# Canine colostrum exosomes: characterization and influence on the canine mesenchymal stem cell secretory profile and fibroblast anti-oxidative capacity

**DOI:** 10.1186/s12917-020-02623-w

**Published:** 2020-11-02

**Authors:** Antonio J. Villatoro, María del Carmen Martín-Astorga, Cristina Alcoholado, José Becerra

**Affiliations:** 1 Laboratory of Bioengineering and Tissue Regeneration (LABRET), Department of Cell Biology, Genetics and Physiology, Faculty of Sciences, University of Málaga, IBIMA, 29071 Málaga, Spain; 2 Instituto de Immunología Clínica y Terapia Celular (IMMUNESTEM), Miraflores del Palo, 14, 29018 Málaga, Spain; 3 Networking Biomedical Research Center in Bioengineering, Biomaterials and Nanomedicine (CIBER-BBN), 28029 Madrid, Spain; 4 Andalusian Centre for Nanomedicine and Biotechnology-BIONAND, Severo Ochoa 35, 29590 Málaga, Spain

**Keywords:** Canine colostrum milk, Mesenchymal stem cells, Exosomes, Dog, Anti-oxidative capacity

## Abstract

**Background:**

Canine colostrum milk (CCM) is a specific secretion of the mammary gland that is fundamental for the survival of the newborn. CCM has many described components (immunoglobulins, proteins or fat), but its small vesicles, named exosomes, are largely unknown.

**Results:**

A characterization of CCM exosomes was performed. Exosomes were abundant in CCM and appeared with the characteristic cup-shaped morphology and well-defined round vesicles. The size distribution of exosomes was between 37 and 140 nm, and western blot analysis showed positive expression of specific exosomal markers. Proteomic analysis revealed a total of 826 proteins in exosome cargo. We also found that exosomes modified the proliferation and secretory profiles in canine mesenchymal stem cells derived from bone marrow (cBM-MSCs) and adipose tissue (cAd-MSCs). Additionally, CCM exosomes demonstrated a potent antioxidant effect on canine fibroblasts in culture.

**Conclusions:**

Our findings highlight, for the first time, the abundant presence of exosomes in CCM and their ability to interact with mesenchymal stem cells (MSCs). The addition of exosomes to two types of MSCs in culture resulted in specific secretory profiles with functions related to angiogenesis, migration and chemotaxis of immune cells. In particular, the cAd-MSCs secretory profile showed higher potential in adipose tissue development and neurogenesis, while cBM-MSC production was associated with immunity, cell mobilization and haematopoiesis. Finally, exosomes also presented antioxidant capacity on fibroblasts against reactive oxygen species activity within the cell, demonstrating their fundamental role in the development and maturation of dogs in the early stages of their life.

**Supplementary information:**

**Supplementary information** accompanies this paper at 10.1186/s12917-020-02623-w.

## Background

Canine colostrum milk (CCM) is a specific secretion of the mammary gland produced during the first two days after labour, and it is fundamental for the survival of dogs during the first weeks after birth [1].

In addition to its nutritional function, CCM plays a very significant role in passive immunity, the development of the immune system and the maturation of various organs, which improves the metabolism and vital functions of the neonate [2–8]. This secretion contains many described components (immunoglobulins, proteins or fat) and different biological membrane structures that transport bioactive molecules (cargo) related to signalling pathways and intercellular communication with newborn tissues [8, 9]. Among these vesicular structures, exosomes stand out.

Exosomes are biological nanovesicles (30–200 nm) composed of a lipid bilayer and secreted by different cell types, whose cargo includes proteins, lipids and nucleic acids (mainly miRNA) [6, 10].

Because of their membrane, exosomes in breast milk can survive harsh conditions, such as digestion, and are absorbed intact by intestinal epithelial cells and incorporated into the circulatory system through vascular endothelial cells [11–15].

Breast milk exosomes are involved in the regulation of the neonate’s immune response, promoting the growth of the intestinal epithelium and microbiota development [16–18].

Exosomes have been described in human breast milk and some domestic species, such as pig, cow, horse, buffalo, yak and camel [19–26]. Although exosomes have been isolated from different cell types in canine species [27–29], they have not been described in CCM.

The effect of CCM exosomes has been evaluated in some cell types [30]; however, it has never been assessed in mesenchymal stem cells (MSCs). MSCs play a strategic role in the development, homeostasis and repair of different organs and tissues [31, 32] and have shown promising results in the treatment of different canine diseases [33, 34]. Therefore, we believe that it is interesting to demonstrate the effect of canine colostrum exosomes on different types of MSCs to help understand their role in the neonatal period in the canine species.

On the other hand, in the early stages of life after birth, there is an exponential increase in reactive oxygen species (ROS) [35], which may be responsible for serious alterations that are very well described in the human neonate [36, 37], calves [38] and canine newborns [35]. Among the components of colostrum, there are different essential antioxidants against oxidative damage [38, 39]; however, the antioxidant potential of CCM exosomes has not been evaluated.

With these premises, the purpose of our study was, for the first time, to isolate exosomes by ultracentrifugation from CCM and characterize them according to transmission electron microscopy (TEM), their size distribution, electronegativity, and exosome markers according to western blot and proteomic analyses.

We also demonstrated the effects of CCM exosomes by evaluating their influence on canine MSC proliferation and their secretory profile.

Finally, we evaluated the antioxidant capacity of CCM exosomes on canine fibroblasts, given that in the early stages of the life of the canine neonate, a large number of ROS-mediated pathologies are related to the maturation of the respiratory system [35], in which fibroblasts play a fundamental role.

## Results

### Colostrum refractive index

The values obtained from all colostrum samples were within the standard values described for this species. The average refractive index value was 1.343 ± 0.0014 (Table 1).

**Table 1:** Information about the donors

Sample	Breed	Age (years)	Weight (Kg)	Puppies	RI
1	Spanish water dog	2	12	4	1.345
2	Yorkshire	4	6	3	1.343
3	Chihuahua	4	5	2	1.343
4	Mixed breed	5	14	6	1.342
5	French bulldog	4	29	7	1.343
6	Golden retriever	3	36	6	1.345
7	Pug	6	11	5	1.341
8	Boxer	3	19	7	1.342
Mean		3.8	16.5	5	1.343
SD		1.25	10.97	1.85	0.0014

The breed, age, weight, number of puppies and refractive index (RI) are indicated. Data are presented as the mean ± standard deviation (SD).

### Canine colostrum exosome characterization

**Fig. 1:**
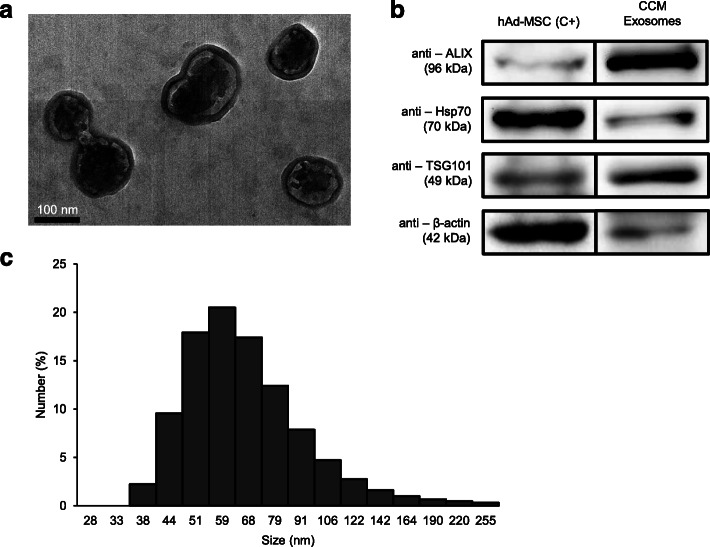
Characterization of CCM exosomes. **a** Representative TEM images of exosomes isolated from CCM. Exosomes appear with characteristic cup-shaped morphology and as round well-delimited vesicles. Bars, 100 nm. **b** Western blot analysis showing positive expression of ALIX, Hsp-70 and TSG-101 surface-specific exosomal markers. Exposure time: 40 s. Positive control: protein lysate of human adipose mesenchymal stem cells (hAd-MSC). Cropped image (original blot included in the Additional file 4). **c** Exosomal size distribution profile

The mean exosome concentration obtained from the eight CCM samples was 305.60 ± 46.7 μg/mL. CCM exosomes were visualized by TEM (Fig. 1a), and their size distribution was between 37 and 140 nm with a zeta potential of − 11.40 ± 0.53 mV (Fig. 1c). The measurement of size was based on the Dynamic Light Scattering (DLS) technique.

CCM exosomes showed positive expression of ALIX, heat shock protein 70 (Hsp70) and TSG101 (tumour susceptibility gene 101) exosomal markers (Fig. 1b and Additional file 4).

### Proteomic analysis

The total number of peptides was determined by mass spectrometry and analysed using the *Canis lupus familiaris* protein database. We found 826 proteins in CCM exosomes. The biological processes of characterized exosome proteins were determined according to *Gene Ontology* (GO) parameters (Additional file 1). CCM exosome proteins are involved in a variety of physiological functions, such as cell differentiation, cell organization and biogenesis, cellular component movement, defence response, metabolic process, regulation of biological process, response to stimulus and transport. Proteins involved in *GO* parameters such as cell communication and conjugation were not found. A list of specific proteins is shown in Additional file 2. One protein can be related to different biological functions.

### Colostrum exosomes significantly increase cAd-MSCs proliferation

**Fig. 2:**
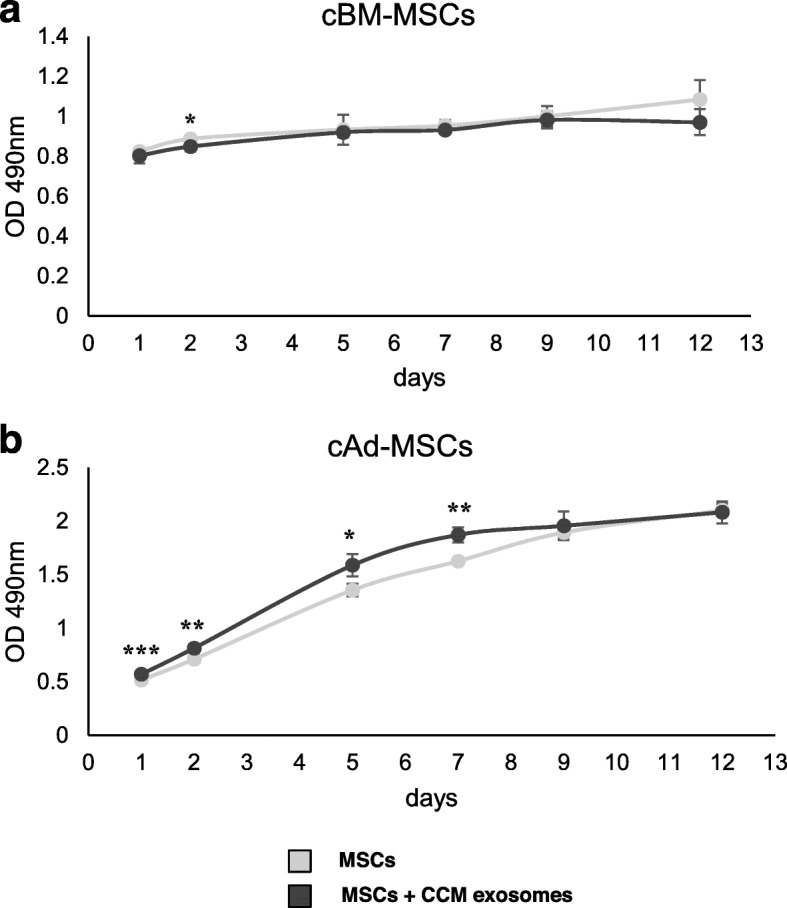
cBM-MSC and cAd-MSC proliferation in the presence of CCM exosomes. Comparison of cBM-MSCs **a** and cAd-MSCs **b** treated with CCM exosomes (dark grey) and their respective control (light grey). Data represent the mean ± SD. Asterisks indicate significant differences between compared values *P* < 0.05 (*), *P* < 0.01 (**) and *P* < 0.001 (***)

The cell proliferation curve showed an increase in cAd-MSCs (Fig. 2b) proliferation for 12 days in the presence of CCM exosomes, whereas this effect was not observed in cBM-MSCs (Fig. 2a).

### Secretory profile of canine MSCs in the presence of colostrum exosomes

**Fig. 3:**
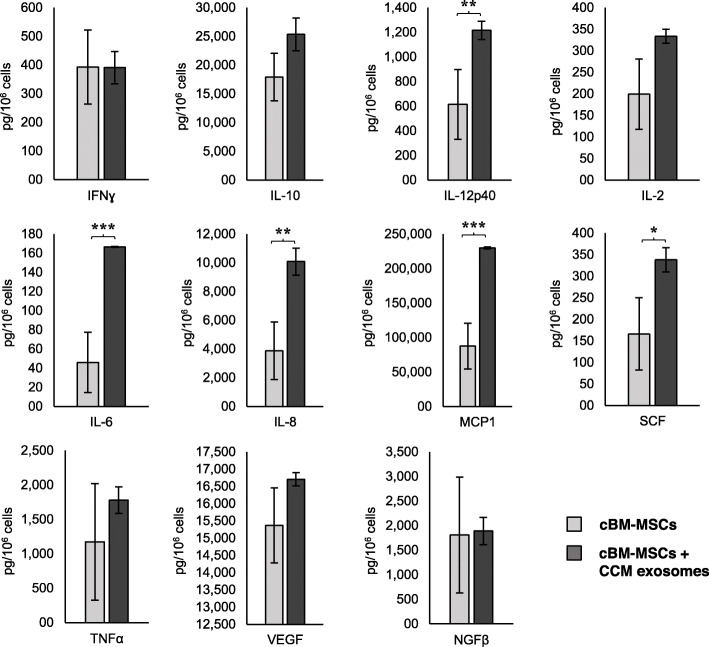
Cytokines and growth factor secretory profile of cBM-MSCs. Controls are indicated in light grey, and cells treated with CCM exosomes are indicated in dark grey. CCM exosomes showed significantly increased IL-12p40, IL-6, IL-8, MCP-1 and SCF production. Asterisks indicate significant differences between compared values *P* < 0.05 (*), *P* < 0.01 (**) and *P* < 0.001 (***). Data presented as the mean and standard deviation (*n* = 3)

**Fig. 4:**
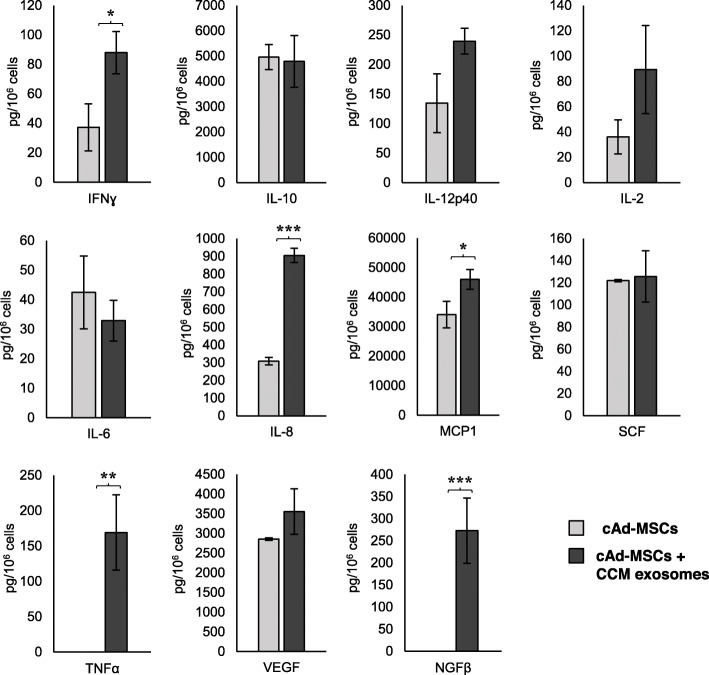
Cytokine and growth factor secretory profile of cAd-MSCs. Controls are indicated in light grey, and cells treated with CCM exosomes are indicated in dark grey. IFN-γ, IL-8, MCP-1, TNF-α and NGF-β production increase after incubation with CCM exosomes. Asterisks indicate significant differences between compared values *P* < 0.05 (*), *P* < 0.01 (**) and *P* < 0.001 (***). Data presented as the mean and standard deviation (*n* = 3)

The secretory profile characterization of both MSC sources is shown in Figs. 3 and 4. After incubation with CCM exosomes, the results showed a production significantly increased of IL-12p40, IL-6, IL-8, MCP-1 and SCF in cBM-MSCs and IFN-γ, IL-8, MCP-1, TNF-α and NGF-β in cAd-MSCs. NO production and IDO activity were not observed in any case.

### Canine colostrum exosomes demonstrate antioxidant capacity in canine fibroblasts

**Fig. 5:**
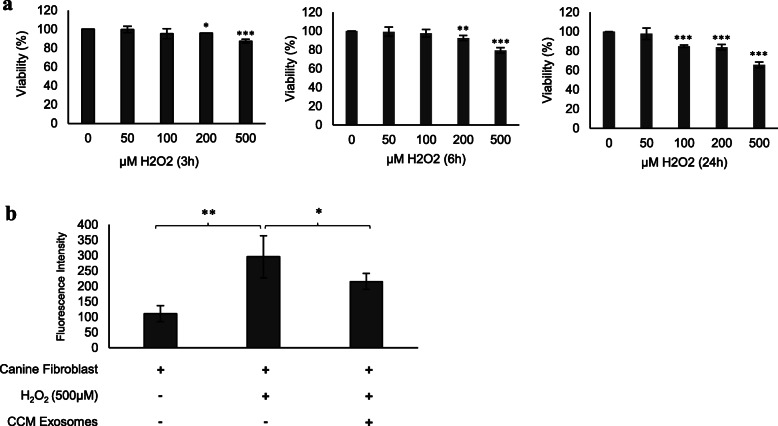
CCM exosomes decrease ROS production in canine fibroblasts. **a** Canine fibroblast cell viability after exposure to 50,100,200 and 500 μM H_2_O_2_ for 3 h, 6 h and 24 h. **b** Fluorescence intensity equivalent to ROS generation by canine fibroblasts according to the applied treatment. Asterisks indicate significant differences between compared values *P* < 0.05 (*), *P* < 0.01 (**) and *P* < 0.001 (***). Data are expressed as the mean ± SD

Canine fibroblasts incubated with H_2_O_2_ were used as a model for ROS overproduction. Cell viability decreased with increasing concentration and exposure time to H_2_O_2_ (Fig. 5a). In the final assay, canine fibroblasts were incubated for 3 h with 500 μM H_2_O_2_, and CCM exosomes were added immediately after incubation. An important decrease in ROS levels was observed in cells treated with CCM exosomes, demonstrating their antioxidant effect (Fig. 5b).

## Discussion

Colostrum plays a fundamental role in survival in the neonatal period of the dog, as well as in its future development as an adult [1, 3]. To date, despite knowledge of the nutritional and immunological components of colostrum in dogs [4, 5], there has been no study on biological nanostructures, such as exosomes, their cargo and biological functions.

As far as we know, this is the first study that describes and characterizes the presence of exosomes from CCM and evaluates their interaction with canine MSCs and fibroblasts.

Ultracentrifugation techniques allowed the isolation of abundant exosomes from CCM, similar to that already described for exosomes isolated from other canine species [27, 29]. The presence of CCM exosomes was confirmed by TEM, size determination and western blot analysis of the expression ALIX, Hsp70 and TSG101 exosomal markers, according to the recommendation of the International Society for Extracellular Vesicles [40, 41].

Exosomes play a key role in cell-to-cell communication and contain different specific proteins depending on their cellular origin. Nevertheless, exosomes share a subset of essential proteins for vesicular biogenesis, structure and distribution [34, 42, 43]. Through proteomic analysis, we identified 892 proteins mainly related to functions such as transport, metabolism, regulation of different biological functions, cell differentiation, organization and biogenesis. These results coincide with colostrum milk exosomes of other species [7, 43], which suggest the evolutionary importance of these vesicles in regulating different cellular functions in newborns [6, 13, 14, 25, 44], which is shared between different species of mammals [10].

When we compared the canine proteomic profile of colostrum exosomes with exosomes from different canine mesenchymal sources already described by our team [29], we found that they share 11 proteins with common functions, confirming that exosomes are carriers of certain proteins with basic functions within the same species. Among these proteins, those related to functions such as angiogenesis, growth, inflammation, metabolism and cell signalling stand out (Additional file 3). Undoubtedly, more studies are needed to understand the functioning of exosomes in canine species.

MSCs play a major role in homeostasis and tissue repair; however, very little is known about the factors that may influence them in early neonatal stages. Evidence suggests that the loss or malfunctioning of stem/progenitor cells necessary for normal cell differentiation and tissue repair may underlie the pathobiology of some diseases [45].

Evaluating MSCs as the target of colostrum exosomes, we found interesting results that depend on the cellular source. CCM exosomes co-cultured with MSCs demonstrated a statistically significant increase in cAd-MSC proliferation, whereas this effect was not observed in cBM-MSCs.

We suggest that colostrum exosomes can play a very interesting role in the development of fat reserves in dog. The percentage of adipose tissue in newborns is low and increases rapidly during the first month of life, a critical process for avoiding the risk of neonatal mortality, which does not appear to be related to breeding size [1, 46, 47].

Adipose tissue, besides being an energy reservoir, represents a natural defence against hypothermia and fulfils metabolic, endocrine and regulatory functions, both with systemic and local effects [48, 49]. MSCs are exerted through a large diversity of secreted adipokines with complex autocrine and paracrine effects [50]. MSCs are multipotent postnatal progenitors, with adipose tissue being the main source of this cell type [29, 51]. MSC fat residents are generally the principal source of adipocytes during postnatal growth and the maintenance of adipose tissue [52]. Therefore, the increased proliferation of MSCs would help increase fat reserves.

In this study, we demonstrated that canine colostrum exosomes lead to changes in the secretory profile of both types of canine MSCs studied, but in a very different way. Of the 13 analytes evaluated, we found a significant increase in the production of 5 of analytes in cAd-MSCs (IL-8, MCP-1, IFN-γ, TNF-α and NGF-β) and 5 analytes in cBM-MSCs (IL-12p40, IL-6, IL-8, MCP-1 and SCF).

Both cell types showed an increase in the secretion of IL-8 and MCP-1, which are factors related to migration, chemotaxis and angiogenesis.

IL-8, also known as CXCL8, has been shown to have potent pro-angiogenic properties, promoting vein endothelial cell proliferation, migration, tube formation and the ability to attract and activate neutrophils [53]. MCP-1, one of the factors associated with the immunomodulatory effects of MSCs, reduces apoptosis and plays a direct mediating role for angiogenesis, which is manifested by the formation of new blood vessels [54] that are necessary for the development and growth process.

cBM-MSCs stimulated with CCM exosomes specifically increase the production of factors related to immunity (IL-6, IL-12p40) and regulation and the mobilization of haematopoiesis (SCF)**.** IL-6 is a pleiotropic cytokine with a key role in different biological processes, such as regulation of the immune response, inflammation, haematopoiesis, apoptosis, cell survival and cell proliferation [55]. IL-12p40 plays an important role in the development of T cells and enhances the production of immune factors [56].

In contrast to cBM-MSCs, colostrum exosomes in cAd-MSCs, in addition to stimulating their proliferation, demonstrated a change in their secretory profile by increasing the release of proinflammatory cytokines (TNF-α and IFN-γ). TNF-α is a pleiotropic cytokine with important but sometimes contradictory functions in numerous physiological processes related to immunity and inflammation [57]. IFN-γ intervenes in macrophage activation, induces the expression of MHC class II molecules, increases cytotoxic potential and favours, together with TNF-α, the development of the fundamental Th1 cell responses to control viral infections [58, 59].

In addition, we found that colostrum exosomes increased the secretion of factors related to neurogenesis (NGF-β), most notably in cAd-MSCs. NGF plays a crucial role in the peripheral and central nervous systems; regulates the growth, differentiation and survival of neurocytes; improves cognitive functions; and shows potential to induce angiogenesis under physiological and pathological conditions [60, 61].

Although both MSC types demonstrate secretory similarity in terms of their functions related to angiogenesis, migration and chemotaxis of immune cells, the different behaviour of each cell type would confirm the importance of their cellular niche in the different biological functions of individuals. Thus, while adipose tissue MSCs show important endocrine and metabolic potential in adipose tissue development and neurogenesis, the response of BM-MSCs is more consistent with immunity, cell mobilization, angiogenesis and haematopoiesis.

Newborns, because of their immature antioxidant capacity, are more prone to oxidative stress than adults [35, 62–64], leading to an increase in the risk factors that trigger inflammation, infection and ischemia and resulting in damage to multiple organs, which plays a key role in the pathogenesis of several perinatal diseases [65–67].

Fibroblasts are an abundant cell type in the body, and their role is to produce the extracellular matrix necessary for the formation and maintenance of structural integrity at very important stages in the maturation of certain vital organs, such as the lung [68, 69]; therefore, they suffer the effects of free radicals. This is the justification for using this cell type to evaluate the antioxidant capacity of CCM exosomes.

Colostrum is known to be essential in the antioxidant mechanism of the neonate [70–73]; however, to date, the antioxidant potential of canine colostrum exosomes against fibroblasts has not been described. We demonstrate the important role that exosomes play in avoiding the effects of free radicals on fibroblasts and intervene in the maturation and development of the puppy.

Therefore, the results presented in our study aid in understanding how colostrum functions through its exosomes, its interrelationship with MSC and its antioxidant role [8, 16, 42].

Although our study obviously had limitations due to the small sample size of colostrum donors and the restrictions posed by the lack of specific reagents available for the canine species, we believe that our work is the first step in this direction. However, a more in-depth investigation of exosome functions with a focus on miRNA cargos, gene regulation, immunity and metabolism may be an interesting line of research.

## Conclusions

We described for the first time the isolation and characterization of exosomes from CCM. Our findings highlight their abundant presence in colostrum and their action on different biological functions. On the one hand, exosomes interact with MSCs by inducing proliferation and modulation of the secretory profile depending on their source.

For the two types of MSCs studied, the addition of exosomes resulted in a secretion profile with functions related to angiogenesis, migration and chemotaxis of immune cells. However, independently, mesenchymal stem cells from adipose tissue showed higher potential in adipose tissue development and neurogenesis, while mesenchymal stem cells from bone marrow showed higher potential in immunity, cell mobilization and haematopoiesis.

On the other hand, exosomes also exert their antioxidant capacity on fibroblasts against the negative effects of free radicals, demonstrating their fundamental role in the development and maturation of dogs in the early stages of their life.

## Methods

All animal procedures were conducted by licensed veterinary surgeons and complied according to both national and European legislation (Spanish Royal Decree RD1201/2005 and EU Directive 86/609/CEE as modified by 2003/65/CE, respectively) for the protection of animals used for research experimentation and other scientific purposes. Likewise, the protocols were approved by the Institutional Animal Care and Use Committee of BIONAND (Andalusian Center for Nanomedicine and Biotechnology), Málaga, Spain, and written consent was obtained from all dog owners.

### Animals and colostrum sample collection

Eight client-owned healthy bitches of different breeds with a mean age of 3.87 ± 1.25 years and body weight of 16.5 +/− 10.97 kg were selected as colostrum donors. The average litter size was 4.75 ± 1.65 dogs. Animals were up to date with vaccinations and deworming and were fed a dry balanced diet for growing dogs ad libitum.

All animals were clinically examined previously, submitted to haematological and biochemical tests, and did not manifest symptoms of infectious or parasitic diseases. No medication was administered during pregnancy. Colostrum was obtained in an interval that oscillated between parturition and 45 min after parturition, always before suction by the puppies. The mammary glands were disinfected, a massage was performed, and 3 mL of colostrum was collected using a manual milk extraction syringe. Colostrum was collected from all mothers from the two inguinal glands (M-5). None of the animals required any type of anaesthesia or sedation, and they were not sacrificed to obtain colostrum. The samples were stored at 4 °C until analysis.

### Refractive index

The colostrum refractive index was measured in colostrum at room temperature (21 °C) with a handheld refractometer on samples diluted 1:2 in distilled water (Atago, Japan; refractive scale from 1.333 to 1.360) as previously described [4, 74]. All samples were analysed in the same session.

### Colostrum exosome isolation and characterization

CCM from each bitch was centrifuged separately at 13.000 g for 30 min to remove cellular debris and microvesicles. The supernatant was centrifuged once at 100,000 g for 60 min at 4 °C, and then, the exosome pellet was washed three times with phosphate-buffered saline (PBS) at 135,000 g for 90 min at 4 °C using a 70 Ti rotor in an Optima LE-80 K ultracentrifuge (Beckman Coulter). The isolated exosomes were resuspended in PBS and quantified by a bicinchoninic acid (BCA) kit (Thermo Fisher Scientific) according to the manufacturer’s instructions [75].

CCM exosomes from the eight bitches were used for all trials. Exosomal surface proteins were analysed by western blot (WB) analysis as follows: 30 μg of CCM exosomes, previously quantified by the BCA kit, were probed with the mouse antibodies anti-ALIX (Abcam), anti-TSG101 (Abcam), anti-Hsp70 (Santa Cruz Biotechnology) and anti-Actin (Abcam). Appropriate secondary antibodies were used, and signal detection was carried out using enhanced chemiluminescence reagent (ECL, Cell Signaling Technology) and visualized in the ChemiDocTM XRS + system (BioRad) [29, 75]. A protein lysate of human adipose mesenchymal stem cells (hAd-MSCs) was used as a positive control.

To determine the shape and size of the samples, they were analysed by transmission electron microscopy (TEM, Morgagni 268D electron microscope). For this assay, an exosomal fraction was placed on a nickel grid (Aname) and allowed to dry overnight. Images were taken the next day [29].

The size distribution of purified exosomes was determined using a Zetasizer Nano ZS (Malvern Instruments). The Z potential parameters (electronegativity) and size distribution were analysed at 25 °C according to the instructions of the Central Research Support Services (SCAI) of the University of Málaga.

### Proteomic analysis

CCM exosomes were analysed by proteomics following the instructions provided by the SCAI. Proteome Discoverer 2.2 software (Thermo Fisher Scientific) coupled to Sequest HT was used for the identification of proteins. The MS/MS^2^ data were matched against the TrEMBL and SwissProt protein sequence databases and with the biological processes provided by the Gene Ontology database. The following parameters were taken into account: (1) N-terminal acetylation and methionine oxidation as variable modifications, (2) carbamidomethylation of the cysteines as a fixed modification, (3) two missed cleavages by trypsin, (4) significance threshold of 0.05, (5) mass tolerance of 0.02 Da for precursors and fragmented masses, and (6) search in the same database with inverted sequences with identical search parameters (“Peptide decoy”) to estimate the number of false positives using Percolator software [76, 77].

### Canine MSC culture and CCM exosome proliferation effects

Canine bone marrow (cBM-MSCs) and adipose tissue (cAd-MSCs) mesenchymal stem cells from the same donor were isolated and characterized as previously described [29, 33, 34]. Cultures were carried out under standard culture conditions: Dulbecco’s modified Eagle’s medium (DMEM) containing 10% exosome-free fetal bovine serum (FBS), 2.5 mM L-glutamine, 100 U/mL penicillin, 100 μg/mL streptomycin, and 1.25 μg/mL fungizone (all from Sigma-Aldrich). Cells were trypsinized at confluence and cryopreserved in liquid nitrogen. The experiments were carried out on culture passage 3. FBS exosome-free serum was obtained by ultra-centrifugation at 100.000 g for 60 min at 4 °C using a 70 Ti rotor in an Optima LE-80 K ultracentrifuge (Beckman Coulter). The supernatant was collected, and the precipitate (exosomes) was eliminated.

Cell proliferation was measured using the MTS assay (CellTiter 96 Aqueous One Solution Cell Proliferation Assay, Promega) according to the manufacturer’s instructions. cBM-MSCs and cAd-MSCs were seeded at a concentration of 3 × 10^3^ cells per well in a 96-well plate. Two doses of CCM exosomes (25 μg/mL) were administered on days 1 and 6, and the cell culture medium absorbance optical density was measured at 490 nm at 1, 2, 5, 7, 9, and 12 days using a microplate reader (ELx800, BioTek instruments).

### Colostrum exosome effects on the canine MSC secretory profile

cBM-MSCs and cAd-MSCs were seeded at a density of 5 × 10^5^ cells in FT-25 flasks under standard culture conditions and incubated overnight. For the experimental group, CCM exosomes were added at a concentration of 25 μg/mL, and the secretome was collected and filtered after 24 h of co-culture. The control group was performed under standard culture conditions for 24 h. The concentrations of 11 analytes were determined by a Luminex canine cytokine 11-plex assay kit (Thermo Fisher Scientific): chemokine (monocyte chemoattractant protein-1, MCP-1); cytokines (interleukins: IL-2, IL-6, IL-8, IL-10, IL-12p40, tumour necrosis factor alpha: TNF-α, interferon gamma: IFN-γ); immune mediator (prostaglandin E2: PGE2) and growth factors (beta-nerve grown factor: NGF-β, stem cell factor: SCF, transforming growth factor beta: TGF-β, vascular endothelial growth factor A: VEGF-A). All the analyte concentrations are expressed in pg/10^6^ cells.

Indoleamine 2, 3-dioxygenase (IDO) enzymatic activity and NO production were measured spectrophotometrically using kynurenine and a nitrite/nitrate colorimetric assay kit (Roche) according to the manufacturer’s protocol, respectively [29].

### Canine fibroblast viability assay

An MTS assay was used to determine canine fibroblast (Cellider Biotech) cell viability. Fibroblasts (3000 per well) were seeded in a 96-well plate, incubated overnight and treated with different concentrations (50, 100, 200, and 500 μM) of hydrogen peroxide (H_2_O_2_) (Sigma) for 3 h, 6 h and 24 h. Standard culture conditions were used for the control group. At the specified time points, 20 μL of MTS solution (CellTiter 96 Aqueous One Solution Cell Proliferation Assay, Promega) was added to the cells. After 3 h of incubation, optical density values were determined at 490 nm using a microplate reader (ELx800, BioTek instruments). Each group was tested in quadruplicate. The cell proliferation rates of treated cells were calculated as relative values with the control group [78].

### Reactive oxygen species measurement

ROS detection was performed using a DCFDA / H2DCFDA - Cellular ROS Assay Kit (Abcam) according to the manufacturer’s instructions. Canine fibroblasts were co-cultured with exosomes (25 μg/mL) for 24 h after being exposed to H_2_O_2_ (500 μM) for 3 h. Standard culture conditions were used after the H_2_O_2_ treatment for the control group. Then, cells were incubated with 2′, 7′-dichlorofluorescin diacetate (DCFDA, 25 μM, 100 μl/well) for 45 min at 37 °C in the dark. DCFDA, a non-fluorescent compound, is oxidized by ROS into 2′, 7′-dichlorofluorescin (DCF), a highly fluorescent compound. ROS signalling was detected by a fluorescence microplate reader (ELx800, Bio-Tek instruments, Winooski, VT, USA) with excitation and emission wavelengths of 485 nm and 535 nm, respectively. The results were analysed by KC4 software (BioTek Instruments) [78].

### Statistical analysis

Data analysis was performed by SigmaPlot 11.0 software, and each test was repeated on three biological replicates. The data are presented as the mean ± standard deviation (SD). S*tudent’s t-test* was used for the MSC proliferation, canine fibroblast viability and ELISA assay results, and the *P*-value was adjusted using the Bonferroni method for multiple comparisons. The degree of significance was established in the following ranges: *P* < 0.05 (*), *P* < 0.01 (**) and *P* < 0.001(***).

## Supplementary information

**Additional file 1.** Comparison of biological processes of characterized exosome proteins determined by Gene Ontology parameters. One protein can be related to different biological functions.

**Additional file 2.** List of specific proteins in CCM exosomes.

**Additional file 3.** List of specific proteins in common between CCM exosomes and canine MSC exosomes.

**Additional file 4.** Original Western Blot images with different exposure times and their descriptions.

## Data Availability

All data generated or analysed during this study are included in this published article (and its supplementary information files).

## Abbreviations

BCA: Bicinchoninic acid
cAd-MSCs: Canine adipose-derived mesenchymal stem cells
cBM-MSCs: Canine bone marrow-derived mesenchymal stem cells
CCM: Canine colostrum milk
DCF: 2′, 7′-dichlorofluorescin
DCFDA: 2′, 7′-dichlorofluorescin diacetate
DLS: Dynamic light scattering
DMEM: Dulbecco’s modified Eagle’s medium
FBS: Fetal bovine serum
Hsp70: Heat shock protein 70
IDO: Indoleamine 2, 3-dioxygenase
IFN-γ: Interferon gamma
interleukins: IL-2, IL-6, IL-8, IL-10, IL-12p40
MCP-1: Monocyte chemoattractant protein-1
MSCs: Mesenchymal stem cells
NGF-β: Beta-nerve grown factor
PGE2: Prostaglandin E2
ROS: Reactive oxygen species
SCF: Stem cell factor
SD: Standard deviation
TEM: Transmission electron microscope
TGF-β: Transforming growth factor beta
TNF-α: Tumour necrosis factor alpha
TSG101: Tumour susceptibility gene 101
VEGF-A: Vascular endothelial growth factor A
WB: Western blot
