# Supplementary material for: Canine colostrum exosomes: characterization and influence on the canine mesenchymal stem cell secretory profile and fibroblast anti-oxidative capacity
Source: BMC Vet Res. 2020 Nov 2;16:417. doi: 10.1186/s12917-020-02623-w (PMC7607682; doi:10.1186/s12917-020-02623-w)
Supplement: Supplementary file 1 — Additional file 1. Comparison of biological processes of characterized exosome proteins determined by Gene Ontology parameters. One protein can be related to different biological functions. [file 12917_2020_2623_MOESM1_ESM.pdf]

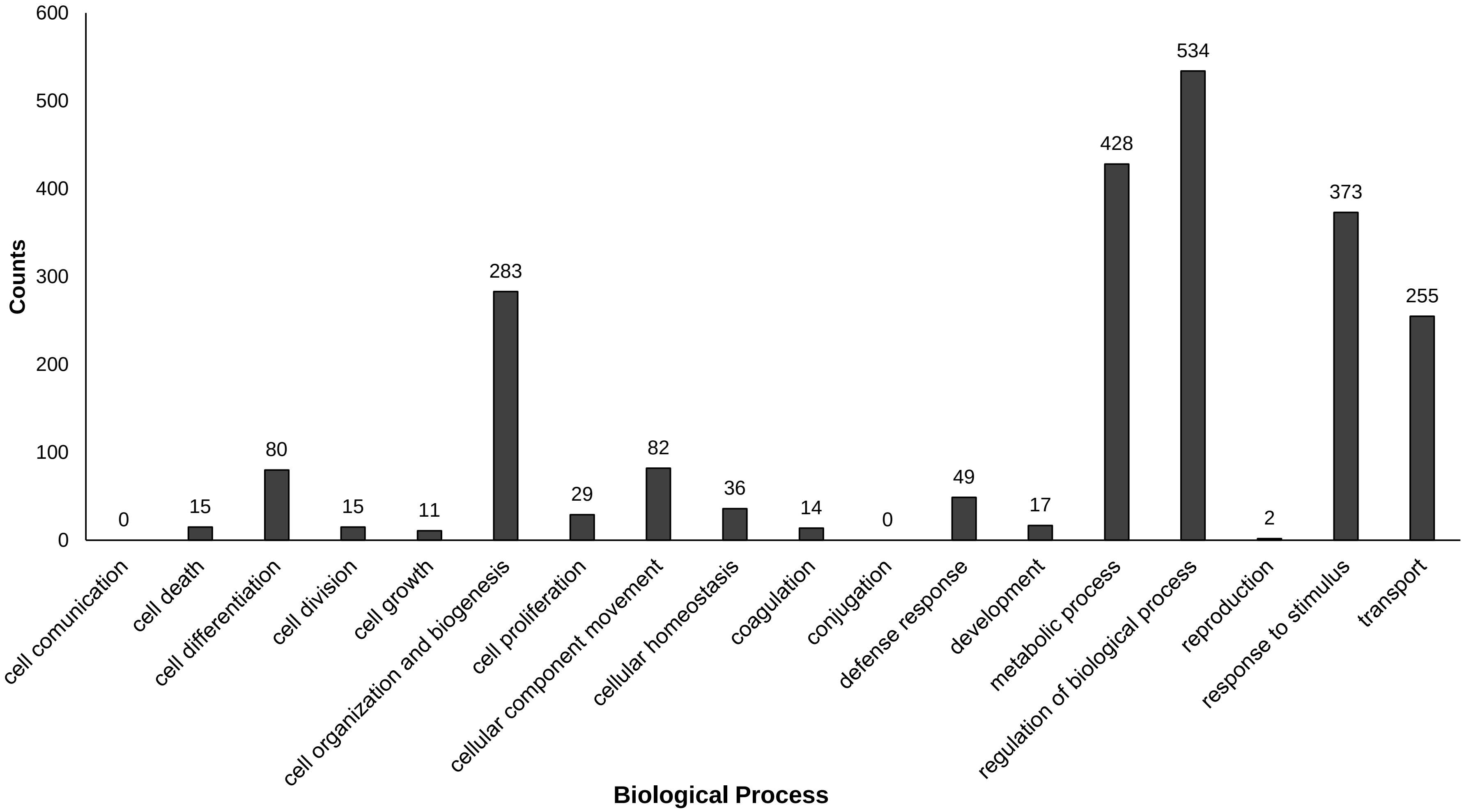

Additional file 1: Comparison of biological processes in characterized exosomes proteins determined by Gene Ontology parameters. One protein can be related with different biological functions
