# Supplementary material for: Canine colostrum exosomes: characterization and influence on the canine mesenchymal stem cell secretory profile and fibroblast anti-oxidative capacity
Source: BMC Vet Res. 2020 Nov 2;16:417. doi: 10.1186/s12917-020-02623-w (PMC7607682; doi:10.1186/s12917-020-02623-w)
Supplement: Supplementary file 2 — Additional file 2. List of specific proteins in CCM exosomes. [file 12917_2020_2623_MOESM2_ESM.pdf]

**Additional file 2:** List of specific proteins in canine colostrum milk exosomes.

| Number | Accession | Description | MW [kDa] | Score | Peptides | Coverage [%] | Biological Process |
|---|---|---|---|---|---|---|---|
| **1** | F1PQT5 | Prominin 2 | 92.2 | 196.379 | 24 | 30 | Regulation of biological process |
| **2** | F1PFZ5 | Milk fat globule-EGF factor 8 protein | 47.8 | 177.433 | 29 | 46 | Cell organization and biogenesis<br>Regulation of biological process |
| **3** | E2R5T9 | Actinin alpha 4 | 104.9 | 175.949 | 35 | 45 | Cell differentiation<br>Cell organization and biogenesis<br>Regulation of biological process |
| **4** | F1PFG6 | Programmed Cell death 6 interacting protein | 96.1 | 175.085 | 39 | 42 | Cell death<br>Cell organization and biogenesis<br>Regulation of biological process |
| **5** | E2QY08 | Actinin alpha 1 | 105.4 | 174.034 | 34 | 43 | Cell differentiation<br>Cell organization and biogenesis<br>Regulation of biological process |
| **6** | F1PIX8 | Complement C3 | 186.8 | 163.806 | 45 | 27 | Cell organization and biogenesis<br>Metabolic process<br>Regulation of biological process<br>Response to stimulus |
| **7** | F1PXU3 | Butyrophilin subfamily 1 member A1 | 58.2 | 161.225 | 26 | 45 | Regulation of biological process |
| **8** | F1PJ65 | IQ motif containing GTPase activating protein 1 | 189 | 145.064 | 36 | 26 | Cell growth<br>Regulation of biological process<br>Response to stimulus |
| **9** | F1PWW0 | Filamin a | 280.5 | 140.499 | 42 | 21 | Cell organization and biogenesis<br>Cellular component movement<br>Regulation of biological process<br>Response to stimulus |
| **10** | F1PSK6 | EH domain containing 1 | 53.1 | 138.514 | 27 | 51 | Cell organization and biogenesis<br>Regulation of biological process<br>Transport |
| **11** | P25473 | Clusterin | 51.8 | 137.516 | 19 | 35 | Metabolic process<br>Regulation of biological process<br>Response to stimulus |

| | | | | | | | |
|---|---|---|---|---|---|---|---|
| **12** | F1Q4J0 | Glucosidase II alpha subunit | 106.3 | 135.039 | 26 | 32 | Metabolic process |
| **13** | P79143 | Aminopeptidase N | 110.2 | 134.516 | 26 | 25 | Cell differentiation<br>Development<br>Metabolic process |
| **14** | E2RCI8 | Annexin | 76.1 | 118.142 | 28 | 42 | Cell organization and biogenesis<br>Cellular homeostasis<br>Regulation of biological process<br>Response to stimulus<br>Transport |
| **15** | F1PDJ5 | Apolipoprotein A-I | 30.2 | 116.208 | 26 | 74 | Cell organization and biogenesis<br>Cell proliferation<br>Cellular component movement<br>Metabolic process<br>Regulation of biological process<br>Response to stimulus<br>Transport |
| **16** | F6X907 | Disco interacting protein 2 homolog B | 171.5 | 107.642 | 31 | 23 | Metabolic process |
| **17** | Q6TEQ7 | Annexin A2 | 38.6 | 101.193 | 20 | 52 | Regulation of biological process |
| **18** | F1PHQ0 | Clathrin heavy chain | 191.5 | 100.585 | 25 | 17 | Cell organization and biogenesis<br>Metabolic process<br>Transport |
| **19** | F1P9J3 | Myosin-9 | 226.3 | 97.815 | 29 | 15 | Cell differentiation<br>Cell organization and biogenesis<br>Cellular component movement<br>Metabolic process<br>Regulation of biological process<br>Transport |
| **20** | J9JHQ2 | Xanthine dehydrogenase | 147.1 | 95.17 | 26 | 18 | Metabolic process |
| **21** | F1PH57 | NCK associated protein 1 | 125.8 | 93.863 | 23 | 21 | Regulation of biological process<br>Response to stimulus |
| **22** | F1PSC1 | Mitogen-activated protein kinase kinase kinase kinase 4 | 151.5 | 93.304 | 24 | 21 | Cell organization and biogenesis<br>Metabolic process<br>Regulation of biological process<br>Response to stimulus |

| | | | | | | | |
|---|---|---|---|---|---|---|---|
| **23** | E2RLS3 | Heat shock protein 90 alpha family class B member 1 | 83.2 | 90.352 | 20 | 28 | Cell organization and biogenesis<br>Metabolic process<br>Regulation of biological process<br>Response to stimulus |
| **24** | F1P6H7 | Fibronectin | 267.1 | 89.515 | 20 | 12 | Cell differentiation<br>Cell organization and biogenesis<br>Metabolic process<br>Regulation of biological process<br>Response to stimulus |
| **25** | F1PP08 | Dipeptidyl peptidase 4 | 88.6 | 88.097 | 22 | 26 | Cellular component movement<br>Defense response<br>Metabolic process<br>Regulation of biological process<br>Response to stimulus |
| **26** | F1P719 | EH domain containing 4 | 61.2 | 86.967 | 22 | 42 | Cell organization and biogenesis<br>Transport |
| **27** | J9NWX6 | Afadin, adherens junction formation factor | 200.6 | 82.854 | 25 | 16 | Regulation of biological process<br>Response to stimulus |
| **28** | E2RD86 | Protein disulfide-isomerase | 56.7 | 81.786 | 22 | 30 | Cellular homeostasis<br>Metabolic process<br>Regulation of biological process<br>Response to stimulus |
| **29** | J9NVU0 | Integrin subunit alpha 6 | 119.4 | 81.191 | 21 | 23 | Regulation of biological process<br>Response to stimulus |
| **30** | F1PR26 | Prostaglandin F2 receptor inhibitor | 97.1 | 80.972 | 23 | 25 | Cell organization and biogenesis |
| **31** | F1PJ74 | Apolipoprotein E | 37.2 | 80.632 | 17 | 49 | Cell organization and biogenesis<br>Cellular homeostasis<br>Metabolic process<br>Regulation of biological process<br>Response to stimulus<br>Transport |
| **32** | F1P9K1 | Immunoglobulin superfamily member 3 | 134 | 79.88 | 24 | 22 | Regulation of biological process<br>Response to stimulus |
| **33** | E2RE76 | Apolipoprotein A-IV | 42.5 | 79.878 | 19 | 48 | Cell organization and biogenesis<br>Defense response<br>Metabolic process |

| | | | | | | | |
|---|---|---|---|---|---|---|---|
| | | | | | | | Regulation of biological process<br>Response to stimulus<br>Transport |
| **34** | E2RSI6 | Ezrin | 69.4 | 78.614 | 24 | 34 | Cell organization and biogenesis<br>Metabolic process<br>Regulation of biological process<br>Response to stimulus<br>Transport |
| **35** | F1PSQ0 | IQ motif containing GTPase activating protein 2 | 178.1 | 77.937 | 27 | 17 | Cell organization and biogenesis<br>Regulation of biological process<br>Response to stimulus |
| **36** | E2RL88 | Integrin subunit alpha 6 | 122 | 76.633 | 22 | 23 | Cell communication<br>Cell differentiation<br>Cell organization and biogenesis<br>Cellular component movement<br>Regulation of biological process<br>Response to stimulus |
| **37** | P18649 | Apolipoprotein E | 35.3 | 76.356 | 16 | 50 | Cell organization and biogenesis<br>Metabolic process<br>Regulation of biological process<br>Response to stimulus;Transport |
| **38** | F1Q147 | Myosin IC | 121.9 | 74.873 | 22 | 22 | Cell organization and biogenesis<br>Regulation of biological process<br>Response to stimulus<br>Transport |
| **39** | E2R434 | Catenin delta 1 | 107.6 | 74.007 | 17 | 19 | Regulation of biological process |
| **40** | P33685 | Beta-lactoglobulin-1 | 18.6 | 70.922 | 8 | 51 | Transport |
| **41** | E2RBB8 | Tyrosine-protein kinase | 58.7 | 70.339 | 17 | 35 | Cell differentiation<br>Cell organization and biogenesis<br>Cellular component movement<br>Defense response<br>Metabolic process<br>Regulation of biological process<br>Response to stimulus<br>Transport |

| | | | | | | | |
|---|---|---|---|---|---|---|---|
| **42** | F1PDQ4 | Cytoplasmic FMR1 interacting protein 1 | 146.1 | 69.753 | 23 | 17 | Cell growth<br>Cell organization and biogenesis<br>Regulation of biological process<br>Response to stimulus |
| **43** | E2RM62 | FAT atypical cadherin 2 | 480.2 | 69.28 | 25 | 6 | Cellular component movement |
| **44** | E2QXN8 | Annexin | 54.4 | 66.892 | 17 | 30 | Response to stimulus<br>Transport |
| **45** | F1PHQ1 | Serpin family A member 9 | 46.9 | 65.715 | 16 | 35 | Regulation of biological process |
| **46** | F1PR93 | WD repeat domain 1 | 66.3 | 65.578 | 13 | 30 | Cell differentiation<br>Cell organization and biogenesis<br>Cellular component movement<br>Metabolic process<br>Regulation of biological process |
| **47** | E2RP20 | Perilipin | 48.9 | 65.386 | 12 | 33 | Transport |
| **48** | J9P829 | Cordon-bleu WH2 repeat protein like 1 | 125.8 | 65.042 | 14 | 17 | Cell organization and biogenesis |
| **49** | F1PRN2 | Myosin Id | 116 | 64.626 | 20 | 21 | Regulation of biological process<br>Transport |
| **50** | E2RTL4 | Procollagen-lysine,2-oxoglutarate 5-dioxygenase 3 | 84.7 | 64.573 | 15 | 22 | Cell organization and biogenesis<br>Metabolic process |
| **51** | F1PN98 | Coagulation factor V | 251.7 | 63.682 | 24 | 10 | Coagulation |
| **52** | F1PTY1 | Keratin, type II cytoskeletal 1 | 63.7 | 63.044 | 14 | 21 | Defense response<br>Metabolic process<br>Regulation of biological process<br>Response to stimulus |
| **53** | J9P969 | AHNAK nucleoprotein | 533.6 | 60.604 | 19 | 19 | Cell organization and biogenesis<br>Regulation of biological process |
| **54** | F1Q0N9 | Keratin 19 | 43.9 | 59.58 | 21 | 53 | Cell differentiation<br>Cell organization and biogenesis<br>Regulation of biological process<br>Response to stimulus |
| **55** | E2RRC6 | Radixin | 71 | 59.543 | 19 | 25 | Cell organization and biogenesis<br>Regulation of biological process<br>Response to stimulus |
| **56** | P41148 | Endoplasmin | 92.5 | 59.321 | 15 | 19 | Metabolic process<br>Response to stimulus |

| | | | | | | | |
|---|---|---|---|---|---|---|---|
| **57** | E2RR68 | Family with sequence similarity 129 member B | 84.3 | 59.015 | 14 | 20 | Metabolic process<br>Regulation of biological process<br>Transport |
| **58** | F1PHR2 | Pyruvate kinase | 59.6 | 57.971 | 11 | 27 | Metabolic process<br>Response to stimulus |
| **59** | Q863H1 | Secreted frizzled-related protein 2 | 33.3 | 57.501 | 12 | 31 | Cell communication<br>Cell differentiation<br>Development<br>Regulation of biological process<br>Response to stimulus |
| **60** | Q9N2G8 | Beta-casein | 28.4 | 56.306 | 6 | 16 | Transport |
| **61** | B6V8E6 | Catenin beta-1 | 85.5 | 53.785 | 13 | 23 | Cell communication<br>Cell differentiation<br>Cell growth<br>Cell organization and biogenesis<br>Cell proliferation<br>Cellular component movement<br>Metabolic process<br>Regulation of biological process<br>Response to stimulus<br>Transport |
| **62** | F1PEN6 | Transferrin receptor protein 1 | 86.5 | 53.77 | 14 | 22 | Cell differentiation<br>Cellular homeostasis<br>Metabolic process<br>Regulation of biological process<br>Response to stimulus<br>Transport |
| **63** | F2Z4N7 | Actin, alpha, cardiac muscle 1 | 42 | 53.672 | 10 | 28 | Cell organization and biogenesis<br>Cellular component movement<br>Regulation of biological process |
| **64** | F1PGY1 | Heat shock protein 90 alpha family class A member 1 | 73 | 53.278 | 14 | 20 | Cell organization and biogenesis<br>Metabolic process<br>Regulation of biological process<br>Response to stimulus<br>Transport |

| | | | | | | | |
|---|---|---|---|---|---|---|---|
| **65** | E2R7F1 | Moesin | 67.9 | 52.768 | 20 | 29 | Cell differentiation<br>Cell organization and biogenesis<br>Cell proliferation<br>Cellular component movement<br>Regulation of biological process<br>Response to stimulus |
| **66** | F6UME0 | Alpha-2-macroglobulin | 165.1 | 52.637 | 16 | 13 | Cell differentiation<br>Regulation of biological process |
| **67** | F1P6B7 | Annexin | 38.6 | 52.305 | 10 | 36 | Cell differentiation<br>Cell organization and biogenesis<br>Cellular component movement<br>Defense response<br>Metabolic process<br>Regulation of biological process<br>Response to stimulus<br>Transport |
| **68** | F1PK62 | Peptidyl-prolyl cis-trans isomerase | 18 | 52.156 | 8 | 49 | Metabolic process |
| **69** | F1PAX2 | Ceruloplasmin | 122.5 | 50.751 | 12 | 12 | Cellular homeostasis<br>Metabolic process<br>Transport |
| **70** | F1PCV3 | Angiotensin-converting enzyme | 151.1 | 50.477 | 19 | 14 | Cell proliferation<br>Metabolic process<br>Regulation of biological process<br>Transport |
| **71** | E2R0F7 | Calpain 7 | 92.7 | 50.044 | 15 | 19 | Metabolic process<br>Regulation of biological process |
| **72** | F1PBA1 | Sodium/potassium-Transporting ATPase subunit alpha | 112.6 | 50.02 | 17 | 17 | Transport |
| **73** | J9NVC6 | Immunoglobulin heavy constant mu | 67.6 | 49.942 | 8 | 12 | Cell organization and biogenesis<br>Defense response<br>Metabolic process<br>Regulation of biological process<br>Response to stimulus |
| **74** | F1PW00 | Protein tyrosine phosphatase, receptor type K | 161.1 | 49.919 | 16 | 13 | Cell organization and biogenesis<br>Cellular component movement<br>Metabolic process |

| | | | | | | | |
|---|---|---|---|---|---|---|---|
| | | | | | | | Regulation of biological process<br>Response to stimulus |
| **75** | F1PKT2 | Lipolysis stimulated lipoprotein receptor | 66.2 | 49.379 | 12 | 26 | Cellular component movement<br>Development<br>Regulation of biological process |
| **76** | J9P3K0 | Myoferlin | 234.4 | 48.259 | 17 | 9 | Cell organization and biogenesis<br>Regulation of biological process<br>Response to stimulus |
| **77** | F1P983 | Protein tyrosine phosphatase, receptor type J | 135.8 | 48.216 | 12 | 13 | Metabolic process<br>Regulation of biological process<br>Response to stimulus |
| **78** | F1Q2F6 | Fatty acid synthase | 273.3 | 47.684 | 18 | 8 | Cell differentiation<br>Metabolic process<br>Response to stimulus |
| **79** | X5IHJ5 | 4F2 Cell-surface antigen heavy chain | 58.4 | 47.26 | 12 | 22 | Metabolic process |
| **80** | F1PV82 | Neural precursor Cell expressed, Developmentally down-regulated 4-like, E3 ubiquitin protein ligase | 110.6 | 47.185 | 14 | 19 | Metabolic process<br>Regulation of biological process<br>Response to stimulus |
| **81** | F2Z4P4 | Elongation factor 1-alpha | 50.1 | 47.116 | 9 | 23 | Metabolic process |
| **82** | F2Z4N2 | Histone H4 | 11.4 | 46.72 | 8 | 53 | Cell organization and biogenesis<br>Metabolic process<br>Regulation of biological process |
| **83** | Q28294 | Guanine nucleotide-binding protein G(Q) subunit alpha | 42.1 | 46.511 | 11 | 33 | Regulation of biological process<br>Response to stimulus |
| **84** | A0A140T8E6 | Junction plakoglobin | 81.7 | 46.186 | 16 | 22 | Cell organization and biogenesis<br>Cellular component movement;<br>Regulation of biological process<br>Response to stimulus |
| **85** | E2RGP6 | Tumor susceptibility 101 | 44.1 | 45.911 | 11 | 29 | Cell differentiation<br>Cell organization and biogenesis<br>Metabolic process<br>Regulation of biological process<br>Transport |

| | | | | | | | |
|---|---|---|---|---|---|---|---|
| **86** | E2RE81 | Protein tyrosine phosphatase, receptor type F | 211.9 | 45.543 | 16 | 10 | Cell organization and biogenesis<br>Cellular component movement<br>Metabolic process<br>Regulation of biological process<br>Response to stimulus |
| **87** | F1PM51 | Epithelial Cell adhesion molecule | 35.3 | 45.286 | 7 | 21 | Cell differentiation<br>Regulation of biological process<br>Response to stimulus |
| **88** | F1PVE5 | Vacuolar protein sorting 4 homolog B | 49.2 | 45.233 | 12 | 27 | Cell organization and biogenesis<br>Metabolic process<br>Regulation of biological process<br>Response to stimulus<br>Transport |
| **89** | F1P6E1 | Complement C1s | 77.5 | 45.144 | 11 | 17 | Defense response<br>Metabolic process<br>Regulation of biological process<br>Response to stimulus |
| **90** | E2R735 | Amine oxidase | 58.5 | 44.719 | 9 | 23 | Metabolic process |
| **91** | E2RB31 | Hypoxia up-regulated 1 | 111.4 | 44.682 | 15 | 15 | Regulation of biological process<br>Response to stimulus<br>Transport |
| **92** | F1P914 | Major vault protein | 99.1 | 44.653 | 16 | 19 | Regulation of biological process<br>Response to stimulus |
| **93** | F2Z4Q6 | Serum albumin | 68.6 | 43.742 | 11 | 17 | Cell communication<br>Regulation of biological process<br>Response to stimulus<br>Transport |
| **94** | E2RAB0 | Chordin like 2 | 47.7 | 42.903 | 7 | 19 | Regulation of biological process |
| **95** | J9P028 | Glutathione peroxidase | 17.3 | 42.806 | 8 | 55 | Cell organization and biogenesis<br>Metabolic process<br>Response to stimulus |
| **96** | E2QXY5 | Erythrocyte membrane protein band 4.1 like 1 | 96.9 | 42.682 | 12 | 17 | Cell organization and biogenesis |
| **97** | F1PYU9 | Keratin, type I cytoskeletal 10 | 57.7 | 41.446 | 13 | 18 | Cell differentiation<br>Metabolic process |

| | | | | | | | |
|---|---|---|---|---|---|---|---|
| **98** | F1PYM4 | Insulin like growth factor binding protein acid labile subunit | 70.3 | 41.217 | 11 | 20 | Regulation of biological process |
| **99** | E2QSF4 | Tubulin beta chain | 49.6 | 40.213 | 8 | 18 | Cell organization and biogenesis |
| **100** | J9P6P4 | Sulfotransferase | 35.3 | 40.044 | 13 | 37 | Metabolic process |
| **101** | E2RJ12 | Sorting nexin | 67.9 | 39.931 | 11 | 19 | Metabolic process<br>Regulation of biological process<br>Transport |
| **102** | O46882 | MHC class I DLA-88 | 40.4 | 39.892 | 7 | 25 | Response to stimulus |
| **103** | F1Q1H6 | Integrin beta | 84.6 | 39.731 | 9 | 14 | Cell differentiation<br>Cell organization and biogenesis<br>Cellular component movement<br>Regulation of biological process<br>Response to stimulus |
| **104** | F1PY11 | Plakophilin 3 | 87.3 | 39.261 | 14 | 19 | Cell organization and biogenesis<br>Regulation of biological process |
| **105** | F1PLP3 | Tetraspanin | 32.6 | 39.093 | 6 | 21 | Regulation of biological process<br>Response to stimulus |
| **106** | A0A068EQ83 | Nectin-4 | 55.1 | 38.509 | 12 | 28 | Transport |
| **107** | F1P9P1 | EPH receptor B3 | 110.1 | 38.383 | 14 | 15 | Cell organization and biogenesis<br>Cellular component movement<br>Metabolic process<br>Regulation of biological process<br>Response to stimulus |
| **108** | F1PWL4 | Nectin Cell adhesion molecule 2 | 57.9 | 38.2 | 9 | 28 | Cell organization and biogenesis<br>Regulation of biological process |
| **109** | F1PQ52 | Myeloperoxidase | 83.6 | 38.199 | 13 | 18 | Cell organization and biogenesis<br>Defense response<br>Metabolic process<br>Regulation of biological process<br>Response to stimulus |
| **110** | E2RPE3 | IST1, ESCRT-III associated factor | 39.9 | 38.001 | 9 | 17 | Cell division<br>Regulation of biological process<br>Transport |

| | | | | | | | |
|---|---|---|---|---|---|---|---|
| **111** | E2R9S7 | Catenin alpha 1 | 100.1 | 37.646 | 13 | 17 | Cell organization and biogenesis<br>Regulation of biological process<br>Response to stimulus |
| **112** | F1PUE2 | Protein kinase C and casein kinase substrate in neurons 2 | 55.9 | 37.637 | 10 | 24 | Cell organization and biogenesis<br>Regulation of biological process<br>Transport |
| **113** | E2RKQ6 | Galectin 3 binding protein | 62.2 | 37.355 | 10 | 17 | Transport |
| **114** | E2RFE1 | Integrin beta | 113.1 | 37.224 | 10 | 12 | Cell organization and biogenesis<br>Cellular component movement<br>Regulation of biological process<br>Response to stimulus |
| **115** | J9NVE9 | Serum amyloid A protein | 14.5 | 37.218 | 5 | 44 | Cellular component movement<br>Defense response<br>Metabolic process<br>Regulation of biological process<br>Response to stimulus |
| **116** | J9P4F3 | Vinculin | 116.7 | 36.901 | 16 | 18 | Cell growth<br>Cell organization and biogenesis<br>Regulation of biological process |
| **117** | E2RD72 | Discs large MAGUK scaffold protein 1 | 108.5 | 36.723 | 12 | 14 | Cell communication<br>Cell organization and biogenesis<br>Cell proliferation<br>Metabolic process<br>Regulation of biological process<br>Response to stimulus |
| **118** | E2RTG5 | Integrin subunit alpha M | 128.2 | 36.597 | 12 | 12 | Cell differentiation<br>Regulation of biological process<br>Response to stimulus |
| **119** | F1PR40 | Pericentrin | 168.6 | 36.578 | 12 | 8 | Metabolic process |
| **120** | O46880 | MHC class I DLA-12 | 39.8 | 36.257 | 7 | 27 | Response to stimulus |
| **121** | P61007 | Ras-related protein Rab-8A | 23.7 | 36.246 | 10 | 43 | Cell organization and biogenesis<br>Metabolic process<br>Regulation of biological process<br>Response to stimulus<br>Transport |

| | | | | | | | |
|---|---|---|---|---|---|---|---|
| **122** | F1PKW7 | Tyrosine 3-monooxygenase/tryptophan 5-monooxygenase activation protein beta | 27.8 | 36.242 | 8 | 32 | Regulation of biological process; Transport |
| **123** | E2R8Z5 | Keratin 5 | 62.7 | 36.228 | 12 | 16 | Metabolic process |
| **124** | M1VKS2 | Disintegrin and metalloproteinase domain-containing protein 10 | 84 | 36.194 | 10 | 15 | Metabolic process<br>Regulation of biological process<br>Response to stimulus |
| **125** | F1PQM1 | Purine nucleoside phosphorylase | 32.3 | 35.873 | 8 | 31 | Metabolic process<br>Regulation of biological process<br>Response to stimulus<br>Transport |
| **126** | F1P9S5 | Plexin B2 | 204.9 | 35.871 | 12 | 6 | Cell proliferation<br>Regulation of biological process<br>Response to stimulus |
| **127** | E2RIV1 | AP complex subunit beta | 105.6 | 35.744 | 11 | 13 | Transport |
| **128** | E2RJH2 | ATPase H+ Transporting accessory protein 2 | 39 | 35.511 | 8 | 22 | Metabolic process<br>Regulation of biological process |
| **129** | E2R1E6 | EPS8 like 2 | 63 | 35.486 | 10 | 16 | Regulation of biological process<br>Response to stimulus |
| **130** | F1PRQ8 | Eph receptor B4 | 108.4 | 34.879 | 10 | 11 | Cellular component movement<br>Metabolic process<br>Regulation of biological process<br>Response to stimulus |
| **131** | E2QWN7 | Lymphocyte cytosolic protein 1 | 70.2 | 34.809 | 12 | 23 | Cell organization and biogenesis<br>Cellular component movement<br>Regulation of biological process<br>Response to stimulus |
| **132** | J9NTN8 | E3 ubiquitin-protein ligase | 98.8 | 34.764 | 9 | 11 | Metabolic process |
| **133** | F6ULX5 | Sphingomyelin phosphodiesterase acid like 3b | 50.7 | 34.758 | 9 | 25 | Metabolic process<br>Regulation of biological process |
| **134** | F1PHH3 | Protein tyrosine kinase 7 (inactive) | 118.4 | 34.74 | 9 | 8 | Cell differentiation<br>Cell organization and biogenesis<br>Cellular component movement<br>Metabolic process |

| | | | | | | | |
|---|---|---|---|---|---|---|---|
| | | | | | | | Regulation of biological process<br>Response to stimulus |
| **135** | F1PLT8 | Sulfhydryl oxidase | 79.1 | 34.669 | 7 | 12 | Cellular homeostasis<br>Metabolic process<br>Regulation of biological process |
| **136** | E2QZC7 | ATP binding cassette subfamily B member 9 | 31.3 | 34.615 | 6 | 30 | Transport |
| **137** | F6V1W9 | Transferrin | 110.4 | 34.417 | 11 | 11 | Regulation of biological process<br>Response to stimulus<br>Transport |
| **138** | F1PZ09 | Misshapen like kinase 1 | 111.1 | 34.235 | 12 | 13 | Cell organization and biogenesis<br>Metabolic process<br>Regulation of biological process |
| **139** | F1PSC2 | Talin 1 | 269.6 | 33.856 | 14 | 6 | Cell organization and biogenesis<br>Regulation of biological process<br>Response to stimulus |
| **140** | F1Q1S4 | Protein tyrosine phosphatase, receptor type C | 147.6 | 33.743 | 10 | 9 | Metabolic process<br>Regulation of biological process<br>Response to stimulus |
| **141** | F1PE28 | Transketolase | 63.1 | 33.708 | 10 | 21 | Metabolic process<br>Regulation of biological process |
| **142** | F1PG39 | Inter-alpha-trypsin inhibitor heavy chain 2 | 107.2 | 33.678 | 13 | 12 | Metabolic process<br>Regulation of biological process |
| **143** | P60524 | Hemoglobin subunit beta | 16 | 33.592 | 8 | 43 | Transport |
| **144** | E2RQ14 | Annexin | 35.9 | 33.592 | 10 | 27 | Regulation of biological process<br>Response to stimulus |
| **145** | O97492 | Catalase | 59.8 | 33.526 | 8 | 17 | Metabolic process<br>Regulation of biological process;Response to stimulus |
| **146** | Q95KM5 | Claudin-3 | 23.1 | 33.372 | 5 | 19 | Cell organization and biogenesis<br>Response to stimulus |
| **147** | J9P7X9 | Gap junction protein | 43 | 33.188 | 5 | 19 | Cell communication<br>Regulation of biological process<br>Response to stimulus |

| | | | | | | | |
|---|---|---|---|---|---|---|---|
| | | | | | | | Transport |
| **148** | J9P2N1 | Matrilin 4 | 69.7 | 33.149 | 12 | 18 | Cell growth |
| **149** | L7N0G4 | Tubulin alpha chain | 50.1 | 32.617 | 9 | 24 | Cell organization and biogenesis |
| **150** | Q28288 | Syntaxin-binding protein 2 | 66.5 | 32.614 | 12 | 24 | Transport |
| **151** | E2RMZ3 | Ferritin | 20 | 32.399 | 6 | 40 | Cellular homeostasis<br>Transport |
| **152** | E2QWX1 | Solute carrier family 15 member 2 | 82.9 | 32.324 | 10 | 13 | Transport |
| **153** | E2RNQ2 | Tubulin alpha chain | 56.7 | 32.228 | 9 | 21 | Cell organization and biogenesis |
| **154** | F1P8G0 | Fibrinogen gamma chain | 49.3 | 31.951 | 8 | 21 | Cell organization and biogenesis<br>Metabolic process<br>Regulation of biological process<br>Response to stimulus<br>Transport |
| **155** | J9P7W6 | Casein alpha s1 | 18.9 | 31.879 | 8 | 47 | Transport |
| **156** | F1PTX4 | Keratin, type II cytoskeletal 2 epidermal | 64.7 | 31.499 | 10 | 12 | Cell organization and biogenesis<br>Cell proliferation<br>Cellular component movement<br>Metabolic process |
| **157** | F1PZW8 | G protein subunit alpha 13 | 34.2 | 31.437 | 10 | 39 | Cell differentiation<br>Regulation of biological process<br>Response to stimulus |
| **158** | J9P922 | Cytochrome b reductase 1 | 31.3 | 31.383 | 6 | 20 | Metabolic process<br>Response to stimulus<br>Transport |
| **159** | F1PBT3 | Fructose-bisphosphate aldolase | 74.9 | 30.622 | 10 | 16 | Metabolic process |
| **160** | E2RNA7 | Chitinase domain containing 1 | 44.9 | 30.591 | 8 | 19 | Defense response<br>Metabolic process<br>Regulation of biological process<br>Response to stimulus |
| **161** | F1PGR0 | ADAM metallopeptidase domain 10 | 85 | 30.419 | 8 | 10 | Metabolic process<br>Regulation of biological process<br>Response to stimulus |
| **162** | E2R0I8 | Glucosylceramidase | 59.6 | 30.404 | 9 | 17 | Cell communication<br>Metabolic process |

| | | | | | | | |
|---|---|---|---|---|---|---|---|
| | | | | | | | Regulation of biological process<br>Response to stimulus |
| **163** | F1PAA1 | Heat shock protein 90 alpha family class B member 1 | 50.1 | 30.352 | 6 | 15 | Transport |
| **164** | E2R1I9 | Hyaluronan and proteoglycan link protein 3 | 41.2 | 30.31 | 7 | 21 | Development<br>Transport |
| **165** | D0VWQ3 | Ribosomal protein L18 | 21.6 | 30.062 | 6 | 29 | Metabolic process |
| **166** | E2RT60 | Integrin beta | 88.1 | 29.795 | 8 | 11 | Cell differentiation<br>Cell growth<br>Cell organization and biogenesis<br>Cellular component movement<br>Metabolic process;<br>Regulation of biological process<br>Response to stimulus<br>Transport |
| **167** | E2R413 | Capping actin protein, gelsolin like | 38.8 | 29.621 | 7 | 19 | Cell organization and biogenesis<br>Regulation of biological process |
| **168** | F1PL97 | Protein disulfide-isomerase | 57.4 | 29.57 | 9 | 16 | Cellular homeostasis<br>Metabolic process<br>Regulation of biological process<br>Response to stimulus |
| **169** | F1PTZ9 | Glyceraldehyde-3-phosphate dehydrogenase | 35.5 | 29.419 | 8 | 30 | Metabolic process |
| **170** | F1Q421 | Plasminogen | 91.1 | 29.339 | 8 | 11 | Cell differentiation<br>Cellular component movement<br>Cellular homeostasis<br>Coagulation<br>Metabolic process<br>Regulation of biological process |
| **171** | E2R761 | L-lactate dehydrogenase | 36.6 | 28.975 | 8 | 26 | Metabolic process |
| **172** | F1Q1C9 | Arrestin domain containing 1 | 46 | 28.969 | 7 | 18 | Metabolic process<br>Regulation of biological process<br>Transport |

| | | | | | | | |
|---|---|---|---|---|---|---|---|
| **173** | D5IGC7 | CD36 antigen | 52.5 | 28.915 | 7 | 10 | Cell organization and biogenesis<br>Cellular homeostasis<br>Defense response<br>Metabolic process<br>Regulation of biological process<br>Response to stimulus<br>Transport |
| **174** | F2Z4P9 | Ras-related protein rab-10 | 22.5 | 28.897 | 10 | 46 | Cell differentiation<br>Cell organization and biogenesis<br>Regulation of biological process<br>Response to stimulus<br>Transport |
| **175** | E2QX93 | Protocadherin 1 | 132.9 | 28.855 | 6 | 7 | Regulation of biological process<br>Transport |
| **176** | F1Q0H3 | Ras-related protein Rab-1A | 27 | 28.689 | 9 | 30 | Cell organization and biogenesis<br>Cellular component movement<br>Defense response<br>Metabolic process<br>Regulation of biological process<br>Response to stimulus<br>Transport |
| **177** | O97556 | Rab GDP dissociation inhibitor beta | 50.3 | 28.629 | 9 | 25 | Metabolic process<br>Regulation of biological process<br>Response to stimulus<br>Transport |
| **178** | F1PEH9 | Acyl-CoA synthetase long chain family member 4 | 74.4 | 28.629 | 8 | 15 | Cell differentiation<br>Metabolic process |
| **179** | B6F250 | Erythrocyte band 7 integral membrane protein stomatin | 31.2 | 28.355 | 9 | 35 | Regulation of biological process |
| **180** | F6Y634 | ATP binding cassette subfamily A member 8 | 184.4 | 27.908 | 11 | 7 | Transport |
| **181** | F6Y3P9 | Gelsolin | 85.2 | 27.845 | 9 | 12 | Cell death<br>Cell organization and biogenesis<br>Metabolic process<br>Regulation of biological process<br>Response to stimulus |

| | | | | | | | |
|---|---|---|---|---|---|---|---|
| | | | | | | | Transport |
| **182** | E2R507 | Fatty acid binding protein 3 | 14.8 | 27.588 | 7 | 53 | Regulation of biological process<br>Transport |
| **183** | F1PQ38 | NADPH oxidase 5 | 86.9 | 27.512 | 11 | 12 | Cell proliferation<br>Metabolic process<br>Transport |
| **184** | F1Q1Y8 | Erythrocyte membrane protein band 4.1 like 4B | 96.2 | 27.5 | 7 | 10 | Cell organization and biogenesis<br>Regulation of biological process<br>Response to stimulus |
| **185** | J9NX68 | Integral membrane protein 2C | 30.1 | 27.416 | 7 | 28 | Cell differentiation<br>Regulation of biological process |
| **186** | F1PA62 | Lipoprotein lipase | 53.1 | 27.107 | 6 | 15 | Cell organization and biogenesis<br>Metabolic process<br>Regulation of biological process<br>Response to stimulus |
| **187** | E2QXD1 | Annexin | 52.5 | 26.844 | 9 | 18 | Cell differentiation<br>Cell proliferation<br>Cellular homeostasis<br>Metabolic process<br>Regulation of biological process<br>Response to stimulus |
| **188** | J9NY98 | Calcium-Transporting ATPase | 102.3 | 26.715 | 10 | 11 | Cell organization and biogenesis<br>Regulation of biological process<br>Transport |
| **189** | Q95MP7 | Ferritin heavy chain | 21.3 | 26.678 | 6 | 28 | Cellular homeostasis<br>Metabolic process<br>Regulation of biological process<br>Response to stimulus<br>Transport |
| **190** | F1PZA1 | Isocitrate dehydrogenase [NADP] | 46.8 | 26.639 | 8 | 21 | Metabolic process;<br>Regulation of biological process<br>Response to stimulus |
| **191** | E2RJJ6 | Metalloendopeptidase | 111.8 | 26.486 | 10 | 11 | Metabolic process<br>Regulation of biological process |

| | | | | | | | |
|---|---|---|---|---|---|---|---|
| **192** | E2RC20 | Nucleoside diphosphate kinase | 32.7 | 26.355 | 5 | 27 | Metabolic process |
| **193** | F1P679 | ARP3 actin related protein 3 homolog | 47.6 | 26.22 | 8 | 26 | Cell division<br>Cell organization and biogenesis<br>Cellular component movement<br>Regulation of biological process |
| **194** | E2R9H5 | Solute carrier family 34 member 2 | 74.8 | 26.144 | 9 | 10 | Cellular homeostasis<br>Response to stimulus<br>Transport |
| **195** | E2R417 | Charged multivesicular body protein 2b | 23.9 | 25.852 | 6 | 23 | Cell organization and biogenesis<br>Cellular homeostasis<br>Regulation of biological process<br>Transport |
| **196** | F1PBI6 | Thrombospondin 1 | 129.5 | 25.677 | 8 | 7 | Cell organization and biogenesis<br>Cellular component movement<br>Defense response<br>Metabolic process<br>Regulation of biological process<br>Response to stimulus |
| **197** | F1PBH0 | Chloride intraCellular channel protein | 28.2 | 25.497 | 5 | 31 | Metabolic process<br>Regulation of biological process<br>Transport |
| **198** | F1PP71 | F11 receptor | 32.7 | 25.42 | 5 | 22 | Cell differentiation<br>Cell organization and biogenesis<br>Regulation of biological process<br>Transport |
| **199** | F2Z4P1 | Tubulin beta chain | 50.4 | 25.091 | 5 | 12 | Cell organization and biogenesis<br>Cellular component movement |
| **200** | F1Q424 | 60S ribosomal protein L6 | 32.6 | 25.059 | 9 | 31 | Cell organization and biogenesis<br>Metabolic process |
| **201** | F1PEE3 | Tight junction protein ZO-2 | 134.5 | 24.794 | 12 | 9 | Regulation of biological process<br>Response to stimulus<br>Transport |
| **202** | J9NRN0 | Chaperonin containing TCP1 subunit 8 | 77.8 | 24.785 | 10 | 15 | Cell organization and biogenesis<br>Metabolic process<br>Regulation of biological process<br>Transport |

| | | | | | | | |
|---|---|---|---|---|---|---|---|
| **203** | E2REK6 | Calmodulin 1 | 16.8 | 24.684 | 8 | 52 | Regulation of biological process<br>Response to stimulus |
| **204** | F1Q1H3 | Programmed Cell death 6 | 21.7 | 24.624 | 5 | 25 | Cell death<br>Cell organization and biogenesis<br>Metabolic process<br>Regulation of biological process<br>Response to stimulus<br>Transport |
| **205** | P38400 | Guanine nucleotide-binding protein G(i) subunit alpha-2 | 40.5 | 24.548 | 6 | 19 | Cell division<br>Cell proliferation<br>Regulation of biological process<br>Response to stimulus |
| **206** | E2RCA0 | 1-acyl-sn-glycerol-3-phosphate acyltransferase | 32.2 | 24.486 | 3 | 18 | Metabolic process<br>Regulation of biological process |
| **207** | E2RIQ2 | Cortactin | 60.5 | 24.484 | 10 | 21 | Cell organization and biogenesis<br>Regulation of biological process<br>Transport |
| **208** | E2R0K4 | RAB14, member RAS oncogene family | 23.9 | 24.473 | 8 | 43 | Cell organization and biogenesis<br>Defense response<br>Regulation of biological process<br>Response to stimulus<br>Transport |
| **209** | F1Q3G6 | Mucin 1, Cell surface associated | 55.7 | 24.308 | 5 | 8 | Regulation of biological process |
| **210** | E2QYG5 | Prolyl 3-hydroxylase family member 4 (non-enzymatic) | 50.4 | 24.265 | 4 | 9 | Cell organization and biogenesis<br>Metabolic process |
| **211** | E2RLB2 | RAS like proto-oncogene B | 23.4 | 24.211 | 9 | 34 | Cell communication<br>Regulation of biological process<br>Response to stimulus |
| **212** | F1PKY2 | Scribbled planar Cell polarity protein | 173.2 | 24.118 | 9 | 6 | Cell communication<br>Cell death<br>Cell organization and biogenesis;Cell proliferation<br>Cellular component movement<br>Metabolic process<br>Regulation of biological process<br>Response to stimulus |

| | | | | | | | |
|---|---|---|---|---|---|---|---|
| | | | | | | | Transport |
| **213** | F1PL36 | BAI1 associated protein 2 | 55 | 24.096 | 8 | 15 | Cell organization and biogenesis<br>Regulation of biological process |
| **214** | E2R0I9 | Glucose-6-phosphate 1-dehydrogenase | 59.6 | 24.008 | 9 | 16 | Metabolic process |
| **215** | D3YJ60 | Chitinase 3-like 1 | 42.6 | 23.961 | 7 | 18 | Defense response<br>Metabolic process<br>Regulation of biological process<br>Response to stimulus<br>Transport |
| **216** | F1PF95 | Alkaline phosphatase | 57.6 | 23.942 | 7 | 17 | Cell differentiation<br>Metabolic process<br>Response to stimulus |
| **217** | E2RKU6 | Gamma-glutamyltransferase 6 | 51.2 | 23.942 | 5 | 13 | Metabolic process |
| **218** | J9JHQ0 | Von Willebrand factor A domain containing 2 | 90.4 | 23.941 | 7 | 10 | Cell organization and biogenesis<br>Regulation of biological process |
| **219** | F1PAV5 | Toll-like receptor 2 | 90.2 | 23.926 | 8 | 9 | Defense response<br>Metabolic process<br>Regulation of biological process<br>Response to stimulus<br>Transport |
| **220** | E2QVP6 | Tyrosine 3-monooxygenase/tryptophan 5-monooxygenase activation protein gamma | 28.2 | 23.917 | 6 | 19 | Regulation of biological process<br>Transport |
| **221** | F1PHQ7 | Calcium-Transporting ATPase | 133.8 | 23.908 | 8 | 7 | Cellular component movement<br>Cellular homeostasis<br>Regulation of biological process<br>Response to stimulus<br>Transport |
| **222** | J9P5N6 | ADP ribosylation factor 4 | 20.5 | 23.827 | 5 | 31 | Cellular component movement<br>Metabolic process<br>Regulation of biological process |

| | | | | | | | |
|---|---|---|---|---|---|---|---|
| | | | | | | | Response to stimulus<br>Transport |
| **223** | P18470 | DLA class II histocompatibility antigen, DR-1 beta chain | 30.1 | 23.734 | 6 | 23 | Response to stimulus |
| **224** | F2Z4Q1 | 40S ribosomal protein S4 | 29.6 | 23.623 | 9 | 27 | Metabolic process |
| **225** | F2Z4Q5 | 40S ribosomal protein S3a | 29.9 | 23.535 | 6 | 25 | Cell differentiation<br>Metabolic process<br>Regulation of biological process |
| **226** | P60529 | Hemoglobin subunit alpha | 15.2 | 23.438 | 5 | 41 | Transport |
| **227** | E2RH80 | Platelet derived growth factor receptor like | 42.1 | 23.414 | 6 | 19 | Cell growth<br>Cell division |
| **228** | F1P699 | Phosphatidylethanolamine-binding protein 1 | 22.4 | 23.294 | 6 | 41 | Development<br>Regulation of biological process |
| **229** | P63091 | Guanine nucleotide-binding protein G(s) subunit alpha | 45.6 | 23.146 | 7 | 19 | Regulation of biological process<br>Response to stimulus |
| **230** | J9NSZ5 | G protein subunit alpha 14 | 41.5 | 23.135 | 7 | 20 | Regulation of biological process<br>Response to stimulus |
| **231** | O97578 | Dipeptidyl peptidase 1 | 49.4 | 23.119 | 9 | 15 | Metabolic process |
| **232** | F1PLT7 | Protein tweety homolog | 54.3 | 23.085 | 6 | 13 | Transport |
| **233** | F1PML0 | Kinesin-like protein | 110.3 | 23.008 | 9 | 10 | Cell organization and biogenesis<br>Cellular component movement<br>Regulation of biological process<br>Transport |
| **234** | F1P8G4 | Fucosidase, alpha-L- 2, plasma | 46.7 | 22.969 | 8 | 23 | Metabolic process<br>Regulation of biological process<br>Response to stimulus |
| **235** | E2RHL4 | ADP ribosylation factor 6 | 20.1 | 22.95 | 4 | 33 | Cell death<br>Cell organization and biogenesis<br>Regulation of biological process<br>Response to stimulus |
| **236** | F1PFB4 | Fermitin family member 2 | 72.4 | 22.777 | 7 | 14 | Cell organization and biogenesis<br>Regulation of biological process<br>Response to stimulus |
| **237** | F1PB77 | Alpha-1,4 glucan phosphorylase | 99.9 | 22.765 | 9 | 12 | Cell death |

| | | | | | | | |
|---|---|---|---|---|---|---|---|
| | | | | | | | Metabolic process |
| **238** | E2RLQ9 | Valosin containing protein | 91 | 22.759 | 10 | 12 | Cell communication<br>Cell organization and biogenesis<br>Metabolic process<br>Regulation of biological process<br>Response to stimulus<br>Transport |
| **239** | J9P6N4 | Tyrosine 3-monooxygenase/tryptophan 5-monooxygenase activation protein eta | 28.2 | 22.64 | 6 | 20 | Metabolic process<br>Regulation of biological process<br>Response to stimulus<br>Transport |
| **240** | J9PB81 | Cathepsin G | 27.3 | 22.583 | 6 | 25 | Defense response<br>Metabolic process<br>Regulation of biological process<br>Response to stimulus |
| **241** | J9NS24 | Glycerol-3-phosphate dehydrogenase [NAD(+)] | 45.8 | 22.414 | 7 | 18 | Metabolic process<br>Regulation of biological process |
| **242** | J9P9Z6 | Growth factor receptor bound protein 2 | 25.2 | 22.157 | 7 | 29 | Cell differentiation<br>Cell organization and biogenesis<br>Cellular component movement<br>Defense response<br>Metabolic process<br>Regulation of biological process<br>Response to stimulus<br>Transport |
| **243** | E2RD95 | G protein subunit beta 2 | 37.3 | 22.143 | 6 | 16 | Regulation of biological process<br>Response to stimulus |
| **244** | I4IY07 | Folate hydrolase 1 | 87.3 | 22.082 | 6 | 9 | Metabolic process |
| **245** | E2RMI3 | Abl interactor 1 | 55.1 | 22.038 | 8 | 21 | Cell organization and biogenesis<br>Metabolic process<br>Regulation of biological process |
| **246** | A0MZ32 | MHC class I antigen | 23.4 | 22.006 | 5 | 28 | Regulation of biological process |
| **247** | F1PAF0 | Tissue alpha-L-fucosidase | 53.9 | 21.992 | 7 | 18 | Metabolic process |
| **248** | E2R302 | Aminopeptidase | 107.2 | 21.864 | 9 | 9 | Cell communication<br>Metabolic process<br>Regulation of biological process |

| | | | | | | | |
|---|---|---|---|---|---|---|---|
| | | | | | | | Response to stimulus |
| **249** | F1P7J4 | Complement C5 | 188.5 | 21.711 | 10 | 5 | Defense response<br>Metabolic process<br>Regulation of biological process<br>Response to stimulus |
| **250** | J9NSZ7 | Myelin protein zero like 1 | 35.9 | 21.634 | 7 | 21 | Regulation of biological process<br>Response to stimulus |
| **251** | E2RRJ6 | AP complex subunit beta | 103.6 | 21.609 | 6 | 8 | Transport |
| **252** | F1Q436 | Protein kinase C and casein kinase substrate in neurons 3 | 48.6 | 21.582 | 7 | 17 | Cell organization and biogenesis<br>Metabolic process<br>Regulation of biological process |
| **253** | E2RMB9 | N-myc downstream regulated gene 1 | 41.7 | 21.543 | 5 | 22 | Cell organization and biogenesis<br>Regulation of biological process<br>Response to stimulus |
| **254** | F1PER6 | Membrane bound transcription factor peptidase, site 1 | 117.3 | 21.411 | 9 | 8 | Cell organization and biogenesis<br>Metabolic process<br>Regulation of biological process |
| **255** | E2RR16 | UDP-glucose glycoprotein glucosyltransferase 1 | 176.4 | 21.397 | 8 | 5 | Metabolic process<br>Regulation of biological process<br>Response to stimulus |
| **256** | F1PS17 | Syntrophin beta 1 | 57.1 | 21.394 | 10 | 18 | Cell organization and biogenesis |
| **257** | E2RRC9 | Phosphoglycerate kinase | 44.4 | 21.374 | 7 | 18 | Metabolic process |
| **258** | P52206 | Guanine nucleotide-binding protein subunit alpha-11 | 23.1 | 21.33 | 5 | 27 | Regulation of biological process<br>Response to stimulus |
| **259** | J9P702 | Guanine nucleotide-binding protein subunit gamma | 8 | 21.264 | 4 | 51 | Regulation of biological process<br>Response to stimulus |
| **260** | J9NSF9 | Prothrombin | 70.3 | 21.263 | 5 | 8 | Cellular homeostasis<br>Coagulation<br>Defense response<br>Metabolic process<br>Regulation of biological process<br>Response to stimulus |
| **261** | E2R9B6 | Fetuin B | 42.3 | 21.242 | 6 | 9 | Regulation of biological process |

| | | | | | | | |
|---|---|---|---|---|---|---|---|
| **262** | F1PWJ7 | Basal Cell adhesion molecule (Lutheran blood group) | 67.9 | 21.183 | 7 | 14 | Cellular component movement |
| **263** | F1Q3J3 | Bactericidal/permeability-increasing protein | 53.2 | 21.102 | 6 | 13 | Defense response<br>Regulation of biological process<br>Response to stimulus |
| **264** | E2QUA6 | Syntaxin binding protein 3 | 67.7 | 21.045 | 9 | 14 | Regulation of biological process<br>Response to stimulus<br>Transport |
| **265** | E2RM32 | LLGL2, scribble Cell polarity complex component | 113.9 | 20.919 | 7 | 8 | Regulation of biological process |
| **266** | E2RSH8 | Tyrosine-protein kinase | 59.9 | 20.672 | 8 | 15 | Cell organization and biogenesis<br>Cellular component movement<br>Defense response<br>Metabolic process<br>Regulation of biological process<br>Response to stimulus |
| **267** | F6XEB5 | RAP1A, member of RAS oncogene family | 21.9 | 20.652 | 5 | 24 | Regulation of biological process<br>Response to stimulus<br>Transport |
| **268** | E2QYG6 | ATPase H+ Transporting V1 subunit A | 68.4 | 20.611 | 6 | 12 | Cellular homeostasis<br>Metabolic process<br>Response to stimulus<br>Transport |
| **269** | E2R2C3 | glucose-6-phosphate isomerase | 62.8 | 20.59 | 7 | 15 | Metabolic process<br>Regulation of biological process |
| **270** | F1PLP6 | SIL1 nucleotide exchange factor | 55.5 | 20.567 | 6 | 13 | Metabolic process |
| **271** | F1PE09 | 6-phosphogluconate dehydrogenase, decarboxylating | 53.1 | 20.191 | 6 | 16 | Metabolic process |
| **272** | F1P6Q0 | G protein subunit alpha i3 | 40.5 | 20.17 | 4 | 14 | Cell division<br>Metabolic process<br>Regulation of biological process<br>Response to stimulus |
| **273** | Q5TJE9 | 40S ribosomal protein S18 | 17.7 | 20.121 | 7 | 43 | Metabolic process |
| **274** | A0A140KFN9 | MHC class II antigen beta chain | 30.2 | 20.101 | 5 | 20 | Response to stimulus |

| | | | | | | | |
|---|---|---|---|---|---|---|---|
| **275** | Q6TN20 | Cathelicidin | 19.4 | 20.059 | 4 | 26 | Defense response<br>Regulation of biological process<br>Response to stimulus |
| **276** | E2R3J1 | eukaryotic translation initiation factor 4A2 | 46.4 | 20.051 | 8 | 23 | Metabolic process<br>Regulation of biological process |
| **277** | E2QW69 | RAB5B, member RAS oncogene family | 23.7 | 20.014 | 6 | 34 | Cell organization and biogenesis<br>Regulation of biological process<br>Response to stimulus<br>Transport |
| **278** | E2RI01 | Solute carrier family 7 member 2 | 71.4 | 19.995 | 5 | 8 | Metabolic process<br>Regulation of biological process<br>Transport |
| **279** | F1PLR3 | G protein-coupled receptor class C group 5 member C | 48.2 | 19.985 | 5 | 14 | Regulation of biological process<br>Response to stimulus |
| **280** | F1PDC7 | Notch 1 | 272 | 19.93 | 7 | 3 | Cell death<br>Cell differentiation<br>Cell division<br>Cell organization and biogenesis<br>Cell proliferation<br>Cellular component movement<br>Defense response<br>Development<br>Metabolic process<br>Regulation of biological process<br>Response to stimulus<br>Transport |
| **281** | G1K2D9 | Haptoglobin | 38.4 | 19.924 | 8 | 20 | Metabolic process<br>Regulation of biological process<br>Response to stimulus |
| **282** | Z4YHH2 | Tripeptidyl-peptidase 1 | 62.3 | 19.909 | 5 | 11 | Cell differentiation<br>Cell organization and biogenesis<br>Metabolic process |
| **283** | E2RAY7 | Target of myb1 like 1 membrane trafficking protein | 52.7 | 19.803 | 7 | 14 | Regulation of biological process<br>Response to stimulus<br>Transport |
| **284** | Q38J75 | Coagulation factor VII | 49.7 | 19.771 | 5 | 12 | Coagulation |

| | | | | | | | |
|---|---|---|---|---|---|---|---|
| | | | | | | | Metabolic process |
| **285** | E2R9N5 | Copine 1 | 59.1 | 19.74 | 5 | 9 | Regulation of biological process<br>Response to stimulus |
| **286** | F1PVW0 | L-lactate dehydrogenase | 39.8 | 19.652 | 5 | 15 | Metabolic process |
| **287** | F1PYQ1 | Ras-related C3 botulinum toxin substrate 1 | 23.5 | 19.626 | 5 | 20 | Cell organization and biogenesis<br>Cellular component movement<br>Regulation of biological process<br>Response to stimulus |
| **288** | E2QZQ6 | Copine 3 | 60.1 | 19.548 | 7 | 14 | Metabolic process<br>Regulation of biological process<br>Response to stimulus |
| **289** | Q5U9D0 | Sodium-glucose coTransporter-like 1 | 72.9 | 19.419 | 5 | 8 | Transport |
| **290** | F1PK43 | Aldo-keto reductase family 1 member A1 | 36.6 | 19.342 | 8 | 23 | Metabolic process |
| **291** | E2RG01 | Complement C7 | 94.9 | 19.141 | 7 | 9 | Response to stimulus |
| **292** | F1PS94 | Notch 2 | 265.2 | 19.12 | 7 | 3 | Cell differentiation<br>Development<br>Regulation of biological process<br>Response to stimulus |
| **293** | F1PCH3 | Enolase 1 | 46.8 | 19.096 | 7 | 14 | Metabolic process<br>Regulation of biological process<br>Response to stimulus |
| **294** | F1PE67 | Adaptor related protein complex 2 alpha 1 subunit | 108.1 | 18.992 | 7 | 8 | Regulation of biological process<br>Transport |
| **295** | G1K288 | Ras-related protein Rab-2A | 25.9 | 18.986 | 6 | 31 | Cell organization and biogenesis<br>Regulation of biological process<br>Response to stimulus |
| **296** | E2R949 | Solute carrier family 44 member 1 | 73.2 | 18.92 | 5 | 9 | Transport |
| **297** | E2R020 | ras homolog family member G | 28.2 | 18.872 | 3 | 19 | Cell organization and biogenesis<br>Cellular component movement<br>Regulation of biological process<br>Response to stimulus |
| **298** | F1PWQ1 | ATPase H+ Transporting accessory protein 1 | 51.5 | 18.867 | 4 | 11 | Cellular homeostasis<br>Regulation of biological process<br>Response to stimulus |

| | | | | | | | |
|---|---|---|---|---|---|---|---|
| | | | | | | | Transport |
| **299** | F1PQN5 | cofilin 1 | 18.5 | 18.866 | 4 | 32 | Cell organization and biogenesis<br>Regulation of biological process<br>Response to stimulus |
| **300** | E2RRT3 | RAB8B, member RAS oncogene family | 23.7 | 18.866 | 6 | 23 | Cell organization and biogenesis<br>Regulation of biological process<br>Response to stimulus<br>Transport |
| **301** | E2R5R9 | UDP-glucose pyrophosphorylase 2 | 56.9 | 18.817 | 7 | 14 | Metabolic process |
| **302** | E2RE16 | p21 (RAC1) activated kinase 4 | 64.1 | 18.77 | 7 | 14 | Cell organization and biogenesis<br>Cellular component movement<br>Metabolic process<br>Regulation of biological process<br>Response to stimulus |
| **303** | E2R2K4 | RAP1B, member of RAS oncogene family | 20.8 | 18.662 | 5 | 24 | Cell proliferation<br>Regulation of biological process<br>Response to stimulus |
| **304** | J9NTD9 | Histone H2B | 19.9 | 18.594 | 5 | 21 | Cell organization and biogenesis<br>Transport |
| **305** | J9NX28 | RAS like proto-oncogene A | 23.5 | 18.581 | 6 | 28 | Cell organization and biogenesis<br>Regulation of biological process<br>Response to stimulus |
| **306** | F1PUM6 | Vitronectin | 53.7 | 18.57 | 5 | 12 | Cell differentiation<br>Cell organization and biogenesis<br>Regulation of biological process<br>Response to stimulus<br>Transport |
| **307** | F1PAA9 | Cadherin-1 | 97.7 | 18.508 | 7 | 9 | Cell organization and biogenesis<br>Regulation of biological process<br>Response to stimulus |
| **308** | F1Q4I9 | Galactosylgalactosylxylosylprotein 3-beta-glucuronosyltransferase | 37 | 18.32 | 5 | 16 | Metabolic process<br>Regulation of biological process |
| **309** | F1PXK3 | Nidogen 1 | 136.3 | 18.182 | 7 | 6 | Cell organization and biogenesis<br>Regulation of biological process |
| **310** | J9NTX5 | Cartilage associated protein | 51.8 | 18.126 | 6 | 13 | Metabolic process |

| | | | | | | | |
|---|---|---|---|---|---|---|---|
| | | | | | | | Regulation of biological process |
| **311** | Q50KA9 | Nucleoside diphosphate kinase A | 17.2 | 17.91 | 3 | 28 | Cell differentiation<br>Metabolic process<br>Regulation of biological process<br>Transport |
| **312** | E2RQE7 | NSF attachment protein alpha | 33.2 | 17.902 | 6 | 21 | Cell communication<br>Cell differentiation<br>Cell organization and biogenesis<br>Regulation of biological process<br>Transport |
| **313** | E2QZ50 | Adenylyl cyclase-associated protein | 51.4 | 17.745 | 6 | 17 | Cell organization and biogenesis<br>Regulation of biological process<br>Response to stimulus |
| **314** | M1VPL1 | Nicastrin | 78.2 | 17.697 | 6 | 8 | Metabolic process |
| **315** | P18067 | ras-related protein Rab-7a | 23.5 | 17.637 | 6 | 27 | Cell organization and biogenesis<br>Cellular homeostasis<br>Metabolic process<br>Regulation of biological process<br>Response to stimulus<br>Transport |
| **316** | P01785 | Ig heavy chain V region MOO | 12.7 | 17.61 | 2 | 23 | Defense response<br>Regulation of biological process<br>Response to stimulus |
| **317** | E2RMM2 | C1GALT1 specific chaperone 1 | 36.4 | 17.577 | 5 | 18 | Cell organization and biogenesis<br>Metabolic process |
| **318** | F1PLS1 | Epidermal growth factor receptor pathway substrate 8 | 91.7 | 17.544 | 4 | 6 | Cell organization and biogenesis<br>Cellular component movement<br>Regulation of biological process<br>Response to stimulus |
| **319** | F1PXG4 | Annexin | 35.9 | 17.501 | 6 | 18 | Cell differentiation<br>Regulation of biological process<br>Response to stimulus |
| **320** | F1PGY6 | Toll interacting protein | 30.6 | 17.498 | 5 | 20 | Cell differentiation<br>Metabolic process<br>Regulation of biological process<br>Response to stimulus |

| | | | | | | | |
|---|---|---|---|---|---|---|---|
| **321** | F1PAG6 | ARP2/3 complex 34 kDa subunit | 20.7 | 17.454 | 4 | 24 | Cell organization and biogenesis<br>Regulation of biological process |
| **322** | F1PXZ9 | KRAS proto-oncogene, GTPase | 21.4 | 17.448 | 5 | 31 | Cell communication<br>Cell differentiation<br>Cell organization and biogenesis<br>Regulation of biological process<br>Response to stimulus |
| **323** | E2RMT4 | Actin related protein 2/3 complex subunit 1B | 41 | 17.33 | 6 | 19 | Cell organization and biogenesis<br>Regulation of biological process |
| **324** | F1Q3Y0 | Profilin | 10.6 | 17.315 | 5 | 64 | Cell organization and biogenesis<br>Regulation of biological process |
| **325** | E2RES2 | Serpin family C member 1 | 52.4 | 17.257 | 4 | 8 | Regulation of biological process |
| **326** | F1PZH2 | Leucyl and cystinyl aminopeptidase | 118.7 | 17.065 | 6 | 7 | Cell communication<br>Metabolic process<br>Regulation of biological process<br>Response to stimulus |
| **327** | F6PME1 | Galectin | 31 | 17.008 | 5 | 20 | Cell differentiation<br>Cellular component movement<br>Regulation of biological process<br>Response to stimulus |
| **328** | E2RJE4 | Cystatin | 16.1 | 16.899 | 3 | 28 | Regulation of biological process |
| **329** | F1PHN5 | G protein-coupled receptor class C group 5 member A | 40.1 | 16.894 | 4 | 9 | Regulation of biological process<br>Response to stimulus |
| **330** | F1PQ79 | Adaptor related protein complex 2 mu 1 subunit | 49.6 | 16.888 | 8 | 14 | Transport |
| **331** | E2RM30 | Tissue specific transplantation antigen P35B | 35.8 | 16.858 | 7 | 26 | Metabolic process |
| **332** | E2R273 | SH3 domain containing GRB2 like 1, endophilin A2 | 43.5 | 16.846 | 5 | 14 | Cell organization and biogenesis<br>Transport |
| **333** | A0A096P6K5 | Serum amyloid A protein | 14.3 | 16.839 | 4 | 36 | Defense response<br>Response to stimulus |
| **334** | E2R5G6 | Related RAS viral (r-ras) oncogene homolog | 23.6 | 16.79 | 5 | 32 | Cell differentiation<br>Regulation of biological process<br>Response to stimulus |
| **335** | F1PBM2 | Vesicle trafficking 1 | 33.8 | 16.749 | 5 | 19 | Cell organization and biogenesis |

| | | | | | | | |
|---|---|---|---|---|---|---|---|
| | | | | | | | Transport |
| **336** | E2R5C0 | Glycoprotein M6B | 33.4 | 16.738 | 3 | 7 | Regulation of biological process |
| **337** | Q30343 | Integral membrane glycoprotein | 29.2 | 16.701 | 5 | 18 | Response to stimulus |
| **338** | F1P8D5 | EGF like repeats and discoidin domains 3 | 53.7 | 16.666 | 6 | 14 | Regulation of biological process |
| **339** | F1P841 | GC, vitamin D binding protein | 54.3 | 16.533 | 6 | 10 | Metabolic process<br>Transport |
| **340** | F1PSB7 | Tyrosine-protein kinase | 60.6 | 16.525 | 7 | 11 | Cell differentiation<br>Cellular component movement<br>Defense response<br>Metabolic process<br>Regulation of biological process<br>Response to stimulus<br>Transport |
| **341** | F1Q3Y1 | Peptidyl arginine deiminase 2 | 66.5 | 16.317 | 4 | 8 | Cell organization and biogenesis<br>Metabolic process<br>Regulation of biological process<br>Response to stimulus |
| **342** | F1PGZ8 | G protein-coupled receptor class C group 5 member B | 43 | 16.291 | 4 | 10 | Regulation of biological process<br>Response to stimulus |
| **343** | E2RLQ6 | 6-phosphogluconolactonase | 27.5 | 16.258 | 4 | 22 | Metabolic process |
| **344** | J9P4H9 | Keratin 13 | 49.4 | 16.242 | 7 | 11 | Cell organization and biogenesis |
| **345** | F1Q0R0 | Keratin 14 | 52.3 | 16.168 | 5 | 8 | Cell differentiation<br>Cell organization and biogenesis |
| **346** | A0A1S7J0A8 | Monocyte differentiation antigen CD14 | 39.9 | 16.092 | 4 | 12 | Defense response<br>Response to stimulus |
| **347** | F1PL93 | Rho GDP dissociation inhibitor alpha | 23.4 | 16.049 | 5 | 30 | Regulation of biological process<br>Response to stimulus |
| **348** | O78115 | MHC class II beta chain | 30.7 | 15.969 | 6 | 25 | Response to stimulus |
| **349** | E2QVU9 | Biliverdin reductase B | 22.1 | 15.955 | 5 | 23 | Metabolic process |
| **350** | E2RE22 | Signal transducer and activator of transcription | 90.6 | 15.842 | 6 | 9 | Metabolic process<br>Regulation of biological process<br>Response to stimulus |
| **351** | E2RFV9 | Complement C9 | 62.1 | 15.812 | 5 | 7 | Cell organization and biogenesis<br>Response to stimulus |

| | | | | | | | |
|---|---|---|---|---|---|---|---|
| **352** | E2QWF5 | Peptidylprolyl isomerase | 51.5 | 15.73 | 5 | 12 | Cell organization and biogenesis<br>Metabolic process<br>Regulation of biological process<br>Response to stimulus |
| **353** | F1P9A7 | Protein tweety homolog | 56.8 | 15.712 | 4 | 8 | Transport |
| **354** | E2RB38 | Tropomyosin 3 | 28.9 | 15.708 | 5 | 17 | Cellular component movement |
| **355** | F1PTZ7 | CD44 antigen | 76.9 | 15.658 | 4 | 8 | Cellular component movement<br>Metabolic process<br>Regulation of biological process<br>Response to stimulus |
| **356** | F1PRR4 | Vasodilator-stimulated phosphoprotein | 40.5 | 15.642 | 6 | 17 | Cell organization and biogenesis |
| **357** | F1PJM1 | Fibulin 5 | 50.5 | 15.599 | 4 | 9 | Cell organization and biogenesis<br>Regulation of biological process<br>Transport |
| **358** | E2R774 | Lipase G, endothelial type | 57 | 15.582 | 4 | 11 | Cell organization and biogenesis<br>Metabolic process<br>Regulation of biological process<br>Transport |
| **359** | J9P9U7 | Histone cluster 1 H1 family member e | 21.9 | 15.511 | 5 | 16 | Cell organization and biogenesis<br>Metabolic process<br>Regulation of biological process |
| **360** | E2RNC2 | Claudin | 22.1 | 15.471 | 2 | 13 | Transport |
| **361** | F1PLR0 | Receptor for activated C kinase 1 | 35.1 | 15.408 | 5 | 16 | Cellular homeostasis<br>Metabolic process<br>Regulation of biological process<br>Response to stimulus |
| **362** | F1PRV8 | Deleted in malignant brain tumors 1 | 203.4 | 15.388 | 6 | 7 | Transport |
| **363** | J9P0L5 | Histone H2A | 14.1 | 15.365 | 4 | 35 | Regulation of biological process |
| **364** | J9P5F0 | Complement factor D | 28 | 15.358 | 6 | 25 | Metabolic process |
| **365** | E2RT65 | Phosphoglycerate mutase | 28.8 | 15.356 | 4 | 20 | Metabolic process<br>Regulation of biological process |
| **366** | J9NYU5 | Histone H2B | 17.9 | 15.315 | 4 | 23 | Cell organization and biogenesis |
| **367** | H9GWY1 | Inter-alpha-trypsin inhibitor heavy chain family member 4 | 118.6 | 15.153 | 5 | 4 | Defense response<br>Metabolic process |

| | | | | | | | |
|---|---|---|---|---|---|---|---|
| | | | | | | | Regulation of biological process<br>Response to stimulus |
| **368** | E2QUI7 | EPS8 like 1 | 78.5 | 15.146 | 6 | 11 | Regulation of biological process;<br>Response to stimulus |
| **369** | F1P8Z5 | Apolipoprotein B | 501.2 | 15.081 | 6 | 1 | Cell organization and biogenesis<br>Cellular component movement<br>Metabolic process<br>Regulation of biological process<br>Response to stimulus<br>Transport |
| **370** | E2RNC3 | Dynamin 2 | 98.2 | 15.074 | 6 | 7 | Cell organization and biogenesis<br>Metabolic process<br>Regulation of biological process<br>Transport |
| **371** | F1PPI5 | Amino acid Transporter | 57.5 | 15.071 | 4 | 8 | Transport |
| **372** | H9GW87 | Transaldolase | 37.5 | 15.061 | 6 | 18 | Metabolic process |
| **373** | E2RH47 | 40S ribosomal protein S3 | 26.7 | 14.986 | 6 | 23 | Cell death<br>Cell division;<br>Cell organization and biogenesis<br>Metabolic process<br>Regulation of biological process<br>Response to stimulus |
| **374** | F1PC59 | Peroxiredoxin 6 | 24.7 | 14.893 | 7 | 25 | Cellular homeostasis<br>Metabolic process<br>Regulation of biological process<br>Response to stimulus |
| **375** | F1PQS3 | Capping actin protein of muscle Z-line beta subunit | 41.1 | 14.815 | 6 | 16 | Cell organization and biogenesis<br>Regulation of biological process |
| **376** | E2RR96 | WAS protein family member 2 | 54.3 | 14.787 | 5 | 12 | Cell organization and biogenesis<br>Cellular component movement<br>Regulation of biological process<br>Response to stimulus<br>Transport |
| **377** | F1PUX4 | 60S acidic ribosomal protein P0 | 34.3 | 14.765 | 4 | 13 | Metabolic process<br>Regulation of biological process |
| **378** | E2RLN1 | Serpin family D member 1 | 56.6 | 14.659 | 6 | 10 | Regulation of biological process |

| | | | | | | | |
|---|---|---|---|---|---|---|---|
| **379** | E2RLY5 | Leucine aminopeptidase 3 | 56.2 | 14.552 | 5 | 11 | Metabolic process |
| **380** | E2RB79 | T-complex protein 1 subunit gamma | 61.2 | 14.391 | 5 | 8 | Cell organization and biogenesis<br>Metabolic process<br>Regulation of biological process<br>Transport |
| **381** | F1Q418 | Inter-alpha-trypsin inhibitor heavy chain 1 | 100.9 | 14.385 | 7 | 7 | Metabolic process<br>Regulation of biological process |
| **382** | F1PR99 | Spastin | 68.1 | 14.336 | 7 | 13 | Cell differentiation<br>Cell division<br>Cell organization and biogenesis<br>Cellular component movement<br>Development;Metabolic process<br>Regulation of biological process<br>Response to stimulus<br>Transport |
| **383** | F1PLC3 | Lin-7 homolog A, crumbs Cell polarity complex component | 23.1 | 14.332 | 3 | 16 | Cell organization and biogenesis<br>Transport |
| **384** | A0A0A0MPD0 | Triosephosphate isomerase | 31.7 | 14.276 | 4 | 15 | Development<br>Metabolic process |
| **385** | E2R0L9 | t-complex 1 | 60.2 | 14.224 | 5 | 10 | Metabolic process<br>Regulation of biological process<br>Transport |
| **386** | F6XY66 | Transforming protein RhoA | 21.8 | 14.184 | 4 | 18 | Cell differentiation<br>Cell organization and biogenesis<br>Cellular component movement<br>Regulation of biological process<br>Response to stimulus |
| **387** | E2R2S8 | G protein subunit beta 4 | 37.6 | 14.132 | 5 | 13 | Regulation of biological process<br>Response to stimulus |
| **388** | J9P788 | Signal recognition particle receptor subunit alpha | 83.9 | 14.119 | 5 | 7 | Cell organization and biogenesis<br>Transport |
| **389** | Q95404 | Dqa1 protein | 23.3 | 14.113 | 4 | 23 | Response to stimulus |
| **390** | E2RPW3 | Paraoxonase 1 | 40.1 | 13.978 | 5 | 13 | Metabolic process<br>Regulation of biological process<br>Response to stimulus |

| | | | | | | | |
|---|---|---|---|---|---|---|---|
| **391** | F1PIQ9 | Receptor protein-tyrosine kinase | 138.1 | 13.946 | 5 | 4 | Cell differentiation<br>Cell organization and biogenesis<br>Cellular component movement<br>Metabolic process<br>Regulation of biological process;<br>Response to stimulus |
| **392** | E2RGF3 | Na(+)/H(+) exchange regulatory cofactor NHE-RF | 39.6 | 13.742 | 4 | 14 | Cell differentiation<br>Cell organization and biogenesis<br>Cellular homeostasis;Regulation of biological process<br>Response to stimulus<br>Transport |
| **393** | E2RKC6 | Exostosin glycosyltransferase 2 | 84.1 | 13.654 | 5 | 6 | Cell differentiation<br>Metabolic process |
| **394** | P51147 | Ras-related protein Rab-5C | 23.4 | 13.525 | 4 | 21 | Regulation of biological process<br>Response to stimulus<br>Transport |
| **395** | E2RFR8 | Tyrosine-protein phosphatase non-receptor type | 67.6 | 13.49 | 5 | 8 | Cell differentiation<br>Cell proliferation<br>Defense response<br>Metabolic process<br>Regulation of biological process<br>Response to stimulus |
| **396** | F1PX75 | Protein phosphatase 2 scaffold subunit Aalpha | 65.3 | 13.361 | 4 | 7 | Cell organization and biogenesis<br>Metabolic process<br>Regulation of biological process |
| **397** | F1Q2Y5 | Zymogen granule protein 16B | 20.6 | 13.347 | 2 | 13 | Regulation of biological process |
| **398** | F1PK32 | N-acetyl-alpha-glucosaminidase | 83 | 13.327 | 5 | 8 | Cell organization and biogenesis<br>Response to stimulus |
| **399** | F1PPL6 | RAP2C, member of RAS oncogene family | 20.7 | 13.325 | 5 | 21 | Regulation of biological process<br>Response to stimulus |
| **400** | E2QY64 | Biotinidase | 61 | 13.309 | 4 | 9 | Metabolic process |
| **401** | E2RLG1 | Calcium/calmodulin dependent serine protein kinase | 104.4 | 13.282 | 5 | 6 | Metabolic process<br>Regulation of biological process<br>Transport |

| | | | | | | | |
|---|---|---|---|---|---|---|---|
| **402** | E2QSE3 | Flotillin 1 | 47.3 | 13.23 | 4 | 11 | Cell organization and biogenesis<br>Regulation of biological process<br>Response to stimulus<br>Transport |
| **403** | F1PDB4 | ATP synthase subunit beta | 56.3 | 13.194 | 3 | 7 | Metabolic process<br>Transport |
| **404** | F1PGJ9 | Platelet derived growth factor C | 34.8 | 13.176 | 4 | 10 | Cell organization and biogenesis<br>Coagulation<br>Regulation of biological process<br>Response to stimulus |
| **405** | E2QRX1 | Vacuolar protein sorting-associated protein 35 | 91.6 | 13.136 | 4 | 5 | Cell organization and biogenesis<br>Regulation of biological process<br>Transport |
| **406** | F1PQF9 | Anoctamin | 106 | 13.126 | 6 | 5 | Regulation of biological process<br>Response to stimulus<br>Transport |
| **407** | J9P0B2 | Histone H2A | 14.7 | 13.107 | 4 | 34 | Regulation of biological process |
| **408** | E2QVP0 | Phospholipase D family member 3 | 54.7 | 13.086 | 5 | 11 | Metabolic process |
| **409** | E2QXF3 | 60S ribosomal protein L15 | 24.1 | 13.065 | 5 | 24 | Metabolic process |
| **410** | F1P977 | G protein subunit alpha i1 | 34.8 | 13.03 | 4 | 14 | Cell division<br>Regulation of biological process<br>Response to stimulus |
| **411** | F1PG85 | Solute carrier family 44 member 2 | 80.1 | 13.02 | 5 | 5 | Regulation of biological process<br>Response to stimulus |
| **412** | F1PXM2 | Matrix metalloproteinase | 65.8 | 12.937 | 6 | 10 | Cell differentiation<br>Cell proliferation<br>Cellular component movement<br>Metabolic process<br>Regulation of biological process |
| **413** | E2R141 | Complement C8 beta chain | 67 | 12.914 | 2 | 5 | Response to stimulus |
| **414** | O97758 | Tight junction protein ZO-1 | 197.5 | 12.911 | 5 | 3 | Cell organization and biogenesis<br>Response to stimulus |
| **415** | J9JHN1 | RAB11B, member RAS oncogene family | 24.5 | 12.852 | 5 | 22 | Metabolic process<br>Regulation of biological process<br>Response to stimulus<br>Transport |

| | | | | | | | |
|---|---|---|---|---|---|---|---|
| **416** | F1PFK0 | Twinfilin actin binding protein 1 | 40.8 | 12.845 | 6 | 15 | Cell organization and biogenesis<br>Metabolic process<br>Regulation of biological process |
| **417** | J9NU00 | VPS28, ESCRT-I subunit | 25.9 | 12.822 | 4 | 19 | Metabolic process<br>Regulation of biological process<br>Transport |
| **418** | E2R516 | Synaptosomal-associated protein | 23.4 | 12.798 | 4 | 28 | Cell organization and biogenesis<br>Transport |
| **419** | F6Y2H4 | Serpin family E member 2 | 43.9 | 12.675 | 5 | 11 | Cell organization and biogenesis<br>Coagulation<br>Regulation of biological process<br>Response to stimulus<br>Transport |
| **420** | Q5QQ56 | Xylosyltransferase 1 | 106.9 | 12.66 | 4 | 4 | Metabolic process |
| **421** | E2RP05 | 40S Ribosomal protein S6 | 28.7 | 12.643 | 4 | 18 | Cell death<br>Cell differentiation<br>Cell proliferation<br>Metabolic process<br>Regulation of biological process<br>Response to stimulus |
| **422** | E2RKJ6 | Serpin family B member 1 | 42.8 | 12.631 | 5 | 12 | Regulation of biological process |
| **423** | E2R4F5 | 60S ribosomal protein L13 | 24.3 | 12.587 | 5 | 25 | Metabolic process |
| **424** | F1PEN8 | Carboxylic ester hydrolase | 68.5 | 12.58 | 4 | 7 | Metabolic process |
| **425** | E2RM09 | DnaJ heat shock protein family (Hsp40) member A1 | 44.8 | 12.554 | 4 | 11 | Metabolic process<br>Regulation of biological process<br>Response to stimulus |
| **426** | E2QXN9 | Cystathionine beta-synthase | 60.1 | 12.498 | 5 | 9 | Metabolic process<br>Regulation of biological process<br>Response to stimulus |
| **427** | P56595 | Apolipoprotein C-I | 9.7 | 12.46 | 5 | 36 | Cell organization and biogenesis<br>Metabolic process<br>Regulation of biological process<br>Transport |
| **428** | J9P6V1 | complement C1r subcomponent like | 54 | 12.436 | 4 | 9 | Metabolic process |

| | | | | | | | |
|---|---|---|---|---|---|---|---|
| **429** | E2RFM5 | Mannosyl (alpha-1,3-)-glycoprotein beta-1,2-N-acetylglucosaminyltransferase | 51.6 | 12.404 | 6 | 11 | Metabolic process |
| **430** | E2R8R8 | Ribosomal protein S9 | 22.6 | 12.404 | 5 | 23 | Metabolic process<br>Regulation of biological process |
| **431** | E2QXY7 | ARP2 actin related protein 2 homolog | 45.4 | 12.382 | 4 | 13 | Cell division<br>Cell organization and biogenesis<br>Cellular component movement<br>Regulation of biological process<br>Transport |
| **432** | F1PKR6 | Ribosomal protein S16 | 17.8 | 12.37 | 4 | 23 | Metabolic process |
| **433** | E2RGI4 | chloride intraCellular channel protein | 28.7 | 12.357 | 4 | 23 | Metabolic process<br>Regulation of biological process<br>Transport |
| **434** | F1PMN4 | Sushi repeat containing protein, X-linked 2 | 52.9 | 12.321 | 3 | 8 | Cellular component movement<br>Regulation of biological process<br>Response to stimulus |
| **435** | F1PJP0 | Ectonucleotide pyrophosphatase/phosphodiesterase 1 | 103.9 | 12.276 | 6 | 6 | Cellular homeostasis<br>Metabolic process<br>Regulation of biological process<br>Response to stimulus<br>Transport |
| **436** | F1PQX2 | UEV and lactate/malate dehyrogenase domains | 56.7 | 12.216 | 4 | 8 | Metabolic process<br>Transport |
| **437** | J9JHH9 | Lysophosphatidic acid receptor 1 | 41 | 12.131 | 3 | 12 | Regulation of biological process<br>Response to stimulus |
| **438** | F1P8F7 | Solute carrier family 16 member 7 | 53.1 | 12.09 | 3 | 6 | Transport |
| **439** | E2R985 | Actin-related protein 2/3 complex subunit 3 | 23.3 | 12.074 | 3 | 16 | Cell organization and biogenesis<br>Regulation of biological process |
| **440** | F1PCM5 | Transmembrane protein 8A | 84.4 | 12.042 | 4 | 5 | Regulation of biological process |
| **441** | F1PCK2 | Alpha-1-B glycoprotein | 57.9 | 12.003 | 4 | 8 | Regulation of biological process |
| **442** | E2RN74 | Carboxypeptidase B2 | 49.1 | 11.996 | 5 | 9 | Metabolic process<br>Regulation of biological process |
| **443** | F6V0D8 | Chaperonin containing TCP1 subunit 2 | 57.4 | 11.992 | 4 | 9 | Cell organization and biogenesis<br>Metabolic process |

| | | | | | | | |
|---|---|---|---|---|---|---|---|
| | | | | | | | Regulation of biological process<br>Transport |
| **444** | E2QZT4 | Basigin (OK blood group) | 29.3 | 11.95 | 4 | 17 | Cellular component movement<br>Regulation of biological process |
| **445** | F1PTW4 | Serine/threonine kinase 24 | 49.5 | 11.93 | 5 | 13 | Cell communication<br>Cell organization and biogenesis<br>Metabolic process<br>Regulation of biological process<br>Response to stimulus |
| **446** | J9P7Y9 | Poly(RC) binding protein 1 | 37.4 | 11.929 | 4 | 12 | Metabolic process<br>Regulation of biological process |
| **447** | Q95N05 | Carboxylic ester hydrolase | 62 | 11.858 | 6 | 10 | Cell differentiation<br>Metabolic process |
| **448** | F1PRL5 | Solute carrier family 25 member 5 | 33 | 11.819 | 4 | 13 | Cell organization and biogenesis<br>Regulation of biological process<br>Transport |
| **449** | E2RGH5 | Ubiquitin like modifier activating enzyme 1 | 117.3 | 11.795 | 5 | 5 | Metabolic process<br>Response to stimulus |
| **450** | F1PHH6 | Guanine deaminase | 50.5 | 11.739 | 6 | 13 | Metabolic process |
| **451** | E2RMX9 | Aconitase 1 | 99.6 | 11.73 | 5 | 5 | Cellular homeostasis<br>Metabolic process<br>Response to stimulus |
| **452** | F1Q2J4 | Notch 3 | 216.2 | 11.722 | 4 | 2 | Cell differentiation<br>Development<br>Regulation of biological process<br>Response to stimulus |
| **453** | F1PLE7 | V-set domain containing T-Cell activation inhibitor 1 | 31.3 | 11.708 | 5 | 14 | Regulation of biological process<br>Response to stimulus<br>Transport |
| **454** | F1PPK3 | Solute carrier family 2, facilitated glucose Transporter member 3 | 54.3 | 11.698 | 3 | 8 | Transport |
| **455** | E2RQH0 | SH3 domain binding protein 4 | 107 | 11.667 | 4 | 4 | Regulation of biological process<br>Response to stimulus |
| **456** | E2RBL2 | Secretory carrier-associated membrane protein | 38.5 | 11.629 | 2 | 9 | Transport |

| | | | | | | | |
|---|---|---|---|---|---|---|---|
| **457** | G5D524 | MHC class II antigen | 10.7 | 11.535 | 4 | 39 | Response to stimulus |
| **458** | J9P5N9 | Discoidin domain receptor tyrosine kinase 1 | 104.4 | 11.502 | 4 | 4 | Cell growth<br>Cell organization and biogenesis<br>Cellular component movement<br>Metabolic process<br>Regulation of biological process<br>Response to stimulus |
| **459** | F1PM72 | Chromosome 2 open reading frame 72 | 29.3 | 11.477 | 4 | 19 | Regulation of biological process |
| **460** | F6X9C1 | Cathepsin H | 34.2 | 11.436 | 3 | 11 | Cell death<br>Metabolic process<br>Regulation of biological process<br>Response to stimulus |
| **461** | E2RD65 | Actin-related protein 2/3 complex subunit 5 | 12.1 | 11.371 | 2 | 27 | Cell organization and biogenesis<br>Cellular component movement<br>Regulation of biological process |
| **462** | E2RIU2 | Charged multivesicular body protein 2a | 25.1 | 11.346 | 5 | 23 | Cell organization and biogenesis<br>Regulation of biological process<br>Transport |
| **463** | E2R7L1 | Protein disulfide-isomerase A4 | 72.4 | 11.315 | 5 | 7 | Cellular homeostasis<br>Metabolic process<br>Regulation of biological process<br>Response to stimulus |
| **464** | F1PQT1 | Scavenger receptor class B member 1 | 51.6 | 11.218 | 5 | 11 | Cell organization and biogenesis<br>Cell proliferation<br>Cellular component movement<br>Metabolic process<br>Regulation of biological process<br>Response to stimulus<br>Transport |
| **465** | Q8MJD1 | Elastase, neutrophil expressed | 29.9 | 11.206 | 2 | 9 | Cellular component movement<br>Defense response<br>Metabolic process<br>Regulation of biological process<br>Response to stimulus<br>Transport |

| | | | | | | | |
|---|---|---|---|---|---|---|---|
| **466** | E2R542 | Hyaluronidase | 68 | 11.194 | 4 | 8 | Defense response<br>Metabolic process<br>Regulation of biological process<br>Response to stimulus |
| **467** | E2RRD1 | Protein kinase cAMP-dependent type II regulatory subunit alpha | 45.1 | 11.131 | 4 | 10 | Regulation of biological process |
| **468** | E2RS39 | Claudin | 28.4 | 11.096 | 4 | 14 | Cell organization |
| **469** | E2RCL7 | Phosphatidylinositol binding clathrin assembly protein | 77.4 | 11.019 | 4 | 6 | Cell organization and biogenesis<br>Cell proliferation<br>Metabolic process<br>Regulation of biological process<br>Response to stimulus<br>Transport |
| **470** | E2RHH4 | RAB3D, member RAS oncogene family | 25.4 | 10.981 | 4 | 14 | Metabolic process<br>Regulation of biological process<br>Response to stimulus |
| **471** | E2R3Q1 | Rac GTPase activating protein 1 | 72.3 | 10.952 | 5 | 7 | Cell organization and biogenesis<br>Cell proliferation<br>Regulation of biological process<br>Response to stimulus<br>Transport |
| **472** | E2QWU0 | Actin-related protein 2/3 complex subunit 4 | 19.5 | 10.936 | 4 | 23 | Cell organization and biogenesis<br>Regulation of biological process |
| **473** | Q9TSZ6 | Dystroglycan | 97.1 | 10.91 | 3 | 4 | Cell organization and biogenesis<br>Cellular component movement<br>Metabolic process<br>Regulation of biological process<br>Transport |
| **474** | F1P9U4 | Dihydropyrimidinase like 2 | 73.5 | 10.91 | 4 | 6 | Cell organization and biogenesis<br>Cellular component movement<br>Regulation of biological process |
| **475** | E2R7N5 | Coronin | 54.2 | 10.865 | 4 | 8 | Cell organization and biogenesis<br>Transport |
| **476** | F1PDT7 | Adaptor related protein complex 2 alpha 2 subunit | 95 | 10.853 | 4 | 5 | Transport |

| | | | | | | | |
|---|---|---|---|---|---|---|---|
| **477** | A0A1B1X469 | ATP synthase subunit alpha | 59.7 | 10.814 | 4 | 7 | Metabolic process<br>Transport |
| **478** | F1P6P2 | Transgelin | 22.4 | 10.802 | 4 | 19 | Cell differentiation |
| **479** | E2R7Z7 | Solute carrier family 38 member 3 | 55.6 | 10.786 | 4 | 8 | Cell communication<br>Regulation of biological process<br>Response to stimulus<br>Transport |
| **480** | E2QT78 | Transmembrane protein 79 | 43.7 | 10.738 | 4 | 11 | Cell death<br>Regulation of biological process<br>Transport |
| **481** | F6X637 | Karyopherin subunit beta 1 | 97.2 | 10.722 | 2 | 4 | Cell organization and biogenesis<br>Cellular component movement<br>Regulation of biological process<br>Response to stimulus<br>Transport |
| **482** | F1PYV1 | Methylenetetrahydrofolate dehydrogenase, cyclohydrolase and formyltetrahydrofolate synthetase 1 | 106.2 | 10.69 | 6 | 6 | Metabolic process |
| **483** | E2RJH1 | Ras-related C3 botulinum toxin substrate 2 (Rho family, small GTP binding protein Rac2) | 21.4 | 10.664 | 3 | 18 | Cell organization and biogenesis<br>Regulation of biological process<br>Response to stimulus |
| **484** | E2RK02 | Glycosylphosphatidylinositol specific phospholipase D1 | 92.8 | 10.661 | 4 | 6 | Cellular component movement<br>Metabolic process<br>Regulation of biological process<br>Response to stimulus |
| **485** | Q4LAL9 | Cathepsin D | 44.3 | 10.604 | 4 | 10 | Metabolic process |
| **486** | E2RNL3 | Peroxiredoxin 4 | 30.4 | 10.603 | 2 | 8 | Cell organization and biogenesis<br>Cellular homeostasis<br>Metabolic process<br>Regulation of biological process |
| **487** | E2RRQ7 | DEAD-box helicase 3, X-linked | 78.2 | 10.54 | 4 | 6 | Cell organization and biogenesis<br>Defense response<br>Metabolic process<br>Regulation of biological process<br>Response to stimulus |

| | | | | | | | |
|---|---|---|---|---|---|---|---|
| **488** | P62825 | GTP-binding nuclear protein RAN | 24.4 | 10.458 | 3 | 15 | Cell division<br>Regulation of biological process<br>Response to stimulus<br>Transport |
| **489** | F1PNG7 | Complement C1r | 81.7 | 10.442 | 5 | 6 | Metabolic process<br>Regulation of biological process<br>Response to stimulus |
| **490** | E2RDX2 | Charged multivesicular body protein 4b | 25 | 10.407 | 2 | 11 | Cell organization and biogenesis<br>Metabolic process<br>Regulation of biological process<br>Transport |
| **491** | F1Q0B2 | Mob kinase activator 1b | 25.5 | 10.399 | 3 | 16 | Regulation of biological process<br>Response to stimulus |
| **492** | F1PAB7 | Aldehyde dehydrogenase 9 family member A1 | 54 | 10.367 | 4 | 9 | Metabolic process |
| **493** | F1PD94 | Protease, serine 8 | 35.4 | 10.333 | 2 | 7 | Metabolic process<br>Regulation of biological process |
| **494** | E2RKA7 | Mitogen-activated protein kinase | 42 | 10.304 | 3 | 9 | Metabolic process<br>Regulation of biological process<br>Response to stimulus |
| **495** | J9P6K0 | RAP2A, member of RAS oncogene family | 20.8 | 10.303 | 3 | 11 | Cell organization and biogenesis<br>Regulation of biological process<br>Response to stimulus |
| **496** | E2R386 | Transmembrane protein 59 | 37.4 | 10.3 | 3 | 6 | Metabolic process<br>Regulation of biological process |
| **497** | J9P3M1 | LIM and SH3 protein 1 | 29.8 | 10.298 | 4 | 15 | Cellular component movement |
| **498** | E2QYD8 | Ribosomal protein L35A | 12.5 | 10.215 | 3 | 20 | Metabolic process |
| **499** | F1Q3J6 | Tyrosine-protein phosphatase non-receptor type | 68 | 10.198 | 4 | 7 | Cell differentiation<br>Cell organization and biogenesis<br>Cellular component movement<br>Metabolic process<br>Regulation of biological process<br>Response to stimulus |
| **500** | J9P4M6 | syntaxin 3 | 32.2 | 10.126 | 4 | 11 | Cell organization and biogenesis<br>Regulation of biological process<br>Transport |

| | | | | | | | |
|---|---|---|---|---|---|---|---|
| **501** | F1PAG5 | Vesicle amine Transport 1 | 31 | 10.087 | 2 | 11 | Metabolic process<br>Regulation of biological process |
| **502** | E2R071 | GIPC PDZ domain containing family member 1 | 35.9 | 10.086 | 3 | 11 | Cell communication<br>Cellular component movement<br>Regulation of biological process<br>Transport |
| **503** | F1PME4 | DnaJ heat shock protein family (Hsp40) member C3 | 56.8 | 10.06 | 5 | 9 | Metabolic process<br>Regulation of biological process<br>Response to stimulus |
| **504** | E2R002 | Prostaglandin reductase 1 | 35.7 | 10.059 | 5 | 20 | Metabolic process |
| **505** | G1K2D7 | Coagulation factor IX | 51.7 | 9.894 | 3 | 7 | Coagulation<br>Metabolic process |
| **506** | A0M8U6 | Testin | 48 | 9.888 | 4 | 9 | Regulation of biological process |
| **507** | E2RLP0 | RAB35, member RAS oncogene family | 23 | 9.839 | 3 | 17 | Regulation of biological process<br>Response to stimulus<br>Transport |
| **508** | E2R9U9 | FERM, ARH/RhoGEF and pleckstrin domain protein 2 | 117.3 | 9.834 | 5 | 4 | Cell differentiation<br>Cell organization and biogenesis<br>Regulation of biological process<br>Response to stimulus |
| **509** | F1P912 | Tropomyosin 1 | 37.4 | 9.826 | 4 | 9 | Cell organization and biogenesis<br>Regulation of biological process<br>Response to stimulus |
| **510** | E2QYW0 | OS9, endoplasmic reticulum lectin | 75.8 | 9.752 | 2 | 3 | Metabolic process<br>Response to stimulus |
| **511** | E2QYW3 | interleukin 22 receptor subunit alpha 2 | 26.9 | 9.742 | 4 | 20 | Regulation of biological process<br>Response to stimulus |
| **512** | F1Q080 | Tight junction protein ZO-3 | 113.5 | 9.728 | 5 | 5 | Regulation of biological process |
| **513** | F6XL96 | Von Willebrand factor A domain containing 1 | 45.6 | 9.713 | 3 | 9 | Cell organization and biogenesis<br>Response to stimulus |
| **514** | E2QUE7 | Ribosomal protein L26 | 17.2 | 9.69 | 4 | 22 | Metabolic process<br>Regulation of biological process<br>Response to stimulus |
| **515** | E2RHH5 | NHS like 2 | 123.7 | 9.656 | 4 | 4 | Cell differentiation |

| | | | | | | | |
|---|---|---|---|---|---|---|---|
| **516** | F1PYI3 | 2',3'-cyclic-nucleotide 3'-phosphodiesterase | 47.3 | 9.647 | 4 | 10 | Cell differentiation<br>Cell organization and biogenesis<br>Metabolic process<br>Response to stimulus |
| **517** | Q1HE58 | Ras-related protein Rab-27A | 25 | 9.629 | 4 | 16 | Cell differentiation<br>Cell organization and biogenesis<br>Coagulation<br>Regulation of biological process<br>Response to stimulus<br>Transport |
| **518** | E2RK07 | Exocyst complex component 1 | 102 | 9.572 | 4 | 4 | Regulation of biological process<br>Response to stimulus<br>Transport |
| **519** | F1Q4G4 | Peptidylglycine alpha-amidating monooxygenase | 113.4 | 9.442 | 4 | 3 | Metabolic process |
| **520** | F6Y8J4 | Protein tyrosine phosphatase type IVA, member 1 | 19.8 | 9.439 | 3 | 17 | Metabolic process<br>Regulation of biological process |
| **521** | F1PCY8 | adhesion G protein-coupled receptor G2 | 97.8 | 9.425 | 4 | 5 | Regulation of biological process<br>Response to stimulus |
| **522** | E2RH91 | Staphylococcal nuclease and tudor domain containing 1 | 101.9 | 9.419 | 4 | 4 | Cell differentiation<br>Metabolic process<br>Regulation of biological process |
| **523** | E2QWD3 | CRK proto-oncogene, adaptor protein | 42 | 9.417 | 3 | 9 | Cell organization and biogenesis<br>Regulation of biological process<br>Response to stimulus |
| **524** | E2QUU5 | 60 kDa heat shock protein, mitochondrial | 61 | 9.37 | 3 | 8 | Metabolic process<br>Response to stimulus |
| **525** | E2QUR2 | Glutamyl-prolyl-tRNA synthetase | 169.8 | 9.35 | 3 | 2 | Metabolic process<br>Regulation of biological process<br>Response to stimulus |
| **526** | F1P7Q9 | Nedd4 family interacting protein 2 | 35 | 9.334 | 2 | 7 | Regulation of biological process<br>Response to stimulus<br>Transport |
| **527** | E2R0H6 | Prolactin induced protein | 16.7 | 9.289 | 5 | 26 | Metabolic process<br>Regulation of biological process |

| | | | | | | | |
|---|---|---|---|---|---|---|---|
| | | | | | | | Response to stimulus |
| **528** | J9NVL9 | Peptidyl-prolyl cis-trans isomerase | 26.4 | 9.15 | 3 | 13 | Metabolic process<br>Regulation of biological process |
| **529** | E2R2A1 | Golgin a7 | 15.8 | 9.064 | 5 | 36 | Cell organization and biogenesis<br>Metabolic process<br>Regulation of biological process<br>Transport |
| **530** | J9NRZ2 | RAB18, member RAS oncogene family | 22.9 | 9.058 | 4 | 22 | Cell organization and biogenesis<br>Regulation of biological process<br>Response to stimulus<br>Transport |
| **531** | F1PB05 | Tsukushi, small leucine rich proteoglycan | 39 | 9.033 | 2 | 7 | Cell organization and biogenesis<br>Regulation of biological process<br>Response to stimulus |
| **532** | E2RAL0 | Rho GDP dissociation inhibitor beta | 22.8 | 9.028 | 2 | 14 | Regulation of biological process |
| **533** | J9P687 | Aldose 1-epimerase | 38 | 8.94 | 4 | 12 | Metabolic process |
| **534** | E2R599 | Carboxypeptidase Q | 52.1 | 8.913 | 3 | 6 | Metabolic process |
| **535** | F1Q3V2 | ATP-citrate synthase | 124.5 | 8.85 | 3 | 2 | Metabolic process |
| **536** | E2RFR0 | 40S ribosomal protein S8 | 24.2 | 8.827 | 3 | 18 | Metabolic process |
| **537** | E2R3G3 | Protein-L-isoaspartate O-methyltransferase | 30.3 | 8.816 | 3 | 11 | Metabolic process |
| **538** | J9NX46 | GLI pathogenesis related 2 | 17.1 | 8.728 | 3 | 22 | Regulation of biological process |
| **539** | E2RIQ8 | ribosomal protein S5 | 22.9 | 8.716 | 4 | 15 | Cell organization and biogenesis<br>Metabolic process<br>Regulation of biological process |
| **540** | P18066 | Ras-related protein Rab-5A | 23.6 | 8.694 | 3 | 15 | Cell organization and biogenesis<br>Metabolic process<br>Regulation of biological process<br>Response to stimulus<br>Transport |
| **541** | E2RLL6 | Chaperonin containing TCP1 subunit 6A | 58 | 8.679 | 5 | 9 | Metabolic process<br>Regulation of biological process |

| | | | | | | | |
|---|---|---|---|---|---|---|---|
| **542** | E2RK64 | Fermitin family member 3 | 75.7 | 8.675 | 4 | 8 | Cell organization and biogenesis<br>Regulation of biological process<br>Response to stimulus |
| **543** | E2RMA1 | Tumor protein, translationally-controlled 1 | 19.6 | 8.671 | 3 | 22 | Cell differentiation |
| **544** | J9JHH5 | Joining chain of multimeric IgA and IgM | 18.2 | 8.653 | 2 | 16 | Defense response<br>Regulation of biological process<br>Response to stimulus |
| **545** | F1PRE3 | Charged multivesicular body protein 6 | 21.1 | 8.607 | 2 | 14 | Cell organization and biogenesis<br>Regulation of biological process<br>Transport |
| **546** | E2QXE0 | Serine/threonine-protein phosphatase | 38.5 | 8.607 | 2 | 6 | Cell division<br>Metabolic process<br>Regulation of biological process<br>Response to stimulus |
| **547** | F1P9W6 | Neuraminidase 1 | 44.9 | 8.592 | 3 | 7 | Metabolic process |
| **548** | E2RFZ4 | STE20 like kinase | 143.5 | 8.58 | 3 | 5 | Cell organization and biogenesis<br>Metabolic process<br>Regulation of biological process<br>Response to stimulus |
| **549** | J9NVI2 | Bone marrow stromal Cell antigen 2 | 20.6 | 8.573 | 3 | 15 | Defense response<br>Regulation of biological process<br>Response to stimulus |
| **550** | F1P8X0 | Membrane palmitoylated protein 7 | 65.5 | 8.555 | 4 | 9 | Cell organization and biogenesis<br>Regulation of biological process |
| **551** | A0A0U5J457 | Tumor necrosis factor ligand 6A | 32.6 | 8.554 | 2 | 7 | Regulation of biological process<br>Response to stimulus |
| **552** | F1PBL4 | Fibrinogen alpha chain | 96.6 | 8.544 | 3 | 4 | Cell organization and biogenesis<br>Coagulation<br>Defense response<br>Metabolic process<br>Regulation of biological process<br>Response to stimulus |
| **553** | E2R8C9 | Mannosyl-oligosaccharide glucosidase | 92.3 | 8.543 | 2 | 3 | Metabolic process |

| | | | | | | | |
|---|---|---|---|---|---|---|---|
| **554** | J9NZ45 | RAP2B, member of RAS oncogene family | 16.9 | 8.513 | 4 | 16 | Regulation of biological process<br>Response to stimulus |
| **555** | F1PTW5 | Serine/threonine kinase 25 | 48.1 | 8.476 | 3 | 9 | Cell organization and biogenesis;<br>Metabolic process<br>Regulation of biological process<br>Response to stimulus |
| **556** | J9NW26 | EF-hand domain family member D1 | 18.5 | 8.463 | 3 | 20 | Cell organization and biogenesis |
| **557** | J9P5A8 | Serine/threonine-protein phosphatase | 38.6 | 8.442 | 2 | 6 | Metabolic process |
| **558** | F1PXN3 | Sialic acid acetylesterase | 68.4 | 8.418 | 3 | 6 | Metabolic process<br>Regulation of biological process |
| **559** | F6V9G5 | Syntaxin 4 | 34.3 | 8.342 | 3 | 9 | Cell organization and biogenesis<br>Regulation of biological process<br>Response to stimulus<br>Transport |
| **560** | F1PNS6 | Peptidase D | 43.2 | 8.314 | 3 | 6 | Metabolic process |
| **561** | E2R334 | Programmed Cell death 4 | 51.9 | 8.29 | 2 | 5 | Cell death<br>Regulation of biological process<br>Response to stimulus |
| **562** | E2QUL3 | Acyl-CoA synthetase long chain family member 1 | 77.9 | 8.287 | 3 | 5 | Metabolic process<br>Regulation of biological process<br>Response to stimulus<br>Transport |
| **563** | F1PSM2 | Alpha-1,4 glucan phosphorylase | 119.8 | 8.263 | 2 | 2 | Metabolic process |
| **564** | F1P624 | Cullin associated and neddylation dissociated 1 | 133.8 | 8.189 | 2 | 2 | Cell differentiation<br>Cell organization and biogenesis<br>Metabolic process<br>Regulation of biological process |
| **565** | J9P0W4 | Penta-EF-hand domain containing 1 | 29.6 | 8.179 | 2 | 9 | Cell organization and biogenesis<br>Metabolic process<br>Regulation of biological process<br>Response to stimulus<br>Transport |
| **566** | E2RHV1 | Semaphorin 4C | 92.4 | 8.123 | 5 | 6 | Cell differentiation<br>Cellular component movement<br>Regulation of biological process<br>Response to stimulus |

| | | | | | | | |
|---|---|---|---|---|---|---|---|
| **567** | F1PUL4 | Phosphoglucomutase 1 | 64.6 | 8.086 | 3 | 5 | Metabolic process |
| **568** | E2R4P4 | Transmembrane 4 L six family member 18 | 22.1 | 8.044 | 2 | 11 | Regulation of biological process<br>Transport |
| **569** | E2QU08 | Argininosuccinate synthase 1 | 46.6 | 8.033 | 4 | 8 | Metabolic process<br>Regulation of biological process<br>Response to stimulus |
| **570** | F1P7S9 | Serine/threonine-protein phosphatase | 38.6 | 8.021 | 2 | 8 | Metabolic process<br>Regulation of biological process<br>Response to stimulus |
| **571** | J9P1H1 | Utrophin | 394.6 | 7.95 | 3 | 1 | Regulation of biological process<br>Transport |
| **572** | E2R4H4 | Proteasome endopeptidase complex | 20 | 7.91 | 2 | 14 | Metabolic process |
| **573** | O77846 | MHC class II DLA-DQ beta chain b1 domain | 10.7 | 7.889 | 4 | 44 | Response to stimulus |
| **574** | E2R5Z5 | Transmembrane 9 superfamily member | 75.6 | 7.86 | 2 | 4 | Transport |
| **575** | J9JHV1 | PATJ, crumbs Cell polarity complex component | 199.5 | 7.86 | 3 | 2 | Cellular component movement |
| **576** | J9NWJ5 | Thioredoxin | 11.4 | 7.833 | 3 | 24 | Cellular homeostasis<br>Metabolic process<br>Regulation of biological process<br>Response to stimulus |
| **577** | P61008 | Signal peptidase complex subunit 3 | 20.3 | 7.789 | 2 | 12 | Metabolic process<br>Transport |
| **578** | E2R8W7 | Calcium binding protein 39 | 39.8 | 7.762 | 3 | 7 | Metabolic process<br>Regulation of biological process<br>Response to stimulus |
| **579** | F1P8T3 | Semaphorin 7A (John Milton Hagen blood group) | 63.6 | 7.755 | 2 | 4 | Cell differentiation<br>Cell growth<br>Cell organization and biogenesis<br>Regulation of biological process<br>Response to stimulus |
| **580** | Q5XNR9 | Leukemia inhibitory factor receptor | 123.6 | 7.743 | 3 | 2 | Regulation of biological process<br>Response to stimulus |

| | | | | | | | |
|---|---|---|---|---|---|---|---|
| **581** | E2R2H3 | ST6 beta-galactoside alpha-2,6-sialyltransferase 1 | 45.7 | 7.742 | 2 | 7 | Metabolic process |
| **582** | E2RL34 | 60S ribosomal protein L18a | 20.8 | 7.733 | 4 | 20 | Metabolic process |
| **583** | E2QY99 | Membrane palmitoylated protein 5 | 77.2 | 7.73 | 4 | 5 | Cell organization and biogenesis |
| **584** | E2R9K2 | Transmembrane protein 132A | 109.6 | 7.715 | 2 | 3 | Regulation of biological process<br>Metabolic process<br>Transport |
| **585** | E2R4F0 | Cadherin EGF LAG seven-pass G-type receptor 2 | 317.4 | 7.706 | 4 | 1 | Regulation of biological process<br>Response to stimulus |
| **586** | E2RAC6 | ATPase H+ Transporting V1 subunit B2 | 56.5 | 7.679 | 2 | 4 | Metabolic process<br>Transport |
| **587** | E2RJQ8 | V-type proton ATPase subunit | 40.4 | 7.671 | 3 | 8 | Cellular homeostasis<br>Response to stimulus<br>Transport |
| **588** | E2R569 | APC down-regulated 1 | 58.5 | 7.637 | 3 | 5 | Cellular component movement<br>Regulation of biological process |
| **589** | F1PVR0 | Matrix metalloproteinase | 54.2 | 7.624 | 3 | 5 | Cell differentiation<br>Metabolic process |
| **590** | Q30435 | Integral membrane protein | 23.5 | 7.621 | 3 | 16 | Response to stimulus |
| **591** | F1P7D2 | Syntaxin binding protein 1 | 67.5 | 7.552 | 3 | 5 | Cell communication<br>Cell organization and biogenesis<br>Regulation of biological process<br>Transport |
| **592** | E2R8A8 | 40S ribosomal protein S7 | 20.6 | 7.523 | 4 | 19 | Metabolic process<br>Regulation of biological process |
| **593** | A4GT58 | Cdc42 GTPase-activating protein | 50.6 | 7.443 | 3 | 7 | Regulation of biological process<br>Response to stimulus<br>Transport |
| **594** | F1PGM9 | Complement component 4 binding protein alpha | 57.9 | 7.41 | 3 | 7 | Regulation of biological process |
| **595** | E2RKP5 | Hepsin | 44.9 | 7.389 | 3 | 9 | Cell organization and biogenesis<br>Metabolic process<br>Regulation of biological process<br>Response to stimulus<br>Transport |

| | | | | | | | |
|---|---|---|---|---|---|---|---|
| **596** | F1P797 | 5-aminoimidazole-4-carboxamide ribonucleotide formyltransferase/IMP cyclohydrolase | 64.7 | 7.316 | 3 | 6 | Metabolic process |
| **597** | G1K291 | Palmitoyltransferase | 77.4 | 7.301 | 4 | 7 | Metabolic process |
| **598** | J9NZT7 | Myelin protein zero like 3 | 26.8 | 7.291 | 2 | 9 | Cell organization and biogenesis |
| **599** | Q9N0W3 | Occludin 1B | 63.4 | 7.29 | 4 | 8 | Cell organization and biogenesis<br>Metabolic process<br>Regulation of biological process<br>Response to stimulus |
| **600** | F1PFI3 | Phospholipase C beta 3 | 20.4 | 7.262 | 3 | 18 | Cellular homeostasis<br>Metabolic process<br>Regulation of biological process |
| **601** | E2RIM8 | Beta-hexosaminidase | 60.5 | 7.255 | 4 | 6 | Cell organization and biogenesis<br>Metabolic process<br>Regulation of biological process;reproduction<br>Response to stimulus |
| **602** | F1Q264 | Eukaryotic translation initiation factor 5A | 16.8 | 7.245 | 2 | 16 | Cell death<br>Metabolic process<br>Regulation of biological process<br>Transport |
| **603** | F6Y4A3 | MARCKS like 1 | 19.8 | 7.226 | 2 | 14 | Regulation of biological process |
| **604** | E2R6A6 | Eph receptor A1 | 107.4 | 7.172 | 3 | 4 | Cell organization and biogenesis<br>Metabolic process<br>Regulation of biological process<br>Response to stimulus |
| **605** | F1P969 | Eukaryotic translation initiation factor 3 subunit B | 92 | 7.147 | 3 | 3 | Cell organization and biogenesis<br>Metabolic process<br>Regulation of biological process |
| **606** | E2RIT2 | Transporter | 72 | 7.14 | 2 | 5 | Metabolic process<br>Response to stimulus<br>Transport |
| **607** | E2RGD9 | Myelin protein zero like 2 | 24.5 | 7.139 | 2 | 10 | Cell organization and biogenesis |
| **608** | E2RM44 | Beta-1,4-glucuronyltransferase 1 | 47.1 | 7.128 | 2 | 6 | Cellular component movement<br>Metabolic process |

| | | | | | | | |
|---|---|---|---|---|---|---|---|
| **609** | F1PT97 | Actin related protein 2/3 complex subunit 1A | 45 | 7.121 | 3 | 8 | Cell organization and biogenesis<br>Regulation of biological process |
| **610** | E2RPI9 | Clathrin light chain | 27.1 | 7.112 | 3 | 10 | Cell organization and biogenesis<br>Transport |
| **611** | E2R009 | Tax1 binding protein 1 | 94 | 7.111 | 4 | 4 | Cell organization and biogenesis<br>Metabolic process<br>Regulation of biological process |
| **612** | F1PY49 | Glutamine--fructose-6-phosphate transaminase 1 | 79.9 | 7.11 | 3 | 4 | Metabolic process<br>Regulation of biological process |
| **613** | J9P7K6 | Solute carrier family 2 member 9 | 60.2 | 7.102 | 3 | 5 | Metabolic process<br>Transport |
| **614** | E2RCY1 | Family with sequence similarity 49 member B | 36.7 | 7.084 | 3 | 10 | Regulation of biological process |
| **615** | F1PVU4 | V-type proton ATPase subunit A | 96.5 | 7.08 | 4 | 5 | Cell organization and biogenesis<br>Cellular homeostasis<br>Metabolic process<br>Regulation of biological process<br>Transport |
| **616** | E2QXH3 | Tubulointerstitial nephritis antigen like 1 | 52.3 | 7.057 | 2 | 5 | Metabolic process<br>Response to stimulus<br>Transport |
| **617** | F1PMM8 | Exocyst complex component 4 | 110.6 | 7.051 | 3 | 3 | Cell communication<br>Cell organization and biogenesis<br>Transport |
| **618** | J9P915 | Proteasome endopeptidase complex | 29.4 | 7.044 | 3 | 11 | Metabolic process |
| **619** | E2QS13 | Parkinsonism associated deglycase | 19.9 | 7.038 | 2 | 9 | Cell communication<br>Cell organization and biogenesis<br>Metabolic process<br>Regulation of biological process<br>Response to stimulus<br>Transport |
| **620** | E2RSH4 | Sushi domain containing 2 | 90 | 7.018 | 3 | 3 | Regulation of biological process<br>Response to stimulus<br>Transport |
| **621** | F1PGV3 | Exocyst complex component 7 | 83.9 | 7.016 | 3 | 4 | Regulation of biological process |

| | | | | | | | |
|---|---|---|---|---|---|---|---|
| | | | | | | | Transport |
| **622** | E2RC26 | Endonuclease, poly(U) specific | 47 | 7 | 3 | 7 | Metabolic process<br>Response to stimulus<br>Transport |
| **623** | E2R3D3 | Ribosomal protein L17 | 21.4 | 6.995 | 2 | 10 | Metabolic process |
| **624** | E2RRA0 | Claudin domain containing 1 | 37.2 | 6.984 | 2 | 7 | Cell organization and biogenesis<br>Transport |
| **625** | F1PKX2 | ABI family member 3 binding protein | 122.6 | 6.978 | 3 | 3 | Cell organization and biogenesis<br>Regulation of biological process |
| **626** | F6UZY1 | Adhesion G protein-coupled receptor G1 | 78.7 | 6.976 | 3 | 4 | Cellular component movement<br>Regulation of biological process<br>Response to stimulus |
| **627** | F1PF03 | Receptor protein-tyrosine kinase | 131.4 | 6.927 | 3 | 3 | Cell organization and biogenesis<br>Cell proliferation<br>Cellular component movement<br>Metabolic process<br>Regulation of biological process<br>Response to stimulus |
| **628** | J9P5H0 | Protein Wnt | 42.4 | 6.913 | 3 | 7 | Cell communication<br>Cell differentiation<br>Cell organization and biogenesis<br>Cell proliferation<br>Cellular component movement<br>Development<br>Metabolic process<br>Regulation of biological process<br>Response to stimulus |
| **629** | E2R5D6 | Galactocerebrosidase | 78.6 | 6.9 | 3 | 4 | Metabolic process<br>Regulation of biological process |
| **630** | F1PCG4 | Peroxiredoxin 2 | 21.9 | 6.871 | 2 | 15 | Cellular homeostasis<br>Metabolic process<br>Regulation of biological process<br>Response to stimulus |
| **631** | F1PAP5 | Spectrin alpha, non-erythrocytic 1 | 284.9 | 6.833 | 3 | 1 | Cell organization and biogenesis |
| **632** | F1PIF2 | Cathepsin Z | 29.3 | 6.813 | 3 | 10 | Metabolic process<br>Regulation of biological process |

| | | | | | | | |
|---|---|---|---|---|---|---|---|
| **633** | E2RFA4 | WW domain binding protein 2 | 28.5 | 6.813 | 3 | 12 | Regulation of biological process<br>Response to stimulus |
| **634** | E2RFF0 | Ras homolog enriched in brain | 20.5 | 6.792 | 3 | 16 | Regulation of biological process<br>Response to stimulus |
| **635** | F1Q331 | T-complex protein 1 subunit delta | 58 | 6.78 | 4 | 6 | Metabolic process<br>Regulation of biological process<br>Transport |
| **636** | F1P7C5 | Angiotensin-converting enzyme | 93.4 | 6.777 | 2 | 3 | Metabolic process<br>Regulation of biological process<br>Response to stimulus<br>Transport |
| **637** | P42929 | Heat shock protein beta-1 | 22.9 | 6.771 | 2 | 10 | Metabolic process<br>Regulation of biological process |
| **638** | E2RJ06 | 40S ribosomal protein SA | 35 | 6.77 | 2 | 7 | Cell organization and biogenesis<br>Metabolic process<br>Transport |
| **639** | E2RDN1 | Phosphoserine aminotransferase | 45.2 | 6.747 | 3 | 8 | Metabolic process |
| **640** | E2QV17 | Methionine sulfoxide reductase A | 25.9 | 6.729 | 3 | 18 | Metabolic process<br>Response to stimulus |
| **641** | E2R5F1 | Exocyst complex component 8 | 81.9 | 6.726 | 4 | 4 | Cell organization and biogenesis<br>Transport |
| **642** | F1PQ93 | Stratifin | 27.8 | 6.723 | 3 | 10 | Cell differentiation<br>Cell organization and biogenesis<br>Regulation of biological process<br>Response to stimulus |
| **643** | E2QXS7 | Adenosylhomocysteinase | 47.7 | 6.691 | 3 | 7 | Metabolic process<br>Transport |
| **644** | F1PI16 | 60S ribosomal protein L13a | 23.5 | 6.671 | 3 | 12 | Metabolic process<br>Regulation of biological process |
| **645** | E2RQ15 | Ras-related protein Rab-25 | 23.6 | 6.662 | 4 | 18 | Cell organization and biogenesis<br>Regulation of biological process<br>Response to stimulus<br>Transport |
| **646** | F1Q0K5 | Triokinase and FMN cyclase | 59.4 | 6.643 | 2 | 6 | Metabolic process<br>Regulation of biological process |

| | | | | | | | |
|---|---|---|---|---|---|---|---|
| **647** | E2RB81 | Chaperonin containing TCP1 subunit 5 | 59.5 | 6.56 | 3 | 4 | Metabolic process<br>Regulation of biological process<br>Response to stimulus<br>Transport |
| **648** | E2R667 | Coatomer subunit beta' | 102.3 | 6.547 | 2 | 2 | Transport |
| **649** | F1PW22 | Ethylmalonyl-CoA decarboxylase 1 | 32.5 | 6.541 | 3 | 10 | Metabolic process |
| **650** | F1P7M7 | Cell adhesion molecule 1 | 49.9 | 6.535 | 2 | 4 | Regulation of biological process<br>Response to stimulus |
| **651** | F1PVU3 | Non-specific serine/threonine protein kinase | 86.3 | 6.474 | 3 | 5 | Cell organization and biogenesis<br>Metabolic process<br>Regulation of biological process<br>Response to stimulus |
| **652** | E2RN10 | Beta-2-microglobulin | 14.3 | 6.467 | 3 | 18 | Cell differentiation<br>Metabolic process<br>Regulation of biological process<br>Response to stimulus<br>Transport |
| **653** | B1H0W0 | Cation-independent mannose-6-phosphate/insulin-like growth factor 2 receptor protein | 274.4 | 6.449 | 3 | 1 | Transport |
| **654** | E2QUL2 | Vesicle associated membrane protein 8 | 11.5 | 6.425 | 2 | 24 | Cell organization and biogenesis;<br>Regulation of biological process<br>Transport |
| **655** | J9P0R7 | Reticulon | 130.5 | 6.407 | 2 | 2 | Cell organization and biogenesis<br>Cellular component movement<br>Regulation of biological process |
| **656** | J9NV14 | Parvin alpha | 42.6 | 6.384 | 2 | 8 | Cell organization and biogenesis<br>Cellular component movement<br>Response to stimulus |
| **657** | F1PV63 | CUB domain containing protein 1 | 92.4 | 6.347 | 3 | 3 | Cell differentiation<br>Cellular homeostasis<br>Defense response<br>Development<br>Metabolic process<br>Regulation of biological process |
| **658** | J9NUA8 | Sperm equatorial segment protein 1 | 40.4 | 6.345 | 3 | 7 | Cell organization and biogenesis |

| | | | | | | | |
|---|---|---|---|---|---|---|---|
| | | | | | | | Transport |
| **659** | F1Q4G6 | Protease, serine 22 | 33.4 | 6.286 | 2 | 7 | Metabolic process |
| **660** | F1PQV4 | Olfactomedin like 3 | 46.2 | 6.261 | 2 | 4 | Regulation of biological process |
| **661** | E2RIA8 | Ribosomal protein L8 | 28 | 6.241 | 3 | 10 | Metabolic process |
| **662** | J9P9A6 | LHFPL tetraspan subfamily member 2 | 19.7 | 6.234 | 2 | 14 | Development |
| **663** | E2R8E8 | Deltex E3 ubiquitin ligase 2 | 67 | 6.186 | 3 | 5 | Regulation of biological process<br>Response to stimulus |
| **664** | E2R832 | Phosphomannomutase | 28.4 | 6.172 | 3 | 12 | Metabolic process<br>Transport |
| **665** | F1PQP8 | Ring finger protein 130 | 38.2 | 6.159 | 2 | 7 | Cell death<br>Metabolic process |
| **666** | F1P826 | Torsin family 2 member A | 36.1 | 6.13 | 2 | 11 | Cell organization and biogenesis<br>Metabolic process |
| **667** | L7N0I9 | D-3-phosphoglycerate dehydrogenase | 61 | 6.108 | 3 | 6 | Metabolic process |
| **668** | F2Z4P3 | 60S ribosomal protein L23 | 14.9 | 6.068 | 2 | 16 | Cell organization and biogenesis<br>Metabolic process<br>Regulation of biological process<br>Response to stimulus |
| **669** | J9P8M6 | Platelet derived growth factor D | 41.9 | 6.056 | 2 | 7 | Coagulation<br>Regulation of biological process;<br>Response to stimulus |
| **670** | F1PA63 | Lecithin-cholesterol acyltransferase | 49.7 | 6.043 | 2 | 5 | Cell organization and biogenesis<br>Metabolic process<br>Regulation of biological process<br>Transport |
| **671** | E2RSE6 | Furin, paired basic amino acid cleaving enzyme | 86.6 | 6.033 | 3 | 3 | Cell proliferation<br>Metabolic process<br>Regulation of biological process<br>Transport |
| **672** | E2RLZ9 | Abhydrolase domain containing 14b | 22.3 | 5.982 | 2 | 9 | Regulation of biological process |
| **673** | E2QXS1 | Lipase | 46 | 5.937 | 2 | 5 | Cell organization and biogenesis<br>Cell proliferation<br>Defense response<br>Metabolic process<br>Response to stimulus |

| | | | | | | | |
|---|---|---|---|---|---|---|---|
| **674** | Q8MJ44 | cAMP-dependent protein kinase catalytic subunit alpha | 40.5 | 5.93 | 3 | 7 | Metabolic process<br>Response to stimulus |
| **675** | J9P1M6 | Ubiquitin conjugating enzyme E2 V1 | 17.9 | 5.922 | 2 | 12 | Metabolic process<br>Regulation of biological process<br>Response to stimulus |
| **676** | E2RS84 | Dystrobrevin beta | 91.3 | 5.852 | 2 | 2 | Cell organization and biogenesis<br>Metabolic process<br>Transport |
| **677** | F1PQ10 | Integral membrane protein 2B | 27.4 | 5.842 | 2 | 12 | Regulation of biological process |
| **678** | E2RMQ5 | CD200 molecule | 33 | 5.839 | 2 | 8 | Regulation of biological process |
| **679** | F1PGJ5 | Scavenger receptor class B member 2 | 54.1 | 5.828 | 3 | 7 | Transport |
| **680** | G1K265 | Lysozyme | 16.5 | 5.827 | 2 | 14 | Defense response;Metabolic process<br>Response to stimulus |
| **681** | E2R872 | ATP binding cassette subfamily A member 9 | 183.9 | 5.811 | 3 | 2 | Transport |
| **682** | E2RHX6 | ARP1 actin related protein 1 homolog B | 42.3 | 5.781 | 3 | 7 | Regulation of biological process<br>Transport |
| **683** | E2RPE9 | Adipocyte plasma membrane associated protein | 46.3 | 5.78 | 2 | 5 | Metabolic process |
| **684** | J9P326 | Polyadenylate-binding protein | 70.6 | 5.727 | 3 | 4 | Regulation of biological process |
| **685** | F1PHX7 | KIAA1324 | 111.9 | 5.717 | 3 | 2 | Cell communication<br>Cell organization and biogenesis<br>Regulation of biological process<br>Response to stimulus |
| **686** | Q30410 | MHC class II DLA DQalpha chain | 28.3 | 5.713 | 3 | 10 | Response to stimulus |
| **687** | F1PVP5 | Reticulon | 112.8 | 5.683 | 3 | 3 | Cell organization and biogenesis |
| **688** | E2RT32 | Protein phosphatase 1 regulatory subunit 8 | 31.6 | 5.679 | 2 | 7 | Cell organization and biogenesis<br>Transport |
| **689** | F1P8Q0 | Integrin subunit alpha V | 112.5 | 5.678 | 2 | 2 | Cell differentiation<br>Cell growth<br>Cell organization and biogenesis<br>Cellular component movement<br>Metabolic process<br>Regulation of biological process |

| | | | | | | | |
|---|---|---|---|---|---|---|---|
| | | | | | | | Response to stimulus<br>Transport |
| **690** | E2RJC2 | Complement C1q C chain | 25.6 | 5.667 | 2 | 8 | Regulation of biological process<br>Response to stimulus |
| **691** | F1PHF0 | N-acetylgalactosamine-6-sulfatase | 58.1 | 5.651 | 2 | 3 | Metabolic process |
| **692** | Q004B2 | Carcinoembryonic antigen-related Cell adhesion molecule 1 isoform 4L | 56.4 | 5.643 | 3 | 6 | Development |
| **693** | F1PS26 | NADH-cytochrome b5 reductase | 35.6 | 5.577 | 2 | 6 | Metabolic process |
| **694** | F1P9Z4 | Related RAS viral (r-ras) oncogene homolog 2 | 20.6 | 5.572 | 3 | 17 | Cell differentiation<br>Regulation of biological process<br>Response to stimulus |
| **695** | F6Y470 | Serine incorporator 5 | 51.2 | 5.569 | 2 | 4 | Defense response<br>Metabolic process<br>Response to stimulus<br>Transport |
| **696** | F6XGU5 | KIAA0319 like | 104.9 | 5.567 | 3 | 3 | Development |
| **697** | F1P7I7 | Serine/threonine-protein phosphatase | 35.6 | 5.559 | 2 | 6 | Metabolic process |
| **698** | E2RCP9 | Proteasome 26S subunit, non-ATPase 2 | 100.2 | 5.552 | 3 | 3 | Metabolic process<br>Regulation of biological process |
| **699** | F1PXN6 | Aldehyde dehydrogenase 3 family member B1 | 58.4 | 5.451 | 2 | 4 | Metabolic process<br>Response to stimulus |
| **700** | F6X8D7 | Solute carrier family 2 member 1 | 55.2 | 5.447 | 2 | 4 | Cell communication<br>Cell organization and biogenesis<br>Response to stimulus<br>Transport |
| **701** | J9NTG9 | Eukaryotic translation initiation factor 3 subunit I | 32.8 | 5.446 | 2 | 7 | Metabolic process |
| **702** | E2RD14 | Pleckstrin homology domain containing A5 | 127.5 | 5.427 | 2 | 2 | Metabolic process |
| **703** | A0M8V0 | F-actin-capping protein subunit alpha-2 | 33 | 5.421 | 3 | 12 | Cell organization and biogenesis<br>Regulation of biological process |

| | | | | | | | |
|---|---|---|---|---|---|---|---|
| **704** | E2RL17 | Eukaryotic translation initiation factor 2 subunit alpha | 36.1 | 5.415 | 2 | 6 | Cell communication<br>Metabolic process<br>Regulation of biological process<br>Response to stimulus |
| **705** | E2QWS7 | Oncostatin M receptor | 108.9 | 5.408 | 3 | 3 | Regulation of biological process<br>Response to stimulus |
| **706** | E2R5Q1 | Calponin | 33.4 | 5.401 | 2 | 7 | Cell organization and biogenesis<br>Regulation of biological process<br>Response to stimulus |
| **707** | F1Q0S2 | Sodium/potassium-Transporting ATPase subunit beta | 24.9 | 5.393 | 3 | 13 | Cell organization and biogenesis<br>Cellular homeostasis<br>Regulation of biological process<br>Transport |
| **708** | E2RKR4 | Proteasome subunit alpha type | 28.4 | 5.375 | 2 | 8 | Metabolic process<br>Regulation of biological process |
| **709** | F1PWT3 | Deoxyribonuclease | 33.3 | 5.373 | 2 | 6 | Metabolic process |
| **710** | E2RKM9 | Calcium activated nucleotidase 1 | 43.1 | 5.373 | 2 | 5 | Metabolic process<br>Regulation of biological process<br>Response to stimulus |
| **711** | F1PU02 | Eukaryotic translation initiation factor 3 subunit F | 34.8 | 5.351 | 2 | 7 | Cell organization and biogenesis<br>Metabolic process<br>Regulation of biological process |
| **712** | E2RQP6 | Eukaryotic translation initiation factor 3 subunit C | 105.4 | 5.332 | 2 | 2 | Cell organization and biogenesis<br>Metabolic process<br>Regulation of biological process |
| **713** | E2R6K5 | Histone H3 | 15.3 | 5.297 | 2 | 13 | Cell organization and biogenesis |
| **714** | J9NTG7 | CD55 molecule (Cromer blood group) | 60.3 | 5.282 | 3 | 6 | Defense response<br>Regulation of biological process |
| **715** | E2R9R6 | Programmed Cell death 10 | 20.9 | 5.274 | 2 | 10 | Cell death<br>Cell organization and biogenesis<br>Cellular component movement<br>Regulation of biological process<br>Response to stimulus |
| **716** | E2RD90 | ST3 beta-galactoside alpha-2,3-sialyltransferase 4 | 37.7 | 5.218 | 2 | 6 | Metabolic process |

| | | | | | | | |
|---|---|---|---|---|---|---|---|
| **717** | E2RP88 | S100 calcium binding protein A14 | 11.4 | 5.198 | 2 | 21 | Defense response<br>Regulation of biological process<br>Response to stimulus |
| **718** | E2R7F3 | DnaJ heat shock protein family (Hsp40) member A2 | 45.8 | 5.143 | 2 | 4 | Metabolic process<br>Regulation of biological process<br>Response to stimulus |
| **719** | E2QXJ5 | V-type proton ATPase subunit A | 93 | 5.137 | 2 | 2 | Cell communication<br>Cell organization and biogenesis<br>Cellular homeostasis<br>Metabolic process<br>Response to stimulus<br>Transport |
| **720** | E2R7C8 | Tumor necrosis factor superfamily member 12 | 36.5 | 5.11 | 2 | 6 | Regulation of biological process<br>Response to stimulus |
| **721** | J9NSS7 | Leucine rich alpha-2-glycoprotein 1 | 39 | 5.087 | 2 | 5 | Cell differentiation<br>Regulation of biological process |
| **722** | F1PIA2 | Ubiquitin thioesterase | 31.4 | 5.082 | 2 | 7 | Metabolic process<br>Regulation of biological process<br>Response to stimulus |
| **723** | F1Q067 | Septin 7 | 44.8 | 5.041 | 2 | 5 | Cell division |
| **724** | F1PIG0 | Transforming growth factor beta receptor 3 | 94.2 | 5.025 | 2 | 3 | Cell organization and biogenesis<br>Metabolic process<br>Regulation of biological process<br>Response to stimulus |
| **725** | E2R0A6 | Ribosomal protein L28 | 15.7 | 4.973 | 2 | 15 | Metabolic process<br>Regulation of biological process |
| **726** | F1PPR9 | Solute carrier family 39 member 8 | 49.7 | 4.959 | 2 | 5 | Cellular homeostasis<br>Transport |
| **727** | P33711 | Somatotropin | 24.5 | 4.944 | 3 | 13 | Regulation of biological process<br>Response to stimulus |
| **728** | F1P9U5 | Solute carrier family 44 member 4 | 78.8 | 4.873 | 2 | 3 | Metabolic process<br>Regulation of biological process<br>Transport |
| **729** | E2QXF8 | Kappa-casein | 20.9 | 4.858 | 2 | 7 | Regulation of biological process<br>Transport |

| | | | | | | | |
|---|---|---|---|---|---|---|---|
| **730** | J9P0X7 | Coactosin like F-actin binding protein 1 | 13.8 | 4.823 | 2 | 14 | Defense response<br>Response to stimulus |
| **731** | F1PGX1 | Desmoglein 2 | 122.5 | 4.802 | 2 | 2 | Cell organization and biogenesis<br>Regulation of biological process |
| **732** | E2RPM8 | Tyrosine-protein kinase receptor | 155.7 | 4.761 | 2 | 2 | Cell organization and biogenesis<br>Metabolic process<br>Regulation of biological process<br>Response to stimulus |
| **733** | F1PBZ4 | NAD(P)H quinone dehydrogenase 1 | 30.8 | 4.748 | 2 | 7 | Metabolic process<br>Regulation of biological process<br>Response to stimulus |
| **734** | Q2Q423 | Adhesion G protein-coupled receptor E5 | 92.2 | 4.731 | 2 | 5 | Regulation of biological process<br>Response to stimulus |
| **735** | E2QX13 | Torsin family 4 member A | 46.4 | 4.711 | 2 | 4 | Metabolic process<br>Regulation of biological process<br>Response to stimulus |
| **736** | F6X725 | ATP binding cassette subfamily e member 1 | 67.4 | 4.701 | 3 | 5 | Cell organization and biogenesis<br>Metabolic process<br>Regulation of biological process<br>Transport |
| **737** | E2RB82 | Signal transducer and activator of transcription | 88 | 4.671 | 3 | 6 | Cell differentiation<br>Cell proliferation<br>Defense response<br>Metabolic process<br>Regulation of biological process;reproduction<br>Response to stimulus<br>Transport |
| **738** | F1PK68 | Ubiquitin domain containing 1 | 28.6 | 4.651 | 2 | 10 | Metabolic process<br>Regulation of biological process |
| **739** | J9P2W2 | Transforming growth factor beta induced | 71.8 | 4.617 | 3 | 4 | Cell organization and biogenesis<br>Cell proliferation |
| **740** | E2RBR6 | Proteasome subunit beta | 29.1 | 4.611 | 2 | 7 | Metabolic process<br>Regulation of biological process |
| **741** | E2RK13 | RP2, ARL3 GTPase activating protein | 39.3 | 4.607 | 2 | 5 | Cell organization and biogenesis<br>Metabolic process |

| | | | | | | | |
|---|---|---|---|---|---|---|---|
| | | | | | | | Regulation of biological process<br>Transport |
| **742** | F1P740 | Chloride intraCellular channel protein | 31.3 | 4.606 | 2 | 6 | Metabolic process<br>Regulation of biological process<br>Transport |
| **743** | F6Y290 | Leukotriene A(4) hydrolase | 78.7 | 4.584 | 3 | 4 | Metabolic process |
| **744** | J9P8S3 | Protein S100 | 15.5 | 4.584 | 2 | 10 | Defense response<br>Regulation of biological process<br>Response to stimulus |
| **745** | F1PWE6 | BAI1 associated protein 2 like 1 | 55.4 | 4.583 | 2 | 3 | Cell organization and biogenesis<br>Regulation of biological process<br>Response to stimulus |
| **746** | E2R0B6 | Proteasome subunit beta type | 25.5 | 4.571 | 2 | 9 | Metabolic process |
| **747** | E2RGI5 | Guanine nucleotide-binding protein subunit gamma | 7.3 | 4.549 | 2 | 24 | Regulation of biological process<br>Response to stimulus |
| **748** | F1PFM4 | Glutathione peroxidase | 25.4 | 4.479 | 2 | 9 | Metabolic process<br>Response to stimulus |
| **749** | F1PCE5 | Serpin family A member 1 | 46.7 | 4.476 | 2 | 4 | Regulation of biological process |
| **750** | F1PG99 | Methyltransferase like 9 | 31.9 | 4.444 | 2 | 8 | Metabolic process |
| **751** | F6V9M3 | Tetratricopeptide repeat domain 38 | 52.4 | 4.427 | 2 | 4 | Regulation of biological process |
| **752** | A7E3K7 | Cytochrome b-245 beta chain | 65.3 | 4.422 | 2 | 4 | Defense response<br>Metabolic process<br>Response to stimulus |
| **753** | F1PKA4 | MTSS1, I-BAR domain containing | 82.3 | 4.42 | 2 | 3 | Cell differentiation<br>Cell organization and biogenesis<br>Cell proliferation<br>Regulation of biological process<br>Response to stimulus |
| **754** | F1PCH0 | Sulfhydryl oxidase | 77.7 | 4.418 | 2 | 2 | Cellular homeostasis<br>Metabolic process<br>Regulation of biological process |
| **755** | E2RM54 | Polypeptide N-acetylgalactosaminyltransferase | 64.2 | 4.405 | 2 | 3 | Metabolic process |

| | | | | | | | |
|---|---|---|---|---|---|---|---|
| **756** | E2RTK6 | Charged multivesicular body protein 1a | 21.7 | 4.4 | 2 | 9 | Cell communication<br>Cell division<br>Cell organization and biogenesis<br>Metabolic process<br>Regulation of biological process<br>Response to stimulus<br>Transport |
| **757** | E2RPE5 | Ribosomal protein L35 | 14.6 | 4.393 | 2 | 15 | Metabolic process |
| **758** | E2QUN4 | Aminoadipate aminotransferase | 56.2 | 4.382 | 2 | 4 | Metabolic process |
| **759** | E2QRX3 | Protease associated domain containing 1 | 21.1 | 4.365 | 2 | 10 | Metabolic process<br>Regulation of biological process |
| **760** | J9NUD4 | Beta-1,4-galactosyltransferase 3 | 44.3 | 4.272 | 2 | 5 | Metabolic process |
| **761** | F1P7Y8 | Melanotransferrin | 72.6 | 4.27 | 2 | 3 | Regulation of biological process<br>Transport |
| **762** | F1PFZ9 | ADAM metallopeptidase domain 17 | 92.7 | 4.247 | 2 | 3 | Cellular component movement<br>Defense response<br>Metabolic process<br>Regulation of biological process<br>Response to stimulus |
| **763** | E2RFH1 | Transcobalamin 2 | 48 | 4.222 | 2 | 4 | Transport |
| **764** | F6UYJ9 | Calreticulin | 47.1 | 4.201 | 2 | 4 | Cell organization and biogenesis<br>Metabolic process<br>Regulation of biological process<br>Response to stimulus<br>Transport |
| **765** | E2RS87 | Gamma-aminobutyric acid type A receptor pi subunit | 50.6 | 4.177 | 2 | 4 | Regulation of biological process<br>Response to stimulus<br>Transport |
| **766** | F2Z4N5 | 40S ribosomal protein S11 | 18.4 | 4.177 | 2 | 12 | Metabolic process |
| **767** | L7N055 | Adenosylhomocysteinase | 70.2 | 4.175 | 2 | 3 | Metabolic process<br>Transport |
| **768** | E2R1J6 | Eukaryotic translation initiation factor 3 subunit E | 52.2 | 4.169 | 2 | 4 | Cell organization and biogenesis<br>Metabolic process<br>Regulation of biological process |
| **769** | J9NUZ6 | Peptidyl-prolyl cis-trans isomerase | 22 | 4.142 | 2 | 8 | Metabolic process |

| | | | | | | | |
|---|---|---|---|---|---|---|---|
| **770** | J9PBA0 | C1q and TNF related 5 | 25.9 | 4.128 | 2 | 11 | Cell organization and biogenesis<br>Transport |
| **771** | E2QYS4 | Pantetheinase | 57.9 | 4.125 | 2 | 4 | Metabolic process |
| **772** | E2R5U8 | Transthyretin | 15.9 | 4.113 | 2 | 17 | Metabolic process<br>Transport |
| **773** | F1PIY7 | Tyrosine-protein kinase | 133 | 4.1 | 2 | 2 | Cell differentiation<br>Cellular component movement<br>Defense response<br>Metabolic process<br>Regulation of biological process<br>Response to stimulus |
| **774** | Q95402 | Dqa1 protein | 19 | 4.097 | 2 | 6 | Response to stimulus |
| **775** | E2RE13 | ADP ribosylation factor like GTPase 8B | 21.5 | 4.095 | 2 | 10 | Regulation of biological process<br>Response to stimulus |
| **776** | F1PPJ6 | Torsin family 3 member A | 45.7 | 4.053 | 2 | 4 | Metabolic process<br>Regulation of biological process<br>Response to stimulus |
| **777** | E2RCN0 | Thiosulfate sulfurtransferase like domain containing 1 | 12.3 | 4.04 | 2 | 16 | Metabolic process<br>Regulation of biological process |
| **778** | F2Z4N3 | 60S ribosomal protein L27 | 15.8 | 4.033 | 2 | 15 | Metabolic process |
| **779** | E2RHR7 | Tyrosine--tRNA ligase | 63 | 4.026 | 2 | 3 | Metabolic process |
| **780** | A0A0A0MPE0 | 60S ribosomal protein L12 | 20.7 | 3.985 | 2 | 9 | Metabolic process |
| **781** | O18740 | Keratin, type I cytoskeletal 9 | 76.3 | 3.979 | 2 | 2 | Cell organization and biogenesis |
| **782** | E2RIV3 | Serine hydroxymethyltransferase | 53.4 | 3.972 | 2 | 3 | Cell organization and biogenesis<br>Metabolic process<br>Regulation of biological process |
| **783** | E2QZU4 | Inactive rhomboid protein | 98.2 | 3.972 | 2 | 3 | Cell proliferation<br>Cellular component movement<br>Metabolic process<br>Regulation of biological process<br>Transport |
| **784** | E2RJ26 | Proteasome 26S subunit, non-ATPase 11 | 47.4 | 3.954 | 2 | 4 | Cell differentiation<br>Cell organization and biogenesis<br>Metabolic process |
| **785** | F1Q409 | Copine 2 | 61 | 3.915 | 2 | 4 | Response to stimulus |

| | | | | | | | |
|---|---|---|---|---|---|---|---|
| **786** | E2RF16 | 5'-nucleotidase, cytosolic II | 64.9 | 3.891 | 2 | 5 | Metabolic process |
| **787** | E2QY31 | Platelet-activating factor acetylhydrolase IB subunit alpha | 46.6 | 3.853 | 2 | 6 | Cell communication<br>Cell differentiation<br>Cell division<br>Cell organization and biogenesis<br>Cell proliferation<br>Cellular component movement<br>Development<br>Metabolic process<br>Regulation of biological process<br>Response to stimulus<br>Transport |
| **788** | F1PFF2 | Protein S100 | 11.1 | 3.845 | 2 | 10 | Regulation of biological process |
| **789** | F1Q3C4 | Glutathione S-transferase mu 4 | 26.4 | 3.806 | 2 | 9 | Metabolic process |
| **790** | F6UT62 | Sorting nexin 3 | 18.8 | 3.75 | 2 | 9 | Cell organization and biogenesis<br>Regulation of biological process<br>Response to stimulus<br>Transport |
| **791** | J9NU25 | Heat shock protein family A (Hsp70) member 13 | 51.9 | 3.721 | 2 | 3 | Cell organization and biogenesis |
| **792** | E2RCW3 | Exocyst complex component 2 | 103.9 | 3.721 | 2 | 2 | Cell organization and biogenesis<br>Regulation of biological process<br>Transport |
| **793** | F1PW65 | Fibrinogen beta chain | 56.3 | 3.646 | 2 | 4 | Cell organization and biogenesis<br>Coagulation<br>Defense response<br>Metabolic process<br>Regulation of biological process<br>Response to stimulus |
| **794** | E2RD92 | Malectin | 32 | 3.642 | 2 | 9 | Metabolic process<br>Regulation of biological process |
| **795** | E2REF1 | Nucleotide binding protein like | 35.7 | 3.639 | 2 | 8 | Cell organization and biogenesis |
| **796** | E2RDE7 | Solute carrier family 7 member 4 | 68.5 | 3.634 | 2 | 4 | Transport |
| **797** | F1Q260 | Lysosomal associated membrane protein 1 | 44.6 | 3.617 | 2 | 5 | Regulation of biological process<br>Response to stimulus<br>Transport |

| | | | | | | | |
|---|---|---|---|---|---|---|---|
| **798** | J9P9X5 | Suppressor of tumorigenicity 14 protein homolog | 94.4 | 3.598 | 2 | 3 | Cell differentiation<br>Cell organization and biogenesis<br>Metabolic process |
| **799** | E2RGR7 | Soiled-coil domain containing 80 | 92.3 | 3.586 | 2 | 2 | Cell organization and biogenesis<br>Regulation of biological process |
| **800** | E2RPH7 | ExtraCellular matrix protein 2 | 78.6 | 3.541 | 2 | 2 | Cell organization and biogenesis<br>Regulation of biological process |
| **801** | E2QWL1 | Tropomodulin 3 | 39.6 | 3.54 | 2 | 5 | Cell organization and biogenesis<br>Regulation of biological process |
| **802** | F1Q104 | Calcium voltage-gated channel auxiliary subunit alpha2delta 1 | 119.6 | 3.534 | 2 | 2 | Regulation of biological process<br>Transport |
| **803** | F1P732 | Target of myb1 membrane trafficking protein | 53.6 | 3.528 | 2 | 3 | Transport |
| **804** | J9P4U9 | Dicarbonyl and L-xylulose reductase | 27.1 | 3.522 | 2 | 8 | Cell organization and biogenesis<br>Metabolic process |
| **805** | F6UZV8 | Calpain 1 | 82.1 | 3.506 | 2 | 2 | Metabolic process<br>Regulation of biological process |
| **806** | E2R5J7 | Receptor-type tyrosine-protein phosphatase | 89.6 | 3.465 | 2 | 2 | Metabolic process<br>Regulation of biological process<br>Response to stimulus |
| **807** | F2Z4Q7 | Serpin family F member 1 | 46.5 | 3.353 | 2 | 4 | Regulation of biological process |
| **808** | Q9MYU9 | Phospholipid hydroperoxide glutathione peroxidase | 10.7 | 3.347 | 2 | 21 | Metabolic process<br>Response to stimulus |
| **809** | E2QRV2 | TAO kinase 1 | 116 | 3.31 | 2 | 2 | Metabolic process<br>Regulation of biological process<br>Response to stimulus |
| **810** | F1PVU1 | Dolichyl-diphosphooligosaccharide--protein glycosyltransferase 48 kDa subunit | 50.5 | 3.244 | 2 | 4 | Metabolic process<br>Response to stimulus |
| **811** | E2RC55 | Pellino E3 ubiquitin protein ligase family member 2 | 42.4 | 3.243 | 2 | 6 | Metabolic process<br>Regulation of biological process<br>Response to stimulus |
| **812** | A1YV64 | Carcinoembryonic antigen-related Cell adhesion molecule 28 isoform 3L | 46.8 | 3.233 | 2 | 4 | Development |
| **813** | F1P7V6 | Stress induced phosphoprotein 1 | 62.7 | 3.23 | 2 | 3 | Cell growth |

| | | | | | | | |
|---|---|---|---|---|---|---|---|
| **814** | E2RSQ9 | Dihydropyrimidine dehydrogenase [NADP(+)] | 112 | 3.176 | 2 | 2 | Metabolic process |
| **815** | E2R484 | Keratin 85 | 55.3 | 3.174 | 2 | 4 | Cell organization and biogenesis<br>Response to stimulus |
| **816** | E2QTC7 | Peptidylprolyl isomerase D | 41.2 | 3.1 | 2 | 5 | Cell organization and biogenesis<br>Metabolic process<br>Regulation of biological process<br>Response to stimulus |
| **817** | E2RC02 | GDP-mannose 4,6-dehydratase | 34.1 | 3.083 | 2 | 6 | Metabolic process |
| **818** | J9P1G2 | Complement C1q B chain | 26.3 | 3.081 | 2 | 10 | Response to stimulus |
| **819** | E2RJA0 | p21 (RAC1) activated kinase 2 | 57.9 | 2.969 | 2 | 3 | Cell organization and biogenesis<br>Cellular component movement<br>Metabolic process<br>Regulation of biological process<br>Response to stimulus |
| **820** | E2RAN6 | Fructose-bisphosphatase 1 | 36.8 | 2.945 | 2 | 9 | Cell organization and biogenesis<br>Metabolic process<br>Regulation of biological process<br>Response to stimulus |
| **821** | E2RQ08 | Dolichyl-diphosphooligosaccharide--protein glycosyltransferase subunit 1 | 68.5 | 2.929 | 2 | 4 | Metabolic process |
| **822** | E2QU92 | Casein kinase 1 gamma 1 | 52.6 | 2.862 | 2 | 4 | Metabolic process<br>Regulation of biological process<br>Response to stimulus<br>Transport |
| **823** | F1PB08 | Septin 9 | 64.4 | 2.743 | 2 | 3 | Cell organization and biogenesis<br>Regulation of biological process |
| **824** | E2R7N3 | Midline 1 | 75.3 | 2.665 | 2 | 3 | Regulation of biological process |
| **825** | E2RQ71 | Apolipoprotein M | 21.4 | 2.652 | 2 | 11 | Cell organization and biogenesis<br>Metabolic process<br>Regulation of biological process<br>Transport |
| **826** | E2QZG2 | Ribosomal protein L23a | 17.7 | 2.362 | 2 | 12 | Cell organization and biogenesis<br>Cell proliferation<br>Metabolic process |
