# Supplementary material for: Canine colostrum exosomes: characterization and influence on the canine mesenchymal stem cell secretory profile and fibroblast anti-oxidative capacity
Source: BMC Vet Res. 2020 Nov 2;16:417. doi: 10.1186/s12917-020-02623-w (PMC7607682; doi:10.1186/s12917-020-02623-w)
Supplement: Supplementary file 3 — Additional file 3. List of specific proteins in common between CCM exosomes and canine MSC exosomes. [file 12917_2020_2623_MOESM3_ESM.pdf]

Additional file 3: List of specific proteins in common between CCM exosomes and canine MSCs exosomes.

Number	Accession	Protein	MW [kDa]	Score	Peptides	Coverage [%]	Biological Functions[1]
1	F1PFZ5	Milk fat globule-EGF factor 8 protein	47.8	1070.7	20	44.2	Cell organization and biogenesis<br>Regulation of biological process
2	L7N0G4	Tubulin alpha chain	50.1	628.2	12	38.1	Cell organization and biogenesis
3	E2QSF4	Tubulin beta chain	49.6	551.2	12	38.1	Cell organization and biogenesis
4	F1PTY1	Keratin, type II cytoskeletal 1	63.7	350.8	6	10.8	Defense response<br>Metabolic process<br>Regulation of biological process<br>Response to stimulus
5	F1PSK6	EH domain containing 1	53.1	267.5	5	13.0	Cell organization and biogenesis<br>Regulation of biological process<br>Transport
6	E2RQ14	Annexin	35.9	230.6	5	19.6	Regulation of biological process<br>Response to stimulus
7	J9P7X9	Gap junction protein	43.0	203.1	3	11.8	Cell communication<br>Regulation of biological process<br>Response to stimulus<br>Transport

[1] According to *Gene Ontology* parameters.

8	F2Z4P9	Ras-related protein Rab-10	22.5	189.5	4	22.5	Cell differentiation<br>Cell organization and biogenesis<br>Regulation of biological process<br>Response to stimulus<br>Transport
9	E2R8Z5	Keratin, type II cytoskeletal 5	62.7	149.7	3	5.4	Metabolic process
10	F1PZA1	Isocitrate dehydrogenase [NADP]	46.8	89.3	1	3.1	Metabolic process<br>Regulation of biological process<br>Response to stimulus
11	F1PGZ8	G protein-coupled receptor class C group 5 member B	43.0	82.7	1	3.1	Regulation of biological process<br>Response to stimulus
