# Supplementary figures and images for: Canine colostrum exosomes: characterization and influence on the canine mesenchymal stem cell secretory profile and fibroblast anti-oxidative capacity
Source: BMC Vet Res. 2020 Nov 2;16:417. doi: 10.1186/s12917-020-02623-w (PMC7607682; doi:10.1186/s12917-020-02623-w)

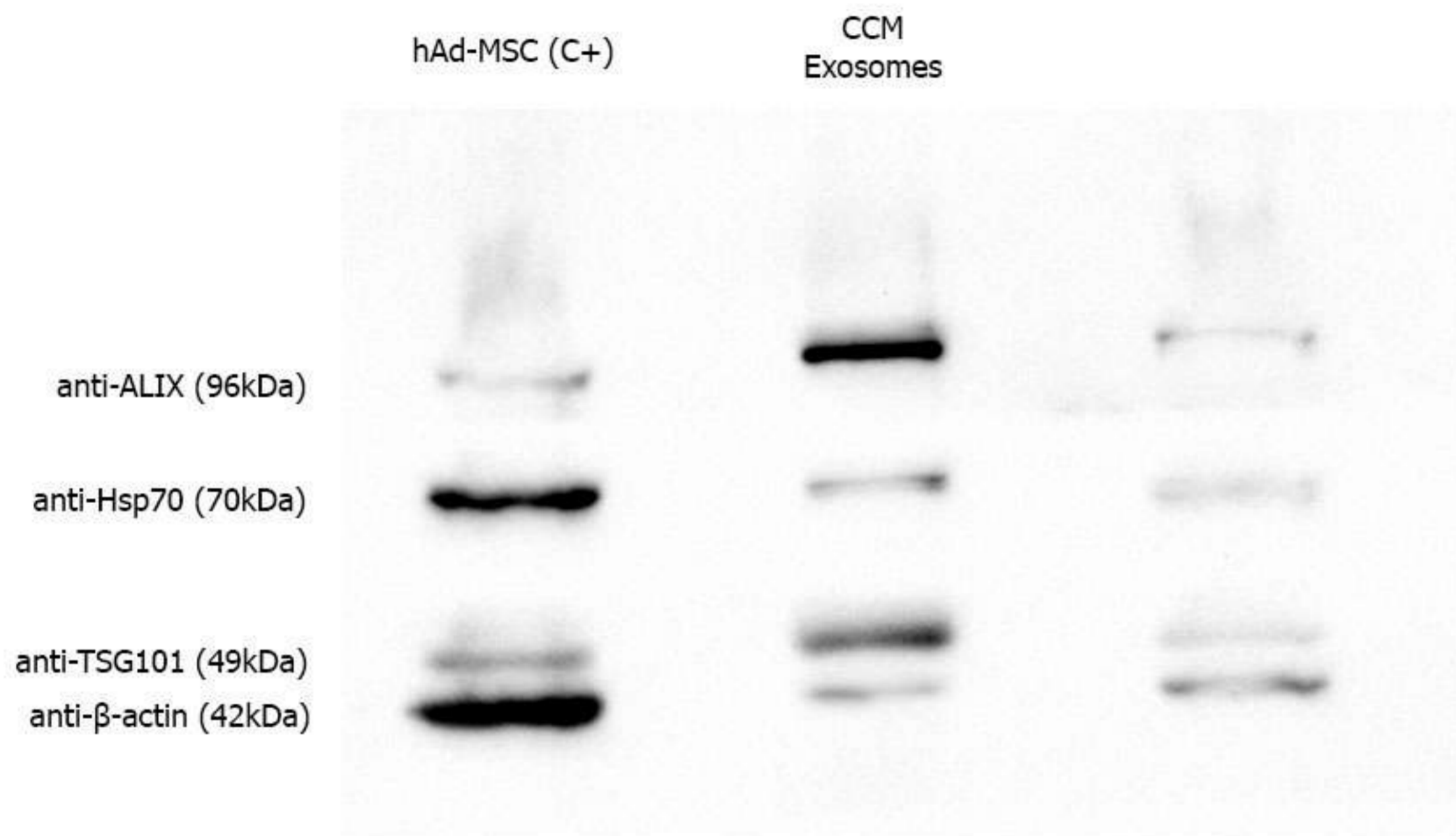

Exposure time: 20 seconds

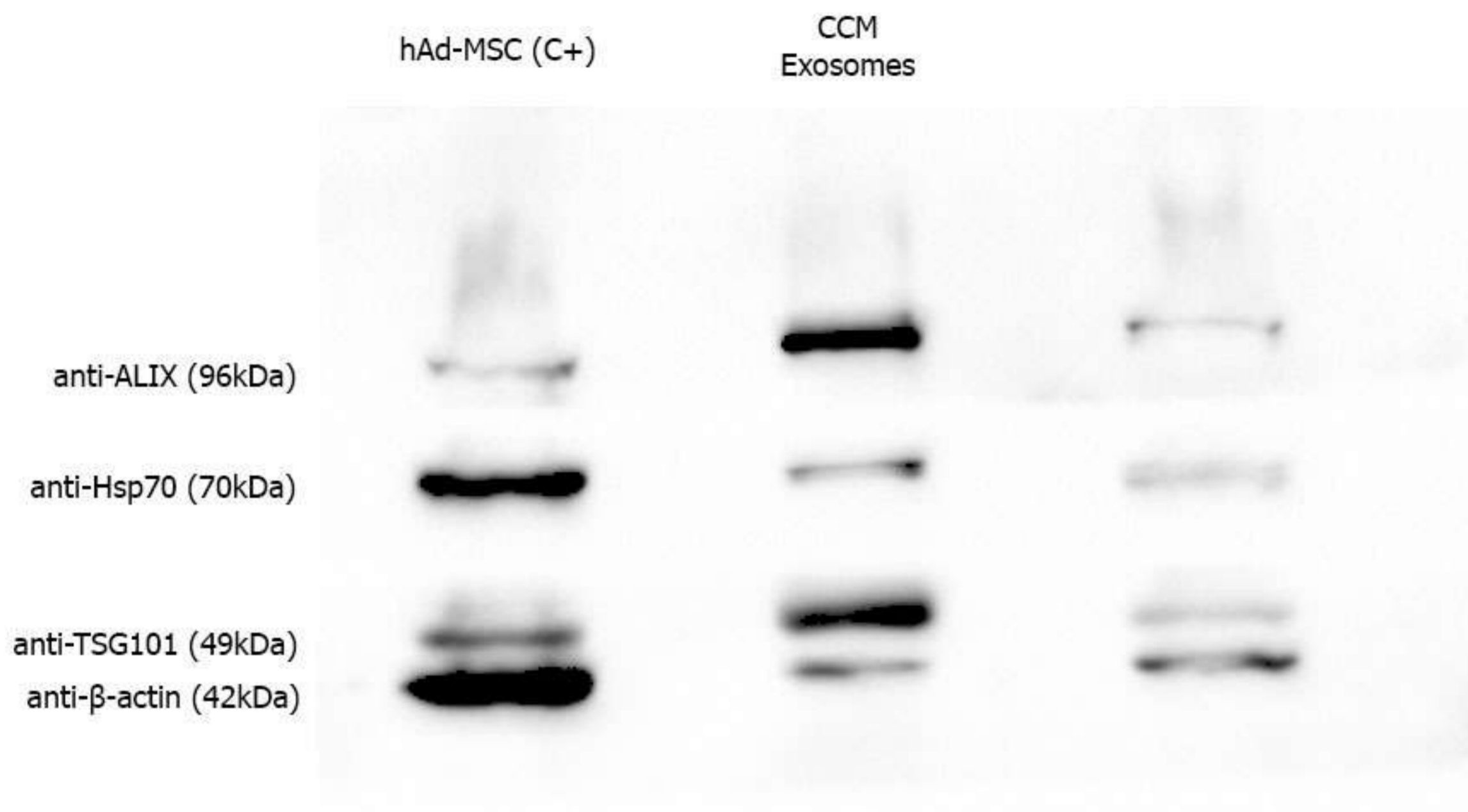

Exposure time: 40 seconds

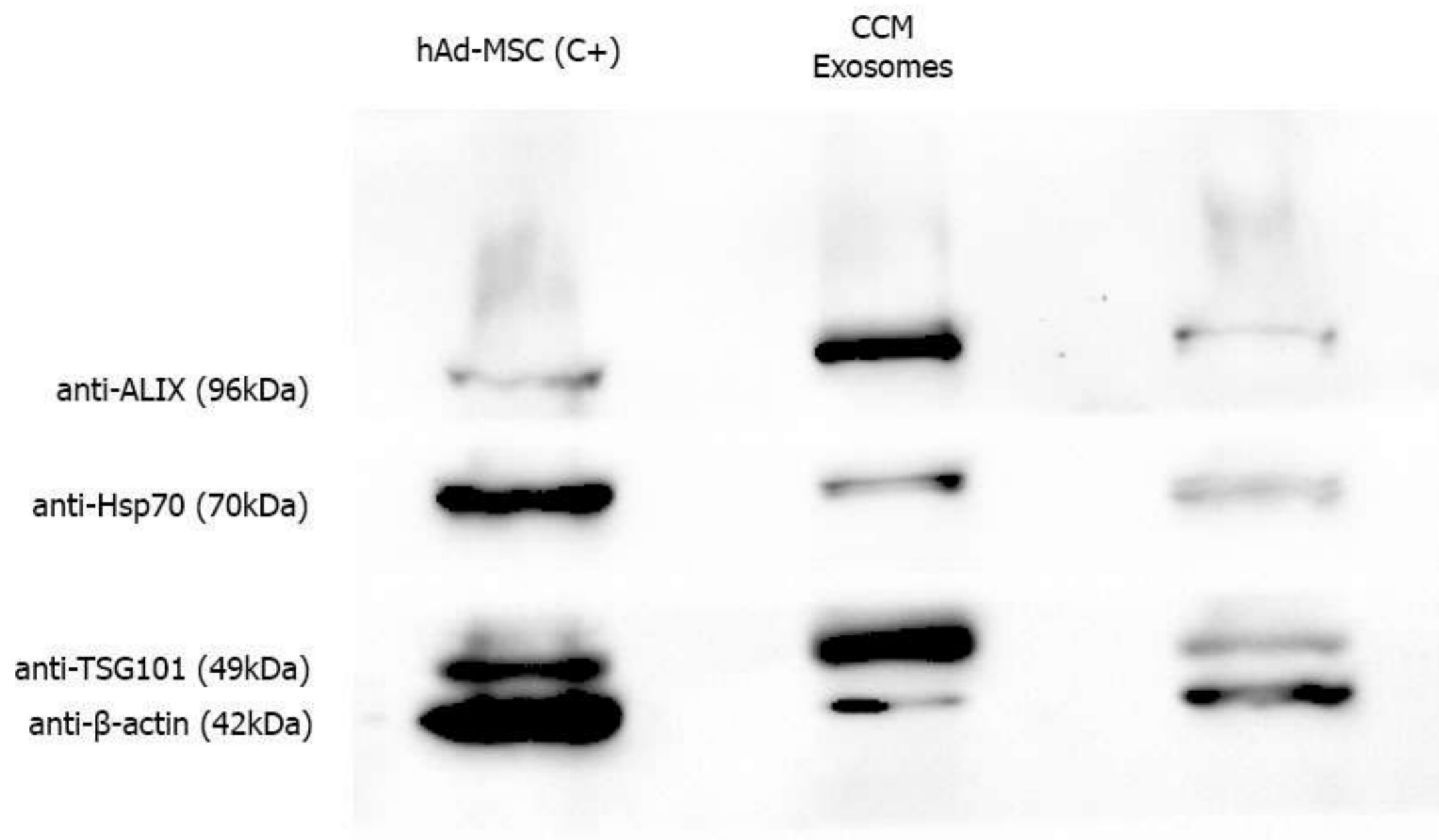

Exposure time: 60 seconds

Supplement: Supplementary file 4 — Additional file 4. Original Western Blot images with different exposure times and their descriptions. [file 12917_2020_2623_MOESM4_ESM.pdf]
